# Design and Synthesis of Novel Bis-Imidazolyl Phenyl Butadiyne Derivatives as HCV NS5A Inhibitors

**DOI:** 10.3390/ph15050632

**Published:** 2022-05-20

**Authors:** Jehad Hamdy, Nouran Emadeldin, Mostafa M. Hamed, Efseveia Frakolaki, Sotirios Katsamakas, Niki Vassilaki, Grigoris Zoidis, Anna K. H. Hirsch, Mohammad Abdel-Halim, Ashraf H. Abadi

**Affiliations:** 1 Department of Pharmaceutical Chemistry, Faculty of Pharmacy and Biotechnology, German University in Cairo, Cairo 11835, Egypt; jehad.hamdi93@gmail.com (J.H.); noura3110@gmail.com (N.E.); 2 Drug Design and Optimization, Helmholtz Institute for Pharmaceutical Research Saarland (HIPS)—Helmholtz Centre for Infection Research (HZI), Campus E8.1, 66123 Saarbrücken, Germany; mostafa.hamed@helmholtz-hips.de (M.M.H.); anna.hirsch@helmholtz-hips.de (A.K.H.H.); 3 Molecular Virology Laboratory, Department of Microbiology, Hellenic Pasteur Institute, Vas. Sofias Avenue, 11521 Athens, Greece; sevif@pasteur.gr (E.F.); nikiv@pasteur.gr (N.V.); 4 Division of Pharmaceutical Chemistry, Department of Pharmacy, School of Health Sciences, National and Kapodistrian University of Athens, Panepistimiopolis-Zografou, 15771 Athens, Greece; sotikats@pharm.uoa.gr; 5 Department of Pharmacy, Saarland University, Campus E8.1, 66123 Saarbrücken, Germany

**Keywords:** direct-acting antivirals, NS5A inhibitors, HCV antivirals, pan-genotypic activity, Gt1b molecular docking, diphenyldiyne core

## Abstract

In today’s global plan to completely eradicate hepatitis C virus (HCV), the essential list of medications used for HCV treatment are direct-acting antivirals (DAAs), as interferon-sparing regimens have become the standard-of-care (SOC) treatment. HCV nonstructural protein 5A (NS5A) inhibitors are a very common component of these regimens. Food and Drug Administration (FDA)-approved NS5A inhibitors, although very potent, do not have the same potency against all eight genotypes of HCV. Therefore, this study aims to synthesize NS5A inhibitor analogues with high potency pan-genotypic activity and high metabolic stability. Starting from an NS5A inhibitor scaffold previously identified by our research group, we made several modifications. Two series of compounds were created to test the effect of changing the length and spatial conformation (*para-para* vs. *meta-meta*-positioned bis-imidazole-proline-carbamate), replacing amide groups in the linker with imidazole groups, as well as different end-cap compositions and sizes. The frontrunner inhibits genotype 1b (Con1) replicon, with an EC_50_ value in the picomolar range, and showed high genotypic coverage with nanomolar range EC_50_ values against four more genotypes. This together with its high metabolic stability (t½ > 120 min) makes it a potential preclinical candidate.

## 1. Introduction

Hepatitis C infection remains a public health problem that is widespread throughout the globe. Despite the scientific advances witnessed during the past decade in both the diagnosis and treatment of hepatitis C virus (HCV) [1,2], more efforts are needed for the complete eradication of HCV, given that there is no availability of prevention measures, such as vaccines [3]. HCV is blood-borne and mainly transmitted through contaminated needle sharing and unscreened blood transfusions [4]. It belongs to the *Hepacivirus* genus of the family Flaviviridae and is a positive-sense single-stranded RNA virus [5]. The hepatitis C viral RNA encodes ten viral proteins that utilize both viral and cellular machinery to replicate the viral genome and create more viruses [1]. These proteins are divided into three structural proteins that form the viral particles (E1, E2, and core), the ion channel protein p7 required for virus assembly, and six non-structural (NS) proteins (NS2, NS3, NS4A, NS4B, NS5A, and NS5B) that are essential for viral RNA replication, as well as virion assembly [6,7].

HCV displays a relatively high degree of genetic heterogeneity and is classified into seven major genotypes (1–7), as well as a recently discovered genotype 8 [8,9]. All were identified by phylogenetic analysis of HCV strains isolated in various regions of the world. These genotypes are subdivided into ten subtypes. Genotypes 1a and 1b account for ~46% of all infections [10,11]. The hepatitis C virus is unique in its ability to establish a chronic infection, and unless successfully treated, it can lead to complications such as liver cirrhosis, hepatocellular carcinoma, and eventually liver failure [12].

Recent advances in the understanding of the different stages of the HCV life cycle and the proteins involved have made the development of specific antiviral drugs possible [13]. Three classes of direct-acting antivirals (DAAs) were developed to inhibit specific steps in the HCV replication cycle by targeting viral proteins, such as NS3/4A protease, NS5A protein, or NS5B polymerase [14].

In 2011, the first DAAs, telaprevir and boceprevir, of the class NS3/4A protease inhibitors (PIs), were approved by the Food and Drug Administration (FDA) [15]. They were first used in combination with interferon-α (IFN-α) and ribavirin (RBV), which resulted in higher sustained virological response (SVR) rates compared to IFN-α and RBV alone but still showed major limitations, and were hence withdrawn from the market in 2015 [16,17,18]. Nowadays, various DAAs used in interferon-free regimens have finished late-stage clinical development and have been approved after displaying improvement in efficacy, treatment duration, and tolerability. Approved DAAs, such as the NS3/4A protease inhibitors paritaprevir, simeprevir, and grazoprevir, the NS5A inhibitors daclatasvir, ledipasvir, ombitasvir, elbasvir, velpatasvir, and pibrentasvir, and the NS5B polymerase inhibitors sofosbuvir and dasabuvir, have led to a dramatic advancement in chronic Hepatitis C infection treatment [19]. Moreover, combination therapy regimens of DAAs with different mechanisms of action further reduced the drug resistance and increased the effectiveness of the antivirals, achieving sustained virologic response (SVR) rates over 90%, as well as reducing the treatment duration to 12 weeks, and even 8 weeks [20,21].

The HCV NS5A protein proved to be critical for viral viability, as its inhibition by DAAs was shown to produce the most rapid decline in viral load when compared to other HCV antiviral classes [22]. It has been proposed that this rapid decline in HCV RNA is due to the multifunctional role of NS5A in viral RNA replication, virion assembly, and secretion, in addition to creating an environment hospitable to the virus by modulating host-cell factors [23]. Today, NS5A inhibitors have become an integral part of nearly every approved IFN-free HCV therapy regimen to achieve a highly effective HCV treatment plan ensuring maximum potency combined with a higher barrier to resistance and pan-genotypic coverage [19].

Although DAAs have proven highly effective for the treatment of HCV, emerging resistance threatens this therapeutic advantage. Hence, this could be due to the virus RNA polymerase’s error-prone nature or due to genome geographical/ethnic diversity factors [2]. Thus, the need for new DAAs to overcome these obstacles is of utmost importance.

The NS5A protein has no identified enzymatic activity or known homologue in eukaryotes or prokaryotes, making the mechanism of action for the inhibitors unclear [24,25]. Moreover, until today, there is no available crystal structure of the protein complexed with any known inhibitor, making it difficult to use structure-based drug design approaches [26]. Therefore, based on the fact that NS5A inhibitors are most commonly symmetric peptidomimetics consisting of peptide-based caps connected to a linker (core), an attempt to improve drug positioning in the binding pocket and strengthen interaction forces was carried out by changing both the core and caps to possibly improve potency across genotypes and resistance profiles.

**Scheme 1:**
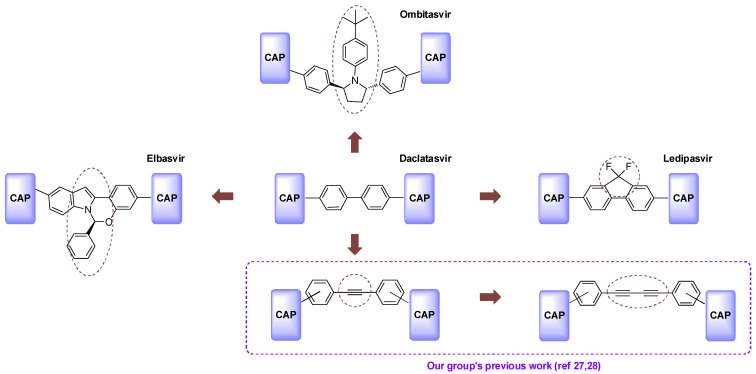
Comparative representation of biphenyl core modifications witnessed in various NS5A inhibitors.

Previous efforts of our group have resulted in highly active DAAs with picomolar EC_50_ values bearing a modified diphenylethyne and diphenylbutadiyne linker with amide-mediated L-proline-L-valine carboxylate caps (Scheme 1) [27,28]. In the present work, we report the structural modification of a series of DAA compounds that introduce the more elongated diphenylbutadiyne linker by focusing on cap modification. Specifically, the proposed changes will introduce a 3,3′-(buta-1,3-diyne-1,4-diyl)-dianiline and 4,4′-(buta-1,3-diyne-1,4-diyl)dianiline scaffolds [28] with bioisosteric replacement of the amide linkages to the caps with imidazoles to enhance both metabolic stability and potency. Indeed, the imidazole ring serves as a bioisostere of the amide bond, as it was previously reported to mimic its action with improved bioavailability, as well as enhanced resistance to metabolic cleavage by proteases, hydrolysis, and oxidation [29]. Therefore, imidazole represents one of the most useful heterocyclic rings in medicinal chemistry. It is capable of serving as a ligand for a diverse array of receptors by mimicking the structure of peptides and binding reversibly to enzymes, providing incredible opportunities for drug discovery [30,31,32,33]. Additionally, different end-capping groups are introduced, creating two sets of symmetric compounds with the capping groups attached either in *meta-meta* or *para-para* substitution connected to the core.

## 2. Results and Discussion

### 2.1. Design Concept

All reported NS5A inhibitors have a long-established design of highly symmetrical pharmacophore structures, commonly bearing bis-pyrrolidine caps along with other structural features, such as linker length, as well as substituents attached to the caps that affect target activity and spectrum [34].

**Figure 1:**
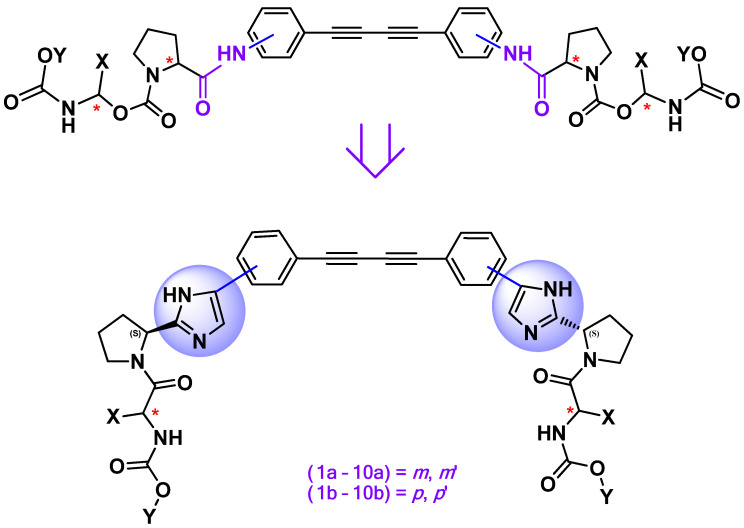
Planned modifications described herein. * = *R* or *S*.

In the pursuit of further developing our previously reported NS5A inhibitors having the 3,3′-(buta-1,3-diyne-1,4-diyl)dianiline and 4-[4-(4-aminophenyl)buta-1,3-diyn-1-yl]aniline cores as a starting point [28], amide groups adjacent to the linker were replaced with imidazole rings, as shown in Figure 1. In some cases of already existing drugs, the change of amide to imidazole also enhanced oral bioavailability, therapeutic window, or even enabled the preparation of injectable aqueous-soluble salts because of the imidazole nitrogen basicity [29].

In compound series (**1a**–**10a**), Figure 1, the imidazole-proline-carbamate caps were connected to the core at *meta-meta* positions, while in compound series (**1b**–**10b**) they were connected to the core at *para-para* positions, in an attempt to reach the ideal length and the most promising spatial orientation. Additionally, we planned to investigate the effect of cap chemical composition and stereochemistry on the potency of synthesized compounds, as it affects the compound’s conformation in space, as well as its interactions with the protein. We investigated the effect of the most commonly used amino acid carbamates caps, such as *L/D*-valine, *L*/*D*-leucine, and *L/D*-phenyl glycine, on the potency of the compounds, while the terminal alkyl carbamate moiety varied between methyl, ethyl, and butyl.

### 2.2. Synthesis

**Scheme 2:**
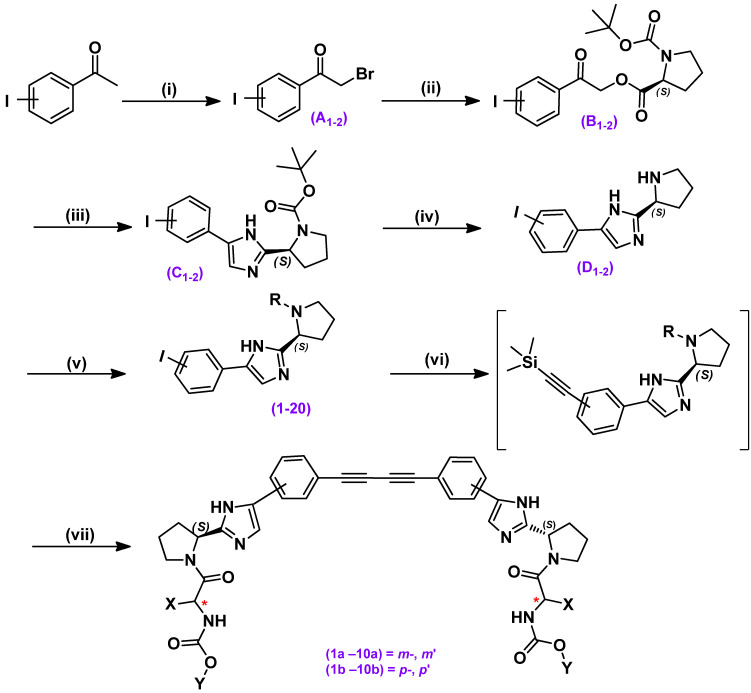
General synthetic route of DAAs, reagents, and conditions; (i) NBS, *p*-TsOH, acetonitrile, reflux, 2h; (ii) *N*-Boc-L-proline, TEA, acetonitrile, room temperature, 3 h; (iii) Ammonium acetate, toluene, reflux, 16 h; (iv) TFA, room temperature, 3 h; (v) Appropriate carbamate (**1c**–**10c**), HBTU, TEA, DCM, room temperature, 2 h; (vi) PdCl_2_(PPh_3_)_2_, CuI, DMF, under argon, 70 °C; (vii) K_2_CO_3_, copper (I) iodide (10% mmol), distilled water, overnight, 55 °C. * = *R* or *S*.

The synthesis of the desired compounds was carried out in seven steps, as summarized in Scheme 2. First, the appropriate iodo-acetophenone was brominated using *N*-bromosuccinimide (NBS) under acidic conditions in the presence of *p*-TsOH, where the bromo substituent was installed in the alpha position to the carbon atom then replaced by an *N*-Boc-*L*-proline residue using triethylamine (TEA) in acetonitrile. The produced ketoester (B_1–2_) was used for the formation of the imidazole ring through the reaction with ammonium acetate in refluxing toluene. After that, the deprotection of Boc takes place by trifluoracetic acid (TFA) to give the free amine moiety (D_1–2_). On the free amine, coupling with amino acid carbamates (**1c**–**10c**) took place using HBTU as a coupling agent to get the monomers of the desired dimeric final compounds. Monomeric compounds then underwent Sonogashira cross-coupling reaction with trimethylsilyl acetylene (TMSA) to replace the iodo group, where triphenylphosphine palladium (II) chloride ([PdCl_2_(PPh_3_)_2_]) was used as a catalyst and copper (I) iodide (CuI) as a co-catalyst in the presence of TEA as a base, all together in DMF as solvent under inert conditions at 70 °C. Finally, the desilylation along with dimerization step took using K_2_CO_3_ in methanol and distilled water in the presence of a few specks of CuI to afford the final dimeric compounds (Table 1).

**Scheme 3:**
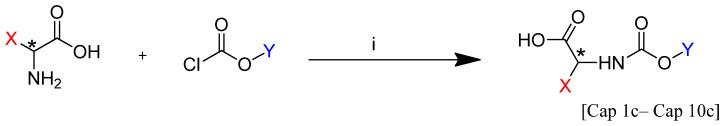
Reagents and conditions: (i) NaOH/1,4-dioxane, room temperature, overnight. * = *R* or *S*.

As for the preparation of the required intermediate carbamates (**1c**–**10c**, summarized in Table 2), amino acids and chloroformates were treated under Schotten–Baumann conditions in the presence of NaOH and dioxane as shown in Scheme 3.

### 2.3. Biological Activity

All synthesized compounds were tested against HCV genotype 1b Con-1 subgenomic replicon in the Huh-5-2 stable cell line. Specifically, their activity against viral replication was estimated by measuring firefly luciferase, which is co-expressed by the bicistronic replicon, and their half-maximal effective concentrations (EC_50_) were determined. In parallel, the half-maximal cytotoxic concentrations (CC_50_) of the compounds were estimated, and their selective indexes (SI_50_: CC_50_/ EC_50_) were calculated. All novel synthesized compounds were active against HCV 1b with EC_50_ < 1 µM except for compound **4b** (Table 3).

#### 2.3.1. Structure–Activity Relationship

Evaluating the structure–activity relationship (SAR) of the synthesized compounds showed that the structure and stereochemistry of the capping groups have a major influence on the activity (EC_50_).

Regarding the amino acid carbamate derivatives, both aliphatic and aromatic amino acids were used. The aliphatic amino acids were valine and leucine. All leucine derivatives were more potent than their corresponding valine derivatives, irrespective of the stereochemistry of the amino acids. This can be seen in both compound series, the *m*, *m*’ connected caps (**1a**–**10a**) and the *p*, *p*’ connected caps (**1b**–**10b**), indicating that the extra methylene spacer in leucine increased the lipophilicity/size of the terminal side chain and enhancing the activity. This is exemplified by compounds **5a**–**8a** to compounds **1a**–**4a**, respectively, and compounds **5b**–**8b** to compounds **1b**–**4b**, respectively.

Comparing the effect of using aromatic amino acids, e.g., phenylglycine, to that of aliphatic amino acids, phenylglycine derivatives showed higher activity than both valine and leucine derivatives in series (**1a**–**10a**). Therefore, the order of activity for amino acids in capping groups for this series is phenylglycine > leucine > valine. However, in series (**1b**–**10b)**, the aliphatic caps showed activity better than the aromatic ones, where leucine derivatives showed the highest activity. This is surprising, as phenylglycine derivatives were reported to have EC_50_ values lower than the valine derivatives when adopted with other cores [35,36].

As for the stereochemistry of the terminal amino acids used, in series (**1a**–**10a**), *D*-enantiomers showed higher biological activity than *L*-enantiomers. This can be seen when comparing the results of *D*-enantiomers **6a** (EC_50_ = 96.6 nM), **8a** (EC_50_ = 85.4 nM), and **10a** (EC_50_ = 0.1 nM), to their corresponding *L*-enantiomers **5a** (EC_50_ = 113.7 nM), **7a** (EC_50_ = 97.8 nM), and **9a** (EC_50_ = 62.0 nM), respectively (Table 3). Compound **10a** in particular, the *D*-phenylglycine derivative, is by far the most potent of all tested compounds with an EC_50_ value of 0.1 nM, as well as being the safest with the highest SI_50_ > 9990. The only exception to the stereochemistry pattern is the methyl-valine analogues, where the *L*-valine methyl derivative **1a** is two times better in activity than the *D*-valine methyl derivative **2a**. In series (**1b**–**10b**), the *L*-enantiomers were found to be more active than the corresponding *D*-enantiomers, except for the *D*-phenylglycine methyl **10b**, which showed better activity than the *L* analogue.

Concerning the terminal carbamate *O*-substituents of series (**1a**–**10a**), the *L*-valine with ethyl substituent **3a**, (EC_50_ = 158.8 nM) had almost equal activity to the methyl substituent **1a** (EC_50_ = 166.4 nM), while the butyl substituent **4a** showed much lower activity than both (EC_50_ = 901.8 nM). In the case of *L*- and *D*-leucine capping, the ethyl substituent again showed almost equivalent activity to that of methyl. Therefore, the order of activity for *O*-substituents is methyl ≈ ethyl > butyl.

For series (**1b**–**10b**), the terminal leucine caps showed an increase in activity with a decrease in the bulkiness of their terminal *O*-substituents. The *L*-leucine methyl **5b** showed activity higher than *L*-leucine ethyl **7b** by more than 3-fold, while *D*-leucine methyl **6b** showed almost double the potency of *D*-leucine ethyl **8b**. Regarding valine caps, the activity increased more clearly as the bulkiness of the terminal *O*-substituent decreased. The *L*-valine methyl **1b** showed higher activity than the *L*-valine ethyl **3b** and *L*-valine butyl **4b**. Therefore, the order of activity for *O*-substituents is methyl > ethyl > butyl.

Overall, compound **10a** showed the highest activity with an EC_50_ value of 0.1 nM among *m*, *m’*-compound series (**1a**–**10a**), while compound **5b** showed the highest activity with an EC_50_ value of 16.1 nM among the *p*, *p*-compound series (**1b**–**10b**).

When comparing series (**1a**–**10a**) with *m*, *m*’ connected caps to series (**1b**–**10b**) with *p*, *p*’-connected caps*,* the *m*, *m’*-connection exhibited higher activity when combined with the D-enantiomer terminal caps, while the *p*, *p’* connected compounds favored the L-enantiomer terminal caps. The *m*, *m’* compounds were more potent with the aromatic caps, while the *p*, *p’* showed higher potency with the bulky aliphatic terminal groups. As for the carbamate *O*-substituted terminal group, the activity for both *m*, *m’*- and *p*, *p’*-compounds was enhanced when combined with shorter substituents, such as the methyl group.

As per the previous observations, we cannot conclude that there are common patterns that can tell which series shows superior activity over the other; however, the most potent among all compounds was the *m*, *m’* derivative with the D-enantiomer aromatic cap and the methyl terminal group (compound **10a**).

#### 2.3.2. Molecular Modeling

**Figure 2:**
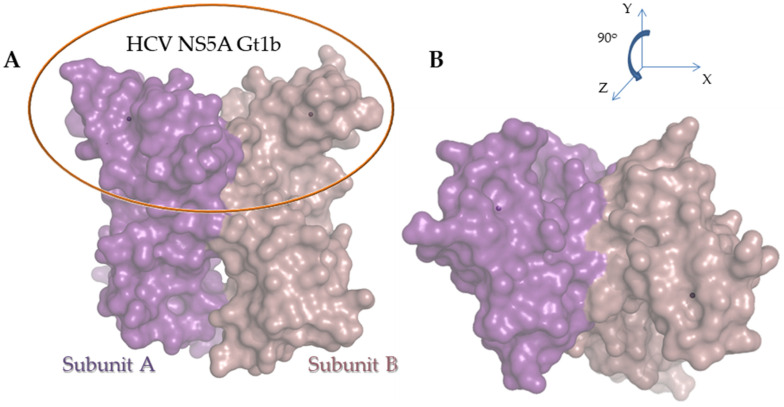
NS5A dimer protein surface representation: (**A**) AB dimer is shown as a surface with subunit A colored purple, subunit B colored lilac and an orange ellipse highlighting the interface forming between the two, (**B**) 90° protein rotation of the AB dimer among the Y and Z axis, revealing the whole interface of the *N*-terminus.

In addition to the SAR data extracted herein, we tried rationalizing the activity of all our compounds using docking experiments performed over HCV NS5A Gt1b (Con1) protein (PDB entry 3FQM) [37]. As described previously by our group [27] and others [38,39], a possible region of interaction for DAAs is formed between the dimeric protein chain interface at the *N*-terminus where zinc metal ions also reside (Figure 2). Here, we focus on analyzing the results of the most active compound (i.e., **10a**) vs. daclatasvir. Poses and binding modes for other compounds are available in the supporting information.

**Figure 3:**
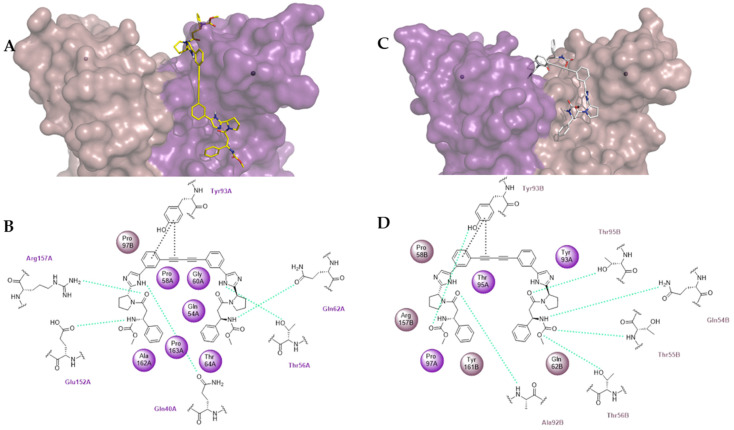
(**A**) Purple and violet surface representation of the HCV GT 1b subunits A and B, respectively, in the presence of **10a** at binding mode obtained from OpenEye the software as yellow sticks and zinc metal shown as faded balls, (**B**) 2D representation of **10a** binding network as obtained from OpenEye software. Residues of purple and violet colors are for subunits A and B, respectively. Green dashed lines represent HB between protein and **10a**, whilst black dashed lines refer to *π*-*π* interactions. (**C**) Purple and violet surface representation of the HCV GT 1b subunits A and B, respectively, bound to **10a**; pose predicted by the software PyRx as gray sticks and zinc metal shown as faded balls; (**D**) 2D representation of **10a**; binding network predicted by PyRx software. Residues of purple and violet color are for subunits A & B, respectively. Green dashed lines represent HB between protein and **10a**, while black dashed lines refer to *π*-*π* interactions.

**Figure 4:**
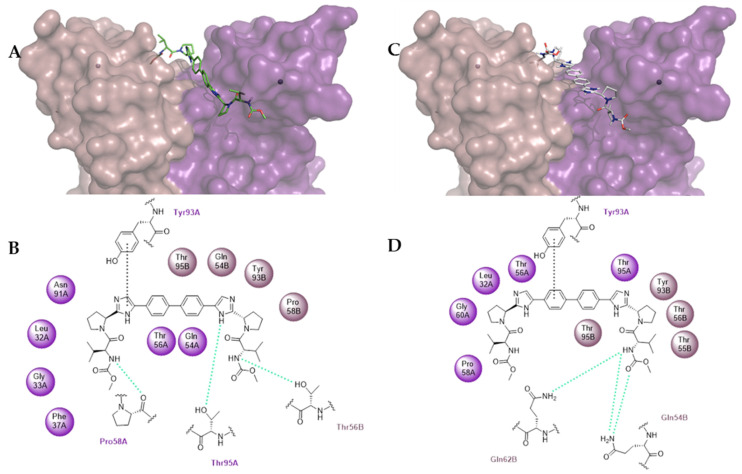
(**A**) Purple and violet surface representation of the HCV GT 1b subunits A and B, respectively, in the presence of **daclatasvir** at binding mode obtained from the OpenEye software as yellow sticks and zinc metal shown as faded balls, (**B**) 2D representation of daclatasvir binding network as obtained from OpenEye software. Residues of purple and violet color are for subunits A and B, respectively. Green, dashed lines represent HB between protein and daclatasvir, while black, dashed lines refer to *π-π* interactions. (**C**) Purple and violet surface representation of the HCV GT 1b subunits A and B, respectively, in the presence of daclatasvir; binding pose predicted by the software PyRx as gray sticks and zinc metal, (**D**) 2D representation of daclatasvir binding network as predicted by the PyRx software. Residues of purple and violet color are for subunits A and B, respectively. Green, dashed lines represent HB between protein and daclatasvir, while black, dashed lines refer to *π-π* interactions.

Compound **10a** exhibits a therapeutic potential similar to the known drug daclatasvir, both having a picomolar EC_50_ value. Its chemical structure incorporates a core connected to *m*, *m’* cap substitution that showed a rich hydrogen bond (HB) network and several hydrophobic and/or *π-π* interactions (Figure 3) with docking scores of −4.04 Chemgauss4 in the OpenEye software and −8.80 kcal/mol in the PyRx software, respectively. The binding mode witnessed in both software programs is balancing between the two Tyr93 A/B residues in either subunit. The imidazole ring forms H-bonds (HBs), and the phenyl ring of the caps is engaged in hydrophobic interactions. On the other hand, daclatasvir introduces a *p*, *p’* cap substitution with similar binding modes and interacting residues (Figure 4). The respective docking score of −5.89 Chemgauss4 in the OpenEye software and −8.70 kcal/mol in the PyRx software. Although the *p*, *p’* substitution is not favored in compound **10b**, this is expected if we consider the difference in core length between **10a** and daclatasvir. The latter shows a distance of 13.8 Å from sp^2^ *N*-imidazole to sp^2^ *N*’-imidazole ring and 14.3 Å for **10a**, accordingly.

**Figure 5:**
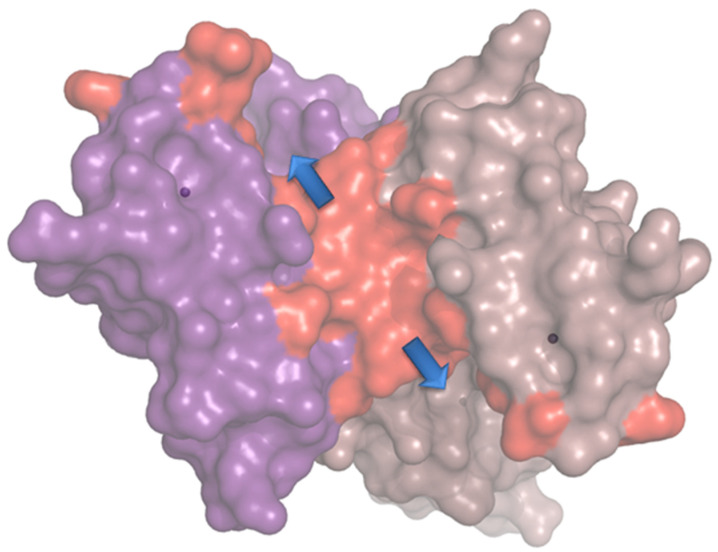
*N*-terminus top view of NS5A dimer protein surface representation with important centered interface formed highlighted in red color. Blue arrows point out symmetric rifts on the side of the protein.

Importantly, as mentioned in the present study and our previous results [27] highly active NS5A inhibitors tend to occupy the centered interface (Figure 5) between the two Tyr93 A/B residues, while solutions traversing down the rifts in either side “due to the protein’s antiparallel symmetry” match with less active compounds (see Supplementary Materials (Figures S1–S21)). Moreover, based on the docking score comparison (see Supplementary Materials Table S1) between two different software programs used in our study, the models represent an almost 90% probability that most active compounds will likely be included in top spots. Thus, models showcase a desired compatibility between in vitro and in silico results, providing further information on possible NS5A inhibitors mode of activity concerning the previously specified region [27,39] of NS5A genotype 1b. Concluding to somewhat higher confidence for future rational drug design efforts.

#### 2.3.3. Pharmacochemical Evaluation and Drug-Likeness

Moreover, with the use of the FAF4 online server [40], pharmacochemical profiling (see Supplementary Materials Table S2a,b) was performed for all new compounds, including the reference compound (daclatasvir). In an attempt to extract valuable information from the data, we see that the number of tolerated rotatable bonds (RB) in DAAs shows a range between 13–18 since greater than 18 witnessed in the case of compounds **4a** and **4b** (i.e., RB = 20) with either *m*, *m*’- or *p*, *p*’-substitution was included in the least active compounds. For the sum of the compounds, we did not find any correlation regarding values on tPSA or HBD/HBA count since all are identical with each other. Focusing on daclatasvir and **10a**, which both exhibited a picomolar antiviral activity on NS5A Gt1b, we see an acceptable range in values on both ionic or not partition coefficients (i.e., logD = 4.16 vs. 6.23/logP = 5.09 vs. 6.01) and flatness (i.e., Fsp^3^ = 0.45 vs. 0.27) for daclatasvir vs. **10a**, respectively.

#### 2.3.4. Activity of Compound 10a in Other HCV Genotypes

As compound **10a** exhibited the highest potency among all synthesized compounds against HCV 1b, it was selected for further characterization. Specifically, we determined the activity of **10a** against viral replication by measuring luciferase activity in Huh7-JFH1, Huh7.5-3a, and Huh7.5-4a stable cell lines containing subgenomic replicons of HCV GT 2a (strain JFH1), GT 3a (strain S52) and GT 4a (strain ED43), respectively. In addition, the activity of **10a** was determined in Huh7-Lunet cells electroporated with the in vitro transcribed full-length reporter viral RNA of HCV genotype 1a (strain H77S.3). Compound **10a** exhibited activity against all four tested genotypes, with the highest activity shown against HCV 2a, with EC_50_ = 28.0 nM (Table 4).

#### 2.3.5. Validation of Compound Activity with Additional Assays

**Figure 6:**
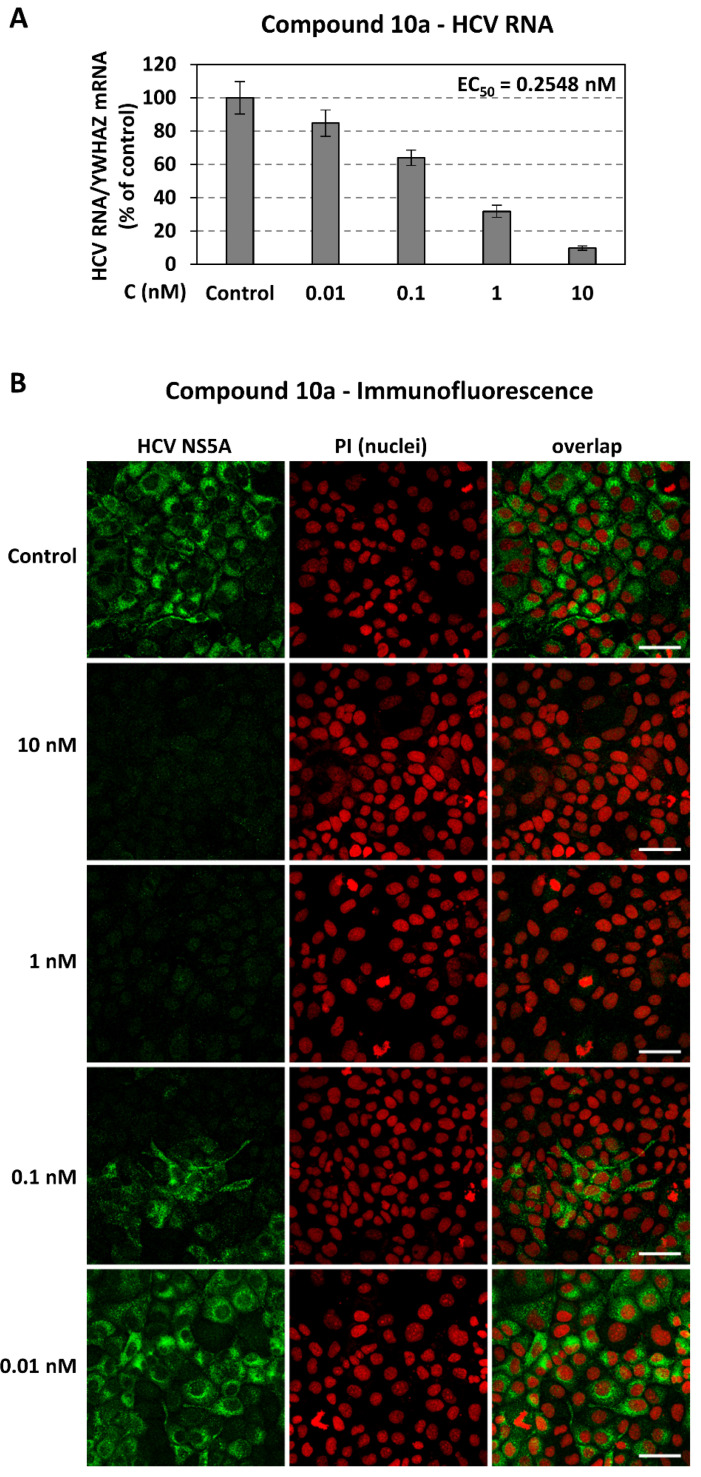
Inhibition of HCV RNA and protein expression levels exerted by compound **10a** in HCV Con1 (GT 1b) subgenomic replicon cell line. (**A**) RT-qPCR analysis of (+) strand HCV RNA levels in Huh5-2 replicon cells treated with 10-fold serial dilutions of compound **10a** or the solvent DMSO. Values are expressed as a percentage of those obtained from cells treated with the solvent (control). The housekeeping gene YWHAZ (tyrosine 3-monooxygenase/tryptophan 5-monooxygenase activation protein zeta) was used as a normalization control. (**B**) Indirect immunofluorescence for NS5A (left column) in Huh5-2 replicon cells treated with 10-fold serial dilutions of compound **10a** or the solvent DMSO. Nuclei were stained with propidium iodide (PI; middle panel). Merged images are shown on the right. Scale bar, 50 μm.

The potency of compound **10a** measured by the luciferase assay in HCV GT 1b was confirmed at the level of viral RNA and NS5A protein expression. Huh5-2 replicon cells were treated with **10a** in serial dilutions, or its solvent DMSO (control), and viral RNA was quantified by reverse transcription-quantitative polymerase chain reaction (RT-qPCR). NS5A protein levels were evaluated by indirect immunofluorescence combined with confocal microscopy analysis. HCV RNA replication was reduced in the presence of the compound with EC_50_ = 0.25 nM (Figure 6A), which is close to the one determined in the luciferase assay (EC_50_ = 0.10 nM). A consistent reduction of NS5A expression was detected, as shown in Figure 6B, in a dose-dependent manner. Nuclei were stained with propidium iodide (PI) as a cell viability control.

#### 2.3.6. Evaluation of Metabolic Stability

Phase I and phase II metabolic stability were measured for our most potent compound **10a** to further evaluate its applicability to go into in vivo studies. This was done using human liver S9 fractions, following the previously reported protocol [41,42]. A number of samples were taken at defined time points, and using LC-MS/MS, the remaining percentage of the parent compound was determined. The calculated half-life time was more than 120 min, indicating high metabolic stability.

## 3. Materials and Methods

### 3.1. Molecular Modeling

A library of the newly synthesized compounds **1a**–**10a**, **1b**–**10b** and daclatasvir serving as control substances were compiled in both a smile formatted file (*.smi) and a Sybyl MOL2 file (*.mol2) using the free program Open Babel v3.1.1 [43]. Following this step, the SMILES library was transformed to SDF format, and their conformers were generated using Omega v.4.1.0.0 software (OpenEye Scientific Software, Inc., Santa Fe, NM, USA; www.eyesopen.com, accessed on 27 February 2021) [44,45]. Experiments were then performed running on a basic laptop PC with an operating system of Windows 10 64-bit (Intel^®^ Core™ i5-1035G1 1.00 GHz CPU processors, RAM 8 GB), using both the OEDocking suite v4.0.0.0 programs (OpenEye Scientific Software, Inc.) [46,47,48] and the PyRx v. 0.8 program (The Scripps Research Institute, La Jolla, CA, USA) [49] for replication reasons. Visualization of the results was performed using the software PyMol v1.4.1 [50].

#### 3.1.1. Ligand and Protein Preparation

The AB dimer crystal structure of HCV NS5A protein genotype 1b (PDB entry 3fqm [37]) was downloaded from the protein databank [51]. The PDB file was cleaned of water molecules and prepared with the OEDocking Suite program MAKE RECEPTOR v4.0.0.0 (OpenEye Scientific Software, Inc.) [52,53] to give the respective input oed extension file to be used in simulation experiments. The search space was centered at the zinc regions of the dimer in each case equally for subunits A and B. This generated an initial box of ~70,000 Å^3^, which after a balanced site-shape creation resulted in an outer docking space of approximately ~27,000 Å^3^ for the protein. Neither residue modifications nor any constraints were implemented on the protein in the docking. The compiled SDF library contained all of the compounds **1a**–**10a**, **1b**–**10b** and daclatasvir. Conformer generation took place with the use of Omega v4.1.0.0 software (OpenEye Scientific Software, Inc.) [44,45] by setting a threshold of 600 structures with the flipping option turned off since all molecules possess specific stereochemistry, and the docking was performed with the OEDocking suite program FRED v4.0.0.0 (OpenEye Scientific Software, Inc.) [46,53]. The model calculations performed are produced by an Exhaustive Search Algorithm. Refinement of results was additionally performed to sort poses with standard options by the OEDocking suite program Scorepose v4.0.0.0 (OpenEye Scientific Software, Inc.) [46,47].

#### 3.1.2. Molecular Docking Replication Experiments

The corresponding compiled Sybyl MOL2 library was subjected to MM2 energy minimization [54] with PerkinElmer Chem3D (Waltham, MA, USA) before in silico reproduction using the virtual screening tool PyRx v0.8 (The Scripps Research Institute) [49] and specifically AutoDock Vina [55,56], which incorporates an alternate algorithm to the calculations (i.e., Genetic Algorithm). Similar to the previously documented conditions mentioned herein, docking was performed in the same region of the protein Hepatitis C virus NS5A genotype 1b. Centered at the zinc regions (i.e., axis X: 23.6268, Y: −9.8409, Z: 9.0372) of the dimer’s subunits A and B. This generated an initial box of >60,000 Å^3^ (i.e., dimensions X: 41.3353, Y: 33.7403, Z: 45.1190). Protein was treated as rigid and the sole parameter adjustment was on “exhaustiveness”, which value was set to 20 instead of the default 8.

### 3.2. Pharmacochemical Profiling

Compounds were compiled on a smile formatted file (*.smi) following the generation of their 3D structure file (*.sdf) all with the use of the program Open Babel v3.1.1 [43]. The later SDF file served as the required input file for the pharmacochemical properties calculations performed over the FAF-Drugs4 server [40]. Selected descriptors were XLOGP3 [57] for the predicted lipophilicity and the server’s built-in filter for drug-likeness [58,59,60,61], which takes into account common drug-design properties following Lipinski’s rule of five and others providing predicted calculations for polar surface area (tPSA), flatness (Fsp^3^) hydrogen bond acceptor/donor count, etc. (see Supplementary Materials Table S2a,b). Moreover, compound **3** was positively checked for the detection of undesirable moieties [62], as well as PAINS [63].

### 3.3. Chemistry

Solvents and reagents were obtained from commercial suppliers and were used without further purification. ^1^H-NMR spectra were recorded at 400 MHz and ^13^C-NMR spectra were run at 101 MHz in deuterated chloroform (CDCl_3_). The chemical shifts are referenced in the residual protonated solvent signals. The purities of the tested compounds were determined by HPLC, coupled with mass spectrometry. Mass spectrometric analysis (UPLC-ESI-MS) was performed using a Waters ACQUITY Xevo TQD system, which consisted of an ACQUITY UHPLC H-Class system and Xevo™ TQD triple-quadrupole tandem mass spectrometer with an electrospray ionization (ESI) interface (Waters Corp., Milford, MA, USA). An Acquity BEH C18 100 mm × 2.1 mm column (particle size, 1.7 µm) was used to separate analytes (Waters, Dublin, Ireland). The solvent system consisted of water containing 0.1% formic acid (A) and 0.1% formic acid in acetonitrile (B). HPLC method: flow rate 200 μL/min. The percentage of B started at an initial of 5% and maintained for 1 min, then increased up to 100% during 10 min, kept at 100% for 2 min, and flushed back to 5% in 3 min, then kept at 5% for 1 min. The MS scan was carried out under the following conditions: capillary voltage 3.5 kV, cone voltage 20 V, radio frequency (RF) lens voltage 2.5 V, source temperature 150 °C, and desolvation gas temperature 500 °C. Nitrogen was used as the desolvation and cone gas at flow rates of 1000 and 20 L/h, respectively. System operation and data acquisition were controlled using MassLynx 4.1 software (Waters). HRMS was measured as previously described [64].

#### 3.3.1. General Synthetic Methods and Experimental Details for All Compounds

##### General Procedure for Carbamate Synthesis

In a 250 mL round bottom flask, 1 M NaOH (75 mL) was added and left to cool to 0 °C in an ice bath. After that, the respective amino acid (24 mmol) was added, and the solution was left to stir until it was homogeneous. Then, the respective chloroformate (33 mmol) and 1,4-dioxane (30 mL) were added drop by drop. The reaction mixture was then allowed to stir overnight at room temperature. The solution was extracted with Et_2_O (3 × 50 mL). The aqueous layer was cooled to 0 °C in an ice bath and concentrated HCl was added dropwise until pH = 2. The aqueous solution was extracted again with Et_2_O (3 × 100 mL). The organic layers were combined, dried over anhydrous Na_2_SO_4_, filtered, and concentrated in vacuo to give a viscous oily product. The compound was used for the next step without further purification.

(Methoxycarbonyl)-*L*-valine (***1C***). The compound was synthesized according to the procedure for carbamate synthesis using *L*-valine amino acid and methyl chloroformate to give a white crystalline product: yield 2.5 g (44%); mp 109–113 °C; CAS no. 74761-42-5; C_7_H_13_NO_4_.

(Methoxycarbonyl)-*D*-valine (***2C***). The compound was synthesized according to the procedure for carbamate synthesis using *L*-valine amino acid and methyl chloroformate to give a white crystalline product: yield 2.4 g (57%); mp 109–113 °C; CAS no. 153575-98-5; C_7_H_13_NO_4_.

(Ethoxycarbonyl)-*L*-valine (***3C***). The compound was synthesized according to the procedure for carbamate synthesis using *L*-valine amino acid and ethyl chloroformate to give a clear oily product: yield 2.4 g (52%); CAS no. 5701-14-4; C_8_H_15_NO_4_.

(Butoxycarbonyl)-*L*-valine (***4C***). The compound was synthesized according to the procedure for carbamate synthesis using *L*-valine amino acid and butyl chloroformate to give a clear oily product: yield 3.1 g (60%); CAS no. 122315-77-9; C_10_H_19_NO_4_.

(Methoxycarbonyl)-*L*-leucine (***5C***). The compound was synthesized according to the procedure for carbamate synthesis using *L*-leucine amino acid and methyl chloroformate to give a clear oily product: yield 2.6 g (58%); CAS no. 74761-37-8; C_8_H_15_NO_4_.

(Methoxycarbonyl)-*D*-leucine (***6C***). The compound was synthesized according to the procedure for carbamate synthesis using *D*-leucine amino acid and methyl chloroformate to give a clear oily product: yield 2.4 g (52%); CAS no. 791635-26-2; C_8_H_15_NO_4_.

(Ethoxycarbonyl)-*L*-leucine (***7C***). The compound was synthesized according to the procedure for carbamate synthesis using *L*-leucine amino acid and ethyl chloroformate to give a clear oily product: yield 2.5 g (51%); CAS no. 19887-30-0; C_9_H_17_NO_4_.

(Ethoxycarbonyl)-*D*-leucine (***8C***). The compound was synthesized according to the procedure for carbamate synthesis using *D*-leucine amino acid and ethyl chloroformate to give a clear oily product: yield 2.7 g (55%); CAS no. 136159-70-1; C_9_H_17_NO_4_.

(Methoxycarbonyl)-*L*-phenylglycine (***9C***). The compound was synthesized according to the procedure for carbamate synthesis using *L*-phenylglycine amino acid and methyl chloroformate to give a clear oily product: yield 2.8 g (58%); CAS no. 60725-19-1; C_10_H_11_NO_4_.

(Methoxycarbonyl)-*D*-phenylglycine (***10C***). The compound was synthesized according to the procedure for carbamate synthesis using *D*-phenylglycine amino acid and methyl chloroformate to give a clear oily product: yield 2.6 g (54%); C_10_H_11_NO_4_.

###### General Procedure for Alpha Carbon Bromination

*N*-bromosuccinimide (6.25 mmol, 1.11 g) was added to the appropriate iodoacetophenone (5 mmol, 1.22 g) in acetonitrile (30 mL), the mixture was stirred at room temperature for 10–15 min, then p-toluene sulfonic acid (*p*-TsOH) (10 mmol, 1.90 g) was added to the mixture and refluxed for 2 h. The reaction mixture was concentrated under reduced pressure, washed with a saturated solution of Na_2_CO_3_ and extracted with EtOAc. The organic layer was separated and dried over MgSO_4_, filtered then concentrated under reduced pressure.

**2-Bromo-1-(3-iodophenyl) ethan-1-one (A_1_).** Synthesized according to the method outlined above using 3′-iodoacetophenone; orange oil; yield: 1.5 g (92%); ^1^H NMR (400 MHz, CDCl_3_) δ 4.29 (2H, s), 7.32 (1H, ddd*, J* = 7.8, 1.6, 1.2 Hz), 7.52 (1H, ddd, *J* = 7.8, 7.7, 0.4 Hz), 7.94 (1H, ddd, *J* = 7.7, 1.9, 1.2 Hz), 8.12 (1H, ddd, *J* = 1.9, 1.6, 0.4 Hz); MS (ESI): *m*/*z* = 324.8 (M+H^+^).

**2-Bromo-1-(4-iodophenyl) ethan-1-one (A_2_).** Synthesized according to the method outlined above using 4′-iodoacetophenone; orange solid; yield: 1.53 g (94%); mp: 113–115 °C, ^1^H NMR (400 MHz, CDCl_3_) δ 4.28 (2H, s), 7.65 (2H, ddd, *J* = 8.6, 1.4, 0.5 Hz), 7.93 (2H, ddd, *J* = 8.6, 1.8, 0.5 Hz); MS (ESI): *m*/*z* = 324.8 (M+H^+^).

###### General Procedure for Pyrrolidine Dicarboxylate Formation. Boc-L-proline (4.6 mmol, 1.5 g) was added to Compound A_1_ or A_2_ in Acetonitrile (15 mL)

Following that, TEA (25 mmol, 3.50 mL) was added, and the reaction mixture was left to stir at room temperature for 3 h. Then, it was concentrated under vacuum, washed with distilled water, and extracted with EtOAc (50 mL × 3). The combined organic layers were separated and dried over MgSO_4_ and filtered then concentrated under vacuum. The compound was confirmed by MS analysis and used in the next step without further purification.

**1-(*Tert*-butyl) 2-(2-(3-iodophenyl)-2-oxoethyl) pyrrolidine-1,2-dicarboxylate (B_1_).** It was synthesized by reacting 2-bromo-1-(3-iodophenyl) ethan-1-one (**A_1_**) with Boc-L-proline according to the previous general procedure; reddish-brown oil; yield: 1.9 g (90%); ^1^H NMR (400 MHz, CDCl_3_) δ 8.01 (ddd, *J* = 8.0, 1.9, 1.2 Hz, 1H), 7.75 (ddd, *J* = 1.9, 1.6, 0.4 Hz, 1H), 7.56 (ddd, *J* = 8.0, 7.8, 0.4 Hz, 1H), 7.30 (ddd, *J* = 7.8, 1.6, 1.2 Hz, 1H), 4.54–4.58 (m, 2H), 4.33 (dd, *J* = 9.3, 5.6 Hz, 1H), 3.20–3.52 (m, 2H), 1.77–2.23 (m, 4H), 1.42 (s, 9H); MS (ESI): *m*/*z* = 460.0 (M+H^+^).

**1-(*Tert*-butyl) 2-(2-(4-iodophenyl)-2-oxoethyl) pyrrolidine-1, 2-dicarboxylate (B_2_).** It was synthesized by reacting 2-bromo-1-(4-iodophenyl) ethan-1-one (**A_2_**) with Boc-L-proline according to the previous general procedure; reddish brown semi solid; yield: 2.0 g (95%); ^1^H NMR (400 MHz, CDCl_3_) δ 7.94 (ddd, *J* = 8.6, 1.8, 0.4 Hz, 2H), 7.65 (ddd, *J* = 8.6, 1.4, 0.4 Hz, 2H), 4.60 (s, 2H), 4.24 (dd, *J* = 9.3, 5.6 Hz, 1H), 3.35 (ddd, *J* = 15.6, 6.9, 1.8 Hz, 2H), 1.77–2.23 (m, 4H), 1.42 (s, 9H); MS (ESI): *m*/*z* = 460.0 (M+H^+^)

###### General Procedure for the Formation of the Imidazole Ring

In a one-pot, multistep transformation, compound B_1_ or B_2_ was exposed to ammonium acetate (75 mmol, 5.78 g) in toluene (20 mL) under reflux. The reaction mixture was concentrated under vacuum, washed with distilled water, and extracted with EtOAc (50 × 3). The combined organic layers were separated and dried over MgSO_4_, filtered then concentrated under vacuum. The product was purified with silica gel column chromatography.

***Tert*-butyl (*S*)-2-(5-(3-iodophenyl)-1*H*-imidazol-2-yl) pyrrolidine-1-carboxylate (C_1_).** It was synthesized by reacting 1-(*tert*-butyl) 2-(2-(3-iodophenyl)-2-oxoethyl) pyrrolidine-1,2-dicarboxylate (**B_1_**) with ammonium acetate, as mentioned in the general procedure for imidazole formation. The product was purified using hexane/EtOAc 2:1 with 1% TEA; brown solid; yield: 1.2 g (70%); mp: 161–164 °C; ^1^H NMR (400 MHz, CDCl_3_) δ 7.84 (ddd, *J* = 8.2, 7.7, 0.5 Hz, 1H), 7.56 (ddd, *J* = 1.7, 1.5, 0.5 Hz, 1H), 7.51 (s, 1H), 7.30–7.39 (m, 2H), 5.38 (dd, *J* = 7.4, 7.1 Hz, 1H), 3.38–3.59 (m, 2H), 1.73–2.33 (m, 4H), 1.35 (s, 9H); MS (ESI): *m*/*z* = 440.0 (M+H)^+^.

***Tert*-butyl (*S*)-2-(5-(4-iodophenyl)-1*H*-imidazol-2-yl) pyrrolidine-1-carboxylate (C_2_).** It was synthesized by reacting 1-(*Tert*-butyl) 2-(2-(4-iodophenyl)-2-oxoethyl) pyrrolidine-1, 2-dicarboxylate (**B_2_**) with ammonium acetate as in the general procedure for imidazole formation; the product was purified using hexane: EtOAc 2:1 with 1% TEA; buff solid; yield: 1.1 g (69%); mp: 156–158 °C; ^1^H NMR (400 MHz, CDCl_3_) 7.93 (ddd, *J* = 8.6, 1.7, 0.5 Hz, 2H), 7.62-7.68 (m, 3H), 5.29 (dd, *J* = 7.4, 7.1 Hz, 1H), 3.51 (m, 2H), 2.25 (m, 1H),1.82-2.15 (m, 3H), 1.41 (s, 9H); MS (ESI): *m*/*z* = 440.0 (M+H)^+^.

###### General Procedure for Boc Deprotection

Trifluoroacetic acid (2 mL) was added to compound C_1_ or C_2_ in dichloromethane (15 mL) at room temperature, and the reaction was left for 3 h. The reaction mixture was concentrated under reduced pressure, neutralized with 5 M NaOH, and extracted with EtOAc. The organic layer was separated, dried over MgSO_4_, and filtered then concentrated under reduced pressure.

**(*S*)-5-(3-Iodophenyl)-2-(pyrrolidin-2-yl)-1*H*-imidazole (D_1_).** It was deprotected as mentioned in the general procedure above; yellow semisolid; yield: 0.8 g (88%); ^1^H NMR (400 MHz, CDCl_3_) δ 7.81 (ddd, *J* = 8.2, 7.7, 0.5 Hz, 1H), 7.70 (ddd, *J* = 1.7, 1.5, 0.5 Hz, 1H), 7.28–7.64 (m, 3H), 4.61 (dd, *J* = 7.7, 6.9 Hz, 1H), 3.22 (ddd, *J* = 6.9, 3.2, 1.7 Hz, 1H), 3.01 (ddd, *J* = 7.7, 6.9, 3.2 Hz, 1H), 1.43–2.12 (m, 4H); MS (ESI): *m*/*z* = 340.0 (M+H)^+^.

**(*S*)-5-(4-Iodophenyl)-2-(pyrrolidin-2-yl)-1*H*-imidazole (D2).** It was deprotected as mentioned in the general procedure above; light brown semisolid; 0.8 g (88%); ^1^H-NMR (400 MHz, CDCl_3_) δ 7.93 (ddd*, J* = 8.6, 1.7, 0.5 Hz, 2H), 7.61-7.68 (m, 2H), 7.60 (s, 1H), 4.33 (dd, *J* = 7.7, 6.9 Hz, 1H), 3.15 (ddd, *J* = 6.9, 3.2, 1.7 Hz, 1H), 2.91 (ddd*, J* = 7.7, 6.9, 3.2 Hz, 1H), 1.98–2.19 (m, 2H), 1.62–1.95 (m, 2H); MS (ESI) *m*/*z* = 340.0 (M+H)^+^.

###### General Procedure for Amide Coupling, Compounds **1**–**20**

To a suspension of compound **D_1_** or **D_2_** (2.35 mmol, 0.8 g), the corresponding carbamate (3 equiv.) and TEA (2 mL) in DCM (50 mL), HBTU (8.82 mmol, 3.34 g) were added, and the mixture was stirred at room temperature for 2 h. The reaction mixture was concentrated under vacuum, washed with distilled water, and extracted with DCM (50 × 3). The combined organic layers were dried over MgSO_4_, filtered and concentrated under vacuum. The product was purified using silica gel column chromatography.

**Methyl ((*S*)-1-((*S*)-2-(5-(3-iodophenyl)-1*H*-imidazol-2-yl)pyrrolidin-1-yl)-3-methyl-1-oxobutan-2-yl)carbamate (1).** It was prepared by coupling compound (**D_1_**) with methyl-*L*-valine carbamate using the previous general procedure, and it was purified using DCM: MeOH with a ratio 20:1; yellow semisolid; 1.0 g (77%); ^1^H-NMR (400 MHz, CDCl_3_) δ 8.07 (d, *J* = 37.2 Hz, 1H), 7.71–7.50 (m, 2H), 7.18–7.01 (m, 2H), 5.62 (t, *J* = 9.9 Hz, 1H), 5.21 (dt, *J* = 8.9, 4.5 Hz, 1H), 4.23 (ddd, *J* = 35.0, 11.9, 7.2 Hz, 1H), 3.94–3.70 (m, 2H), 3.60 (s, 3H), 2.37–1.92 (m, 5H), 0.98–0.82 (m, 6H); ^13^C-NMR (101 MHz, CDCl_3_) δ 172.64, 165.73, 157.10, 148.38, 140.22, 135.44, 133.53, 130.24, 127.41, 123.76, 94.73, 57.65, 54.35, 52.36, 47.89, 31.16, 27.46, 25.36, 17.58; MS (ESI) *m/z =* 497.0 [M+H]^+^.

**Methyl ((*S*)-1-((*S*)-2-(5-(4-iodophenyl)-1*H*-imidazol-2-yl)pyrrolidin-1-yl)-3-methyl-1-oxobutan-2-yl) carbamate (2)**. It was prepared by coupling compound (**D_2_**) with methyl-*L*-valine carbamate using the previous general procedure, and it was purified using DCM: MeOH 100:3; light brown semisolid; yield: 0.7 g (53%); ^1^H NMR (400 MHz, CDCl_3_) δ 7.64 (d, *J* = 8.4 Hz, 2H), 7.52–7.34 (m, 2H), 7.18 (s, 1H), 5.52 (d, *J* = 9.1 Hz, 1H), 5.22 (dd, *J* = 8.1, 3.7 Hz, 1H), 4.31 (dd, *J* = 9.0, 6.8Hz, 1H), 3.67 (s, 3H), 3.65–3.43 (m, 2H), 2.22–1.90 (m, 5H), 0.86 (dd, *J* = 6.6, 3.6 Hz, 6H); ^13^C NMR (101 MHz, CDCl_3_) δ 172.53, 157.22, 148.62, 136.66, 135.34, 132.87, 124.65, 119.56, 118.72, 82.35, 74.31, 58.29, 57.83, 52.47, 48.03, 31.32, 25.35, 19.32, 17.77; MS (ESI) *m/z =* 497.0 [M+H]^+^.

**Methyl ((*R*)-1-((*S*)-2-(5-(3-iodophenyl)-1*H*-imidazol-2-yl)pyrrolidin-1-yl)-3-methyl-1-oxobutan-2-yl)carbamate (3).** It was prepared by coupling compound (**D_1_**) with methyl-*D*-valine carbamate using the previous general procedure, and it was purified using DCM: MeOH 20:1; yellow semisolid; yield: 0.8 g (61%); ^1^H NMR (400 MHz, CDCl_3_) δ 7.99 (dd, *J* = 8.3, 1.7 Hz, 1H), 7.56 (dd*, J* = 10.3, 3.4 Hz, 2H), 7.13–7.04 (m, 2H), 5.71 (d*, J* = 6.9 Hz, 1H), 5.35 (d, *J* = 8.5 Hz, 1H), 4.30–4.27 (m, 1H), 3.67 (s, 3H), 3.62 (s, 2H), 2.23–2.04 (m, 5H), 1.03–0.99 (m, 6H); ^13^C NMR (101 MHz, CDCl_3_) δ 174.77, 172.27, 165.86, 158.06, 157.05, 148.54, 136.20, 133.75, 130.34, 124.32, 94.62, 58.95, 58.73, 52.79, 47.52, 31.13, 30.46, 24.42, 19.05; MS (ESI) *m*/*z* = 497.0 [M+H]^+^.

**Methyl ((*R*)-1-((*S*)-2-(5-(4-iodophenyl)-1*H*-imidazol-2-yl)pyrrolidin-1-yl)-3-methyl-1 oxobutan-2-yl)carbamate (4).** It was prepared by coupling compound (**D_2_**) with methyl-*D*-valine carbamate using the previous general procedure, and it was purified using DCM: MeOH 100:5; yellow semisolid; yield: 0.6 g (46%); ^1^H NMR (400 MHz, CDCl_3_) δ 7.66–7.60 (m, 2H), 7.42 (d, *J* = 8.2 Hz, 2H), 7.18 (d, *J* = 4.7 Hz, 1H), 5.47 (d, *J* = 7.8 Hz, 1H), 5.28 (d, *J* = 6.3 Hz, 1H), 4.20 (t, *J* = 7.6 Hz, 1H), 3.89 (t, *J* = 7.9 Hz, 1H), 3.63 (s, 3H), 3.59 (dd, *J* = 13.1, 5.7 Hz, 2H), 2.10 (dd, *J* = 20.6, 13.7, 11.5 Hz, 4H), 1.02 (dd, *J* = 6.6, 3.3 Hz, 6H); ^13^C NMR (101 MHz, CDCl_3_) δ 172.30, 157.59, 148.48, 147.32, 137.69, 130.56, 126.68, 117.96, 91.65, 58.47, 55.25, 52.64, 47.69, 30.93, 29.35, 24.81, 18.20; MS (ESI) *m/z =* 497.0 [M+H]^+^.

**Ethyl ((*S*)-1-((*S*)-2-(5-(3-iodophenyl)-1*H*-imidazol-2-yl)pyrrolidin-1-yl)-3-methyl-1-oxobutan-2-yl)carbamate (5).** It was prepared by coupling compound (**D_1_**) with ethyl-*L*-valine carbamate using the previous general procedure, and it was purified using DCM: MeOH 20:1; yellow semisolid; yield: 0.6 g (50%); ^1^H NMR (400 MHz, CDCl_3_) δ 7.97 (s, 1H), 7.57–7.50 (m, 2H), 7.20–7.01 (m, 2H), 5.61 (t, *J* = 47.2 Hz, 1H), 5.22 (dd, *J* = 8.1,4.0 Hz, 1H), 4.32–4.27 (m, 1H), 4.10 (dd, *J* = 8.0, 3.9 Hz, 2H), 3.87–3.63 (m, 2H), 2.35–1.94 (m, 5H), 1.07–0.97 (m, 3H), 0.91–0.84 (m, 6H); ^13^C NMR (101 MHz, CDCl_3_) δ 172.59, 165.59, 156.54, 148.19, 135.38, 133.32, 130.08, 123.88, 123.56, 118.80, 94.53, 61.02, 57.39, 54.18, 47.71, 30.86, 25.14, 18.91, 17.41, 14.36; MS (ESI) *m/z =* 511 [M+H]^+^.

**Ethyl ((*S*)-1-((*S*)-2-(5-(4-iodophenyl)-1*H*-imidazol-2-yl) pyrrolidin-1-yl)-3-methyl-1-oxobutan-2-yl) carbamate (6).** It was prepared by coupling compound (**D_2_**) with ethyl-*L*-valine carbamate using the previous general procedure, and it was purified using DCM: MeOH 100:3; yellow semisolid; yield: 0.9 g (75%); ^1^H NMR (400 MHz, CDCl_3_) δ 7.77–7.29 (m, 4H), 7.18 (d, *J* = 22.4 Hz, 1H), 5.46 (d, *J* = 9.0 Hz, 1H), 5.23 (dd, *J* = 7.7, 3.7 Hz, 1H), 4.20 (ddd, *J* = 11.1,10.0,5.7 Hz, 3H), 3.81 (dd, *J* = 38.3, 31.6 Hz, 2H), 2.36–1.94 (m, 5H), 1.25 (t, *J* = 7.0 Hz, 3H), 0.91 (dd, *J* = 27.1, 16.0 Hz, 6H); ^13^C NMR (101 MHz, CDCl_3_) δ 172.93, 156.84, 148.55, 143.57, 137.76, 126.70, 126.54, 118.65, 96.46, 61.35, 57.63, 54.52, 48.05, 31.26, 27.54, 25.50, 19.40, 14.69; MS (ESI) *m/z =* 511.0 [M+H]^+^.

**Butyl ((*S*)-1-((*S*)-2-(5-(3-iodophenyl)-1*H*-imidazol-2-yl)pyrrolidin-1-yl)-3-methyl-1-oxobutan-2-yl)carbamate (7).** It was prepared by coupling compound (**D_1_**) with butyl-*L*-valine carbamate using the previous general procedure, and it was purified using DCM: MeOH 25:1; yellow semisolid; yield: 1.0 g (78%); ^1^H NMR (400 MHz, CDCl_3_) δ 8.00–7.80 (m, 1H), 7.57 (ddd, *J* =26.3, 23.6, 7.9 Hz, 2H), 7.36 (ddd, *J* = 33.7, 17.4, 6.9 Hz, 1H), 7.06 (dd, *J* = 16.0, 8.0 Hz, 1H), 5.90–5.33 (m, 1H),5.24 (d, *J* = 4.4 Hz, 1H), 4.31–4.28 (m, 1H), 4.05 (d, *J* = 6.6 Hz, 3H), 3.93–3.48 (m, 1H), 2.40–1.89 (m, 4H), 1.57 (d, *J* = 6.4 Hz, 3H), 1.36 (s, 2H), 0.91 (d, *J* = 6.4 Hz, 9H); ^13^C NMR (101 MHz, CDCl_3_) δ 175.52, 156.63, 154.49, 148.03, 135.87, 134.97, 133.44, 130.19, 128.59, 123.71, 94.54, 64.99, 58.65, 54.17, 53.25, 47.82, 38.47, 30.92, 25.15, 18.85, 17.22, 13.55; MS (ESI) *m/z =* 539.0 [M+H]^+^.

**Butyl ((*S*)-1-((*S*)-2-(5-(4-iodophenyl)-1*H*-imidazol-2-yl) pyrrolidin-1-yl)-3-methyl-1-oxobutan-2-yl) carbamate (8).** It was prepared by coupling compound (**D_2_**) with butyl-*L*-valine carbamate using the previous general procedure, and it was purified using EtOAc: hexane 2:1; yellow semisolid; yield: 0.9 g (71%), ^1^H NMR (400 MHz, CDCl_3_) δ 7.64 (t, *J* = 8.3 Hz, 2H), 7.55–7.27 (m, 2H), 7.18 (s, 1H), 5.44 (d*, J* = 9.1 Hz, 1H), 5.23 (dd, *J* = 8.1, 3.9 Hz, 1H), 4.35–4.12 (m, 1H),4.06 (td, *J* = 10.8, 3.9 Hz, 2H), 3.89–3.60 (m, 2H), 2.44–1.86 (m, 4H), 1.59 (dd, *J* = 14.2, 6.4 Hz, 2H), 1.37 (dd, *J* = 15.0, 7.4 Hz, 2H), 1.25 (dd, *J* = 8.1, 6.2 Hz, 1H), 0.95–0.83 (m, 9H); ^13^C NMR (101 MHz, CDCl_3_) δ 172.97, 156.98, 148.52, 143.24, 137.77, 126.77, 126.52, 115.63, 65.31, 57.69, 54.55, 48.07, 31.25, 31.14, 27.67, 25.48, 19.18, 17.63, 13.88; MS (ESI) *m/z =* 539.0 [M+H]^+^.

**Methyl ((*S*)-1-((*S*)-2-(5-(3-iodophenyl)-1*H*-imidazol-2-yl)pyrrolidin- 1-yl)-4-methyl-1-oxopentan-2-yl)carbamate (9).** It was prepared by coupling compound (**D_1_**) with methyl-*L*-leucine carbamate using the previous general procedure, and it was purified using DCM: MeOH 20:1; yield: 0.7 g (58%), yellow semisolid; ^1^H NMR (400 MHz, CDCl_3_) δ 8.13–7.95 (m, 1H), 7.54 (dt, *J* = 8.2,4.1 Hz, 2H), 7.22–7.02 (m, 2H), 5.48 (d, *J* = 8.8 Hz, 1H),5.22 (dd, *J* = 8.0, 3.4 Hz, 1H), 4.53 (td, *J* = 9.8, 3.8 Hz, 1H),3.67 (s, 3H), 3.65–3.47 (m, 2H), 1.87–1.54 (m, 4H), 1.53– 1.37 (m, 2H), 1.36–1.23 (m, 1H), 0.94 (dd, *J* = 5.6, 3.2Hz, 6H); ^13^C NMR (101 MHz, CDCl_3_) δ 173.38, 165.63, 156.79, 156.58, 148.06, 146.87, 135.52, 133.34, 130.11, 123.64, 94.55, 54.30, 52.19, 50.90, 47.35, 41.37, 27.68, 25.16, 24.44, 23.14; MS (ESI) *m/z =* 511.0 [M+H]^+^.

**Methyl ((*S*)-1-((*S*)-2-(5-(4-iodophenyl)-1*H*-imidazol-2-yl) pyrrolidin-1-yl)-4-methyl-1-oxopentan-2-yl) carbamate (10).** It was prepared by coupling compound **(D_2_)** with methyl-*L*-leucine carbamate using the previous general procedure, and it was purified using DCM: MeOH 100:3; yellow semisolid; yield: 0.8 g (66%); ^1^H NMR (400 MHz, CDCl_3_) δ 7.65 (t, *J* = 8.9 Hz, 2H), 7.40 (dt, *J* = 14.0, 7.0 Hz, 2H), 7.18 (s, 1H), 5.46 (d, *J* = 8.7 Hz, 1H), 5.22 (dd, *J* = 7.9, 2.8 Hz, 1H), 4.53 (td, *J* = 9.9, 3.6 Hz, 1H), 3.69 (s, 3H), 3.63–3.46 (m, 2H), 2.61 (ddd, *J* = 18.3, 10.4, 3.1 Hz, 2H), 2.13–1.02 (m, 5H),0.98–0.87 (m, 6H); ^13^C NMR (101 MHz, CDCl_3_) δ 173.71, 157.08, 151.73, 148.36, 137.79, 126.76, 126.57, 111.35, 91.92, 54.64, 52.51, 51.20, 47.66, 41.79, 27.84, 24.74, 23.45, 21.65; MS (ESI) *m/z =* 511.0 [M+H]^+^.

**Methyl ((*R*)-1-((*S*)-2-(5-(3-iodophenyl)-1*H*-imidazol-2-yl)pyrrolidin- 1-yl)-4-methyl-1-oxopentan-2-yl)carbamate (11).** It was prepared by coupling compound (**D_1_**) with methyl-*D*-leucine carbamate using the previous general procedure, and it was purified using DCM: MeOH 20:1; yellow semisolid; yield: 0.8 g (66%); ^1^H NMR (δ) (400 MHz, CDCl_3_) δ 8.17–7.95 (m, 1H), 7.69–7.56 (m, 1H), 7.52 (d, *J* = 7.9 Hz, 1H), 7.17–7.03 (m, 2H), 5.47 (dd, *J* = 26.5, 8.6 Hz, 1H), 5.22 (dd, *J* = 8.0, 2.9 Hz, 1H), 4.54 (td, *J* = 9.8, 3.8 Hz, 1H), 3.68 (s, 3H), 3.55 (ddd, *J* = 24.6,11.7, 5.8 Hz, 2H), 2.43–2.06 (m, 4H), 1.75 (ddd, *J* = 20.4,13.7, 10.1 Hz, 2H), 1.39–1.31 (m, 1H), 0.94–0.87 (m, 6H); ^13^C NMR (101 MHz, CDCl_3_) δ 173.44, 156.92, 155.65, 148.31, 139.30, 135.49, 133.52, 130.25, 123.79, 115.96, 94.75, 54.47, 52.34, 51.00, 47.50, 41.80, 27.50, 25.38, 24.60, 23.34, 21.55; MS (ESI) *m/z=* 511.0 [M+H]^+^.

**Methyl ((*R*)-1-((*S*)-2-(5-(4-iodophenyl)-1*H*-imidazol-2-yl) pyrrolidin-1-yl)-4-methyl-1-oxopentan-2-yl) carbamate (12).** It was prepared by coupling compound (**D_2_**) with methyl-*D*-leucine carbamate using the previous general procedure, and it was purified using DCM: MeOH 100:3; yellow semisolid; yield: 0.55 g (45%); ^1^H NMR (400 MHz, CDCl_3_) δ 7.65 (t, *J* = 8.9 Hz, 2H), 7.40 (dt, *J* = 14.0, 7.0 Hz, 2H), 7.18 (s, 1H), 5.46 (d, *J* = 8.7 Hz, 1H), 5.22 (dd, *J* = 7.9, 2.8 Hz, 1H), 4.53 (td, *J* = 9.9, 3.6 Hz, 1H), 3.69 (s, 3H), 3.63–3.46 (m, 2H), 2.61 (ddd, *J* = 18.3, 10.4, 3.1 Hz, 2H), 2.13–1.02 (m, 5H),0.98–0.87 (m, 6H); ^13^C NMR (101 MHz, CDCl_3_) δ 173.71, 157.08, 151.73, 148.36, 137.79, 126.76, 126.57, 111.35, 91.92, 54.64, 52.51, 51.20, 47.66, 41.79, 27.84, 24.74, 23.45, 21.65; MS (ESI) *m/z =* 511.0 [M+H]^+^.

**Ethyl ((*S*)-1-((*S*)-2-(5-(3-iodophenyl)-1*H*-imidazol-2-yl)pyrrolidin-1-yl)-4-methyl-1oxopentan-2-yl)carbamate (13).** It was prepared by coupling compound (**D_1_**) with ethyl-*L*-leucine carbamate using the previous general procedure, and it was purified using DCM: MeOH 20:1; yellow semisolid; yield: 0.75 g (62%); ^1^H NMR (400 MHz, CDCl_3_) δ 7.61 (d, *J* = 7.8 Hz, 1H), 7.45 (d, *J* = 7.7 Hz, 1H), 7.28 (d, *J* = 6.0 Hz, 1H), 7.08 (dt, *J* = 15.8, 8.0 Hz, 2H), 5.24 (dd, *J* = 13.0, 7.5 Hz, 1H), 5.13 (s, 1H), 4.54–4.46 (m, 1H), 4.10 (s, 2H), 3.91–3.79 (m, 2H), 2.50–2.11 (m, 4H), 1.73–1.70 (m, 2H), 1.54 (s, 1H), 1.23 (s, 3H), 0.95 (s, 6H); ^13^C NMR (101 MHz, CDCl_3_) δ 173.79, 156.52, 156.27, 148.10, 145.99, 141.50, 137.03, 133.40, 130.45, 123.95, 94.56, 61.03, 52.15, 51.34, 47.43, 41.47, 24.60, 22.74, 21.57, 21.09, 14.34; MS (ESI) *m/z =* 525.0 [M+H]^+^.

**Ethyl ((*S*)-1-((*S*)-2-(5-(4-iodophenyl)-1*H*-imidazol-2-yl)pyrrolidin-1-yl)-4-methyl-1-oxopentan-2-yl)carbamate (14).** It was prepared by coupling compound (**D_2_**) with ethyl-*L*-leucine carbamate using the previous general procedure, and it was purified using DCM: MeOH 100: 4.5; dark yellow oil; yield: 0.65 g (52%); ^1^H NMR (δ) (400 MHz, CDCl_3_) δ 7.79–7.29 (m, 4H), 7.19 (s, 1H), 5.59–5.31 (m, 1H), 5.30–5.05 (m, 1H), 4.21 (dd, *J* = 91.9, 85.2 Hz, 3H), 3.87–3.53 (m, 2H), 1.92 (ddd, *J* = 261.2, 54.6, 6.4 Hz, 7H), 1.24 (d, *J* = 7.1 Hz, 3H), 0.97–0.89 (m, 6H); ^13^C NMR (101 MHz, CDCl_3_) δ 177.25, 156.64, 155.31, 143.30, 138.04, 128.61, 126.80, 119.15, 102.58, 61.40, 52.50, 51.37, 47.74, 41.68, 24.91, 23.36, 23.03, 21.84, 14.64; MS (ESI) *m/z =* 525.0 [M+H]^+^.

**Ethyl ((*R*)-1-((*S*)-2-(5-(3-iodophenyl)-1*H*-imidazol-2-yl)pyrrolidin-1-yl)-4-methyl-1-oxopentan-2-yl)carbamate (15).** It was prepared by coupling compound (**D_1_**) with ethyl-*D*-leucine carbamate using the previous general procedure, and it was purified using DCM: MeOH; 20:1; yellow semisolid; yield: 0.76 g (60%); ^1^H NMR (400 MHz, CDCl_3_) δ 8.15–7.91 (m, 1H), 7.62 (dd, *J* = 42.8, 11.8 Hz, 1H), 7.54–7.50 (m, 1H), 7.18–7.04 (m, 2H), 5.43–5.28 (m, 1H), 5.28–5.17 (m, 1H), 4.53 (td, *J* = 9.9, 3.8 Hz, 1H), 4.15–4.07 (m, 2H), 3.82–3.55 (m, 2H), 2.47–1.98 (m, 4H), 1.88–1.63 (m, 2H), 1.49 (m 1H), 1.27–1.23 (m, 3H), 0.91 (m, 6H);^13^C NMR (δ) (101 MHz, CDCl_3_) δ 173.36, 156.77, 156.34, 148.16, 147.17, 135.32, 133.35, 130.07, 123.61, 122.59, 94.58, 61.01, 54.29, 50.70, 47.31, 41.66, 25.21, 24.43, 23.18, 21.39, 14.37; MS (ESI) *m/z =* 525.0 [M+H]^+^.

**Ethyl ((*R*)-1-((*S*)-2-(5-(4-iodophenyl)-1*H*-imidazol-2-yl)pyrrolidin-1-yl)-4-methyl-1-oxopentan-2-yl) carbamate (16).** It was prepared by coupling compound (**D_2_**) with ethyl-*D*-leucine carbamate using the previous general procedure, and it was purified using EtOAc; yellow semisolid; yield: 1.0 g (80%); ^1^H NMR (400 MHz, CDCl_3_) δ 7.65 (dd, *J* = 8.4, 3.7 Hz, 2H), 7.51–7.30 (m, 2H), 7.20 (s, 1H), 5.36 (d, *J* = 8.8 Hz, 1H), 5.24–5.16 (m, 1H), 4.54–4.33 (m, 1H), 4.10 (d, *J* = 7.0 Hz, 2H), 3.85–3.63 (m, 2H), 1.67 (ddd, *J* = 91.9, 49.0, 43.6 Hz, 4H), 1.23 (td, *J* = 7.2, 5.0 Hz, 6H), 0.93–0.86 (m, 6H); ^13^C NMR (101 MHz, CDCl_3_) δ 165.92, 156.69, 148.42, 142.31, 137.80, 126.77, 126.61, 119.22, 103.53, 61.34, 54.58, 51.08, 47.64, 38.74, 27.87, 23.45, 23.09, 21.65, 14.67; MS (ESI) *m/z =* 525.0 [M+H]^+^.

**Methyl ((*S*)-2-((*S*)-2-(5-(3-iodophenyl)-1*H*-imidazol-2-yl)pyrrolidin-1-yl)-2-oxo-1-phenylethyl)carbamate (17).** It was prepared by coupling compound **(D_1_)** with methyl-*L*-phenylglycine carbamate using the previous general procedure, and it was purified using DCM: MeOH 20:1; yellow semisolid; yield: 0.8 g (64%); ^1^H NMR (400 MHz, CDCl_3_) δ 7.67 (ddd, *J* = 24.0, 10.9, 5.6 Hz, 1H), 7.57–7.45 (m, 1H), 7.44–7.29 (m, 8H), 5.98–5.82 (m, 1H), 5.33–5.21 (m, 1H), 3.65 (s, 3H), 3.17 (s, 2H), 1.33 (d, *J* = 5.7 Hz, 5H); MS (ESI) *m*/*z* = 531.0 [M+H]^+^.

**Methyl ((*S*)-2-((*S*)-2-(5-(4-iodophenyl)-1*H*-imidazol-2-yl)pyrrolidin-1-yl)-2-oxo-1-phenylethyl) carbamate (18).** It was prepared by coupling compound (**D_2_**) with methyl-*L*-phenylglycine carbamate using the previous general procedure, and it was purified using DCM: MeOH 100:4; yellow semisolid; yield: 0.9 g (72%); ^1^H NMR (400 MHz, CDCl_3_) δ 7.64 (dd, *J* = 19.9, 8.4 Hz, 2H), 7.36 (ddd, *J* = 30.7, 17.8, 5.5 Hz, 6H), 7.24–7.12 (m, 2H), 6.11 (t, *J* = 13.6 Hz, 1H), 5.48 (d, *J* = 7.9 Hz, 1H), 5.29–5.24 (m, 1H), 3.75–3.65 (m, 2H), 3.64 (s, 3H), 2.19–1.85 (m, 4H); ^13^C NMR (101 MHz, CDCl_3_) δ 170.49, 157.94, 156.40, 147.58, 137.72, 136.40, 129.30, 128.78, 128.19, 127.69, 126.50, 114.36, 91.81, 58.31, 56.94, 52.49, 47.33, 27.95, 25.22; MS (ESI) *m*/*z* = 531.0 [M+H]^+^.

**Methyl ((*R*)-2-((*S*)-2-(5-(3-iodophenyl)-1*H*-imidazol-2-yl)pyrrolidin-1-yl)-2-oxo-1-phenylethyl)carbamate (19).** It was prepared by coupling compound (**D_1_**) with methyl-*D*-phenylglycine carbamate using the previous general procedure, and it was purified using DCM: MeOH; 20:1; yellow semisolid; yield: 0.5 g (40%); ^1^H NMR (400 MHz, CDCl_3_) δ 7.90 (t, *J* = 36.0 Hz, 1H), 7.55 (dd, *J* = 26.6, 9.3 Hz, 1H), 7.39–7.28 (m, 8H), 6.06–6.00 (m, 1H), 5.39–5.31 (m, 1H), 5.18 (s, 1H), 3.62 (s, 3H), 3.23–2.87 (m, 2H), 2.57–1.68 (m, 4H); ^13^C NMR (101 MHz, CDCl_3_) δ 173.57, 157.16, 156.01, 137.33, 133.80, 132.45, 130.46, 129.88, 129.23, 128.54, 127.92, 126.98 124.51, 116.06, 94.43, 58.09, 57.70, 52.79, 52.15, 38.45, 36.65; MS (ESI) *m*/*z* = 531.0 [M+H]^+^.

**Methyl ((R)-2-((S)-2-(5-(4-iodophenyl)-1*H*-imidazol-2-yl)pyrrolidin-1-yl)-2-oxo-1-phenylethyl) carbamate (20).** It was prepared by coupling compound (**D_2_**) with methyl-*D*-phenylglycine carbamate using the previous general procedure, and it was purified using DCM: MeOH 100: 4; brown semisolid; yield: 0.9 g (72%); ^1^H NMR (400 MHz, CDCl_3_) δ 7.68 (d, *J* = 8.1 Hz, 2H), 7.40–7.34 (m, 8H), 5.86 (d, *J* = 4.8 Hz, 1H), 5.38 (d, *J* = 5.3 Hz, 1H), 5.33 (d, *J* = 7.0 Hz, 1H), 3.64 (s, 3H), 3.57 (m, 2H), 2.38–1.88 (m, 4H); ^13^C NMR (101 MHz, CDCl_3_) δ 168.37, 157.31, 151.80, 148.41, 138.47, 132.98, 129.71, 129.05, 128.27, 127.35, 125.66, 118.94, 95.82, 58.11, 53.21, 52.60, 47.33, 31.16, 24.72; MS (ESI) *m/z =* 531.0 [M+H]^+^.

###### Procedures of Sonogashira Reaction Followed by Desilylation and Dimerization, Compounds (**1a**–**10a**) and Compounds (**1b**–**10b**)

Compounds (**1**–**20**) were mixed with bis-(triphenylphosphine) palladium II chloride (5% mmol) and copper (I) iodide (10% mmol) in DMF (5 mL). TEA was added (1.5 equiv.), followed by trimethylsilylacetylene (TMS) (1.15 equiv.). The mixture was left to stir at 70 °C for 4 h under argon. Afterwards, the reaction mixture was left to cool, concentrated under vacuum, washed with saturated aqueous NaCl solution, and extracted with EtOAc (50 mL × 3). The organic layer was separated and dried over MgSO_4_, filtered then concentrated under reduced pressure to be used in the next step without further purification.

To the resulting compounds from the Sonogashira reaction step, distilled water (2 mL), methanol (10 mL), and potassium carbonate (4 mmol, 0.55 g) were added. The reaction mixture was stirred at 55 °C overnight. Then, it was concentrated under vacuum, washed with distilled water, and extracted with EtOAc (50 mL × 3). The combined organic layers were dried over MgSO_4_, filtered then concentrated under vacuum. The final product was later purified using silica gel in column chromatography.

**Dimethyl ((2*S*,2′*S*)-((2*S*,2′*S*)-((buta-1,3-diyne-1,4-diylbis(3,1-phenylene))bis(1*H*-imidazole-5,2-diyl))bis(pyrrolidine-2,1-diyl))bis(3-methyl-1-oxobutane-1,2diyl))dicarbamate (1a).** It was prepared using a Sonogashira reaction, followed by dimerization of compound 1, and it was purified using DCM: MeOH 20:1; yellow semisolid; yield: 11%; ^1^H NMR (400 MHz, CDCl_3_) δ 7.57–7.52 (m, 2H), 7.46 (ddd, *J* = 5.7, 3.8, 2.1 Hz, 2H), 7.34–7.28 (m, 4H), 7.23–7.19 (m, 2H), 5.60 (d, *J* = 9.1 Hz, 2H), 5.24–5.21 (m, 2H), 4.31 (dd, *J* = 9.0, 6.9 Hz, 2H), 3.68 (s, 6H), 3.63 (d, *J* = 14.1 Hz, 4H), 2.21–2.12 (m, 3H), 2.09–2.04 (m, 3H), 2.03–1.92 (m, 4H), 0.86 (d, *J* = 6.7 Hz, 12H); ^13^C NMR (101 MHz, CDCl_3_) δ 172.47, 158.29, 156.94, 148.12, 132.68, 131.94, 130.08, 128.39, 128.08, 125.04, 122.18, 115.33, 82.22, 74.81, 57.47, 54.18, 52.18, 47.71, 30.96, 27.25, 25.19, 19.04; MS (ESI) *m*/*z* = 787.0 [M+H]^+^.

**Dimethyl ((2*S*,2′*S*)-((2*S*,2′*S*)-((buta-1,3-diyne-1,4-diylbis(4,1-phenylene))bis(1*H*-imidazole-5,2-diyl))bis(pyrrolidine-2,1-diyl))bis(3-methyl-1-oxobutane-1,2-diyl))dicarbamate (1b).** It was prepared using a Sonogashira reaction, followed by dimerization of compound 2, and it was purified using EtOAc: MeOH 100:3; light brown solid; yield: 12%; mp = 116–118 °C; ^1^H NMR (400 MHz, CDCl_3_) δ 7.77–7.28 (m, 8H), 7.21–6.95 (m, 2H), 5.66 (d, *J* = 7.4 Hz, 2H), 5.29–5.19 (m, 2H), 4.37–3.98 (m, 2H), 3.67 (s, 6H), 3.22–2.74 (m, 4H), 2.44–2.11 (m, 5H), 2.11–1.87 (m, 5H), 0.92 (t, *J* = 26.6 Hz, 12H); ^13^C NMR (101 MHz, CDCl_3_) δ 172.53, 157.22, 148.62, 136.66, 135.34, 132.87, 124.65, 119.56, 118.72, 82.35, 74.31, 58.29, 57.83, 52.47, 48.03, 31.32, 25.35, 19.32, 17.77; MS (ESI) *m*/*z* = 787.0 [M+H]^+^; HRMS *m*/*z* = 785.3783 (calculated = 785.3781) (M-H)^−^.

**Dimethyl ((2*R*,2′*R*)-((2*S*,2′*S*)-((buta-1,3-diyne-1,4-diylbis(3,1-phenylene))bis(1*H*-imidazole-5,2-diyl))bis(pyrrolidine-2,1-diyl))bis(3-methyl-1-oxobutane-1,2-diyl))dicarbamate (2a).** It was prepared using a Sonogashira reaction, followed by dimerization of compound 3, and it was purified using DCM: MeOH 20:1; light brown solid; yield: 10%; mp = 103.0–105.0 °C; ^1^H NMR (400 MHz, CDCl_3_) δ 7.86 (s, 2H), 7.66 (t, *J* = 11.4 Hz, 2H), 7.37 (ddd, *J* = 7.7, 4.6, 1.4 Hz, 2H), 7.30 (t*, J* = 7.7 Hz, 2H), 7.21 (d, *J* = 9.7 Hz, 2H), 5.53 (d, *J* = 7.7 Hz, 2H), 5.31–5.28 (m, 2H), 4.21 (t*, J* = 7.6 Hz, 2H), 3.72–3.63 (m, 6H), 3.63–3.44 (m, 4H), 2.25–2.01 (m, 9H), 1.97 (d, *J* = 16.4 Hz, 1H), 1.02 (dd, *J* = 6.6, 2.0 Hz, 12H); ^13^C NMR (101 MHz, CDCl_3_) δ 171.99, 157.36, 151.33, 148.28, 135.03, 133.28, 130.38, 128.55, 125.45, 121.81, 119.07, 117.31, 81.50, 73.68, 58.21, 54.97, 52.38, 47.35, 30.57, 29.13, 24.47, 19.24; MS (ESI) *m*/*z* = 787.0 [M+H]^+^; HRMS *m*/*z* = 785.3777 (calculated = 785.3781) (M-H)^−^.

**Dimethyl ((2*R*,2′*R*)-((2*S*,2′*S*)-((buta-1,3-diyne-1,4-diylbis(4,1-phenylene))bis(1*H*-imidazole-5,2-diyl))bis(pyrrolidine-2,1-diyl))bis(3-methyl-1-oxobutane-1,2-diyl))dicarbamate (2b).** It was prepared using a Sonogashira reaction, followed by dimerization of compound 4, and it was purified using DCM: MeOH 100:5; light brown solid; yield: 14%; mp: 120–122 °C; ^1^H NMR (400 MHz, CDCl_3_) δ 7.63 (d, *J* = 7.7 Hz, 4H), 7.47 (d, *J* = 8.2 Hz, 4H), 7.18 (s, 2H), 5.56 (d, *J* = 7.3 Hz, 2H), 5.30 (d, *J* = 4.5 Hz, 2H), 4.21 (t, *J* = 7.5 Hz, 2H), 3.79 (d, *J* = 91.2 Hz, 2H), 3.63 (s, 6H), 3.58 (d*, J* = 9.0 Hz, 2H), 2.34–2.09 (m, 5H), 2.09–1.86 (m, 5H), 1.06–0.97 (m, 12H); ^13^C NMR (101 MHz, CDCl_3_) δ 172.17, 157.49, 149.27, 148.57, 136.92, 132.81, 124.49, 119.58, 115.89, 82.13, 74.31, 58.38, 55.16, 52.52, 47.55, 30.76, 29.35, 24.65, 18.08; MS (ESI) *m/z =* 787.0 [M+H]^+^; HRMS *m*/*z* = 785.3784 (calculated = 785.3781) (M-H)^−^.

**Diethyl ((2*S*,2′*S*)-((2*S*,2′*S*)-((buta-1,3-diyne-1,4-diylbis(3,1-phenylene))bis(1*H*-imidazole-5,2-diyl))bis(pyrrolidine-2,1-diyl))bis(3-methyl-1-oxobutane-1,2-diyl))dicarbamate (3a).** It was prepared using a Sonogashira reaction, followed by dimerization of compound 5, and it was purified using DCM: MeOH 20:1; yellow solid; yield: 14%; mp: 124 –126.7 °C; ^1^H NMR (400 MHz, CDCl_3_) δ 7.97 (s, 2H), 7.57–7.50 (m, 4H), 7.20–7.01 (m, 4H), 5.61 (t, *J* = 47.2 Hz, 2H), 5.22 (dd, *J* = 8.1, 4.0 Hz, 2H), 4.32–4.27 (m, 2H), 4.10 (dd, *J* = 8.0, 3.9 Hz, 4H), 3.87–3.63 (m, 4H), 2.35–1.94 (m, 10H), 1.07–0.97 (m, 6H), 0.91–0.84 (m, 12H); ^13^C NMR (101 MHz, CDCl_3_) δ 172.59, 165.59, 156.54, 148.19, 147.36, 135.38, 133.32, 130.08, 123.88, 123.56, 118.80, 82.61, 74.65, 61.02, 57.39, 54.18, 47.71, 30.86, 25.14, 18.91, 17.41, 14.36; MS (ESI) *m*/*z* = 815.0 [M+H]^+^.

**Diethyl ((2*S*,2′*S*)-((2*S*,2′*S*)-((buta-1,3-diyne-1,4-diylbis(4,1-phenylene))bis(1*H*-imidazole-5,2-diyl))bis(pyrrolidine-2,1-diyl))bis(3-methyl-1-oxobutane-1,2-diyl))dicarbamate (3b).** It was prepared using a Sonogashira reaction, followed by dimerization of compound 6, and it was purified using EtOAc: MeOH 100:2.5; dark brown solid; yield: 17%; mp =139–141 °C; ^1^H NMR (400 MHz, CDCl_3_) δ 7.77–7.29 (m, 8H), 7.18 (d, *J* = 22.4 Hz, 2H), 5.46 (d, *J* = 9.0 Hz, 2H), 5.23 (dd, *J* = 7.7, 3.7 Hz, 2H), 4.28 (dd, *J* = 42.7, 35.9 Hz, 2H), 4.13–4.04 (m, 4H), 3.81 (dd, *J* = 38.3, 31.6 Hz, 4H), 2.36–1.95 (m, 10H), 1.25 (t, *J* = 7.0 Hz, 6H), 0.91 (dd, *J* = 27.1, 16.0 Hz, 12H); ^13^C NMR (101 MHz, CDCl_3_) δ 172.74, 156.85, 150.04, 141.81, 132.93, 127.58, 125.65, 124.50, 110.69, 82.64, 74.22, 61.33, 57.70, 54.66, 48.06, 31.30, 25.41, 19.39, 17.74, 14.70; MS (ESI) *m*/*z* = 815.0 [M+H]^+^; HRMS *m*/*z* = 813.4100 (calculated = 813.4094) (M-H)^−^.

**Dibutyl ((2*S*,2′*S*)-((2*S*,2′*S*)-((buta-1,3-diyne-1,4-diylbis(3,1-phenylene))bis(1*H*-imidazole-5,2-diyl))bis(pyrrolidine-2,1-diyl))bis(3-methyl-1-oxobutane-1,2-diyl))dicarbamate (4a).** It was prepared using a Sonogashira reaction, followed by dimerization of compound 7, and it was purified using DCM: MeOH 20:1; light brown semisolid; yield: 15%; ^1^H NMR (400 MHz, CDCl_3_) δ 7.79–7.66 (m, 5H), 7.61–7.47 (m, 5H), 5.44 (d, *J* = 9.1 Hz, 2H), 5.29– 5.09 (m, 2H), 4.21 (t, *J* = 6.0 Hz, 6H), 4.04 (d, *J* = 8.6 Hz, 4H), 2.37–1.81 (m, 10H), 1.67 (dd, *J* = 12.0, 5.9 Hz, 4H), 1.60–1.50 (m, 4H), 0.93–0.89 (m, 18H); ^13^C NMR (101 MHz, CDCl_3_) δ 167.58, 160.22, 154.81, 141.70, 133.68, 132.24, 131.95, 130.70, 128.60, 126.47, 125.68, 124.16, 81.60, 74.10, 67.96, 65.03, 60.00, 45.90, 38.52, 30.16, 28.73, 23.54, 22.80, 13.88, 10.78; MS (ESI) *m*/*z* = 871.0 [M+H]^+^.

**Dibutyl ((2*S*,2′*S*)-((2*S*,2′*S*)-((buta-1,3-diyne-1,4-diylbis(4,1-phenylene))bis(1*H*-imidazole-5,2-diyl))bis(pyrrolidine-2,1-diyl))bis(3-methyl-1-oxobutane-1,2-diyl))dicarbamate (4b).** It was prepared using a Sonogashira reaction, followed by dimerization of compound 8, and it was purified using EtOAc: MeOH 100:2; light brown solid; yield: 21%; mp =136–138 °C; ^1^H NMR (400 MHz, CDCl_3_) δ 7.74–7.28 (m, 8H), 7.21–7.07 (m, 2H), 5.50 (t, *J* = 9.7 Hz, 2H) 5.29–5.20 (m, 2H), 4.36–4.13 (m, 2H), 4.04 (dt, *J* = 8.7, 6.0 Hz, 4H), 3.82 (dd, *J* = 35.8, 28.6 Hz, 4H), 2.39–2.15 (m, 5H), 2.13–1.98 (m, 5H), 1.61–1.56 (m, 4H), 1.40–1.34 (m, 4H), 0.90 (dd, *J* = 15.3, 7.6 Hz, 18H); ^13^C NMR (101 MHz, CDCl_3_) δ 172.87, 157.21, 152.52, 149.10, 133.12, 127.17, 124.89, 124.58, 119.92, 82.57, 74.79, 65.51, 57.97, 54.95, 48.31, 31.55, 31.38, 25.60, 19.62, 19.41, 18.04, 14.12; MS (ESI) *m*/*z* = 871.0 [M+H]^+^; HRMS *m*/*z* = 869.4715 (calculated = 869.4720) (M-H)^−^.

**Dimethyl ((2*S*,2′*S*)-((2*S*,2′*S*)-((buta-1,3-diyne-1,4-diylbis(3,1-phenylene))bis(1*H*-imidazole-5,2-diyl))bis(pyrrolidine-2,1-diyl))bis(4-methyl-1-oxopentane-1,2-diyl))dicarbamate (5a).** It was prepared using a Sonogashira reaction, followed by dimerization of compound 9, and it was purified using DCM: MeOH; 20:1; light brown solid; yield: 14%; mp= 103.1–105.3 °C; ^1^H NMR (400 MHz, CDCl_3_) δ 7.70 (d, *J* = 7.3 Hz, 1H), 7.65 (s, 1H), 7.31 (ddd, *J* = 11.8, 10.9, 6.0 Hz, 6H), 7.21 (d*, J* = 7.6 Hz, 2H), 5.54 (t, *J* = 9.8 Hz, 2H), 5.25–5.17 (m, 2H), 4.53 (dd, *J* = 12.5, 6.2 Hz, 2H), 3.67 (s, 6H), 3.47 (d, *J* = 2.8 Hz, 4H), 1.81–1.48 (m, 8H), 1.47–1.33 (m, 4H), 1.31–1.21 (m, 2H), 0.91 (dd, *J* = 14.7, 5.1 Hz, 12H);^13^C NMR (101 MHz, CDCl_3_) δ 173.21, 158.39, 156.78, 148.16, 131.08, 130.38, 128.54, 125.24, 121.86, 120.35, 119.49, 117.42, 81.46, 74.39, 54.31, 52.14, 50.85, 47.29, 41.52, 25.16, 24.41, 23.14, 21.32; MS (ESI) *m*/*z* = 815.0 [M+H]^+^; HRMS *m*/*z* = 813.4089 (calculated = 813.4094) (M-H)^−^.

**Dimethyl ((2*S*,2′*S*)-((2*S*,2′*S*)-((buta-1,3-diyne-1,4-diylbis(4,1-phenylene))bis(1*H*-imidazole-5,2-diyl))bis(pyrrolidine-2,1-diyl))bis(4-methyl-1-oxopentane-1,2-diyl))dicarbamate (5b).** It was prepared using a Sonogashira reaction, followed by dimerization of compound 10, and it was purified using EtOAc: MeOH 100:4.5; light brown solid; yield: 21%; mp = 124–126 °C; ^1^H NMR (400 MHz, CDCl_3_) δ 7.60 (d, *J* = 88.4 Hz, 4H), 7.48 (s, 4H), 7.21 (d, *J* = 5.2 Hz, 2H), 5.47 (d, *J* = 7.8 Hz, 2H), 5.22 (d, *J* = 7.2 Hz, 2H), 4.60–4.50 (m, 2H), 3.68 (s, 6H), 3.64–3.54 (m, 4H), 2.15 (ddd*, J* = 28.9, 15.1, 5.8 Hz, 8H), 1.70 (m, 4H), 1.53–1.47 (m, 2H), 0.94–0.87 (m, 12H); ^13^C NMR (101 MHz, CDCl_3_) δ 173.87, 157.08, 152.56, 146.80, 139.02, 132.93, 127.18, 124.71, 119.45, 82.54, 74.99, 54.70, 52.49, 51.14, 47.63, 38.74, 25.47, 24.73, 23.49, 21.70; MS (ESI) *m*/*z* = 815.0 [M+H]^+^; HRMS *m*/*z* = 813.4101 (calculated = 813.4094) (M-H)^−^.

**Dimethyl ((2*R*,2′*R*)-((2*S*,2′*S*)-((buta-1,3-diyne-1,4-diylbis(3,1-phenylene))bis(1*H*-imidazole-5,2-diyl))bis(pyrrolidine-2,1-diyl))bis(4-methyl-1-oxopentane-1,2-diyl))dicarbamate (6a).** It was prepared using a Sonogashira reaction, followed by dimerization of compound 11, and it was purified using DCM: MeOH 20:1; yellow solid; yield: 12%; mp =111–113 °C; ^1^H NMR (400 MHz, CDCl_3_) δ 7.70 (s, 2H), 7.35 (t, *J* = 5.8 Hz, 3H), 7.29 (dd, *J* = 12.6, 5.0 Hz, 3H), 7.20 (d, *J* = 6.4 Hz, 2H), 5.52 (d, *J* = 8.7 Hz, 2H), 5.26–5.21 (m, 2H), 4.54 (td, *J* = 9.8, 3.7 Hz, 2H), 3.68 (s, 6H), 3.56 (dd, *J* = 49.1, 14.3 Hz, 4H), 2.20–1.96 (m, 8H), 1.83–1.64 (m, 4H), 1.49 (td, *J* = 10.1, 5.3 Hz, 2H), 0.95–0.88 (m, 12H); ^13^C NMR (101 MHz, CDCl_3_) δ 171.59, 156.76, 151.03, 148.10, 130.39, 128.83, 128.55, 126.74, 125.26, 121.81, 120.99, 115.14, 81.54, 73.86, 54.32, 52.15, 50.84, 47.31, 41.62, 27.38, 25.19, 24.43, 21.37; MS (ESI) *m*/*z* = 815.0 [M+H]^+^; HRMS *m*/*z* = 813.4088 (calculated = 813.4094) (M-H)^−^.

**Dimethyl ((2*R*,2′*R*)-((2*S*,2′*S*)-((buta-1,3-diyne-1,4-diylbis(4,1-phenylene))bis(1*H*-imidazole-5,2-diyl))bis(pyrrolidine-2,1-diyl))bis(4-methyl-1-oxopentane-1,2-diyl))dicarbamate (6b).** It was prepared using a Sonogashira reaction, followed by dimerization of compound 12, and it was purified using EtOAc: MeOH 100:2.5; brown solid; MP = 129–131 °C; yield: 25%; ^1^H NMR (400 MHz, CDCl_3_) δ 7.63 (dd, *J* = 71.0, 14.1 Hz, 4H), 7.46 (dd, *J* = 15.5, 4.4 Hz, 4H), 7.18 (d, *J* = 17.3 Hz, 2H), 5.55 (d, *J* = 8.6 Hz, 2H), 5.24 (dd, *J* = 17.3, 12.1 Hz, 2H), 4.55 (td*, J* = 9.6, 3.8 Hz, 2H), 3.89–3.69 (m, 4H), 3.69–3.65 (s, 6H), 3.13–2.86 (m, 2H), 2.21–2.09 (m, 6H), 1.70–1.43 (m, 4H), 1.35 (dd, *J* = 12.4, 5.0 Hz, 2H), 0.92 (dd, *J* = 20.5, 6.3 Hz, 12H); ^13^C NMR (101 MHz, CDCl_3_) δ 173.48, 158.59, 157.07, 148.63, 132.94, 127.54, 124.68, 124.48, 119.77, 116.97, 81.89, 74.50, 54.72, 52.47, 51.17, 47.63, 41.88, 25.41, 24.73, 23.48, 21.68; MS (ESI) *m*/*z* = 815.0 [M+H]^+^; HRMS *m*/*z* = 813.4091 (calculated = 813.4094) (M-H)^−^.

**Diethyl ((2*S*,2′*S*)-((2*S*,2′*S*)-((buta-1,3-diyne-1,4-diylbis(3,1-phenylene))bis(1*H*-imidazole-5,2-diyl))bis(pyrrolidine-2,1-diyl))bis(4-methyl-1-oxopentane-1,2-diyl))dicarbamate (7a).** It was prepared using a Sonogashira reaction, followed by dimerization of compound 13, and it was purified using DCM: MeOH 20:1; light brown semi-solid; yield: 14%; ^1^H NMR (400 MHz, CDCl_3_) δ 7.83 (s, 1H), 7.65 (d, *J* = 7.0 Hz, 2H), 7.37–7.29 (m, 5H), 7.20 (s, 2H), 5.42 (d, *J* = 8.0 Hz, 2H), 5.24 (s, 2H), 4.53 (s, 2H), 4.11 (d, *J* = 6.4 Hz, 4H), 3.56 (d, *J* = 24.2 Hz, 4H), 2.25 (d, *J* = 85.1 Hz, 8H), 1.70 (s, 4H), 1.48 (d, *J* = 10.1 Hz, 2H), 1.24 (s, 6H), 0.95–0.89 (m, 12H); ^13^C NMR (101 MHz, CDCl_3_) δ 173.30, 156.34, 155.62, 148.17, 134.71, 133.38, 130.37, 129.94, 128.56, 125.27, 121.83, 118.01, 81.50, 73.68, 61.00, 54.31, 50.73, 47.29, 41.63, 25.19, 24.43, 23.16, 21.37, 14.37; MS (ESI) *m*/*z* = 843.0 [M+H]^+^; HRMS *m*/*z* = 841.4398 (calculated = 841.4407) (M-H)^−^.

**Diethyl ((2S,2′S)-((2S,2′S)-((buta-1,3-diyne-1,4-diylbis(4,1-phenylene))bis(1*H*-imidazole-5,2-diyl))bis(pyrrolidine-2,1-diyl))bis(4-methyl-1-oxopentane-1,2-diyl))dicarbamate (7b).** It was prepared using a Sonogashira reaction, followed by dimerization of compound 14, and it was purified using DCM: MeOH 100: 2.5; brown solid; yield: 13%; mp = 118–120 °C; ^1^H NMR (400 MHz, CDCl_3_) δ 7.79–7.29 (m, 8H), 7.19 (s, 2H), 5.62–5.48 (m, 2H), 5.30–4.99 (m, 2H), 4.21 (dd, *J* = 91.9, 85.2 Hz, 6H), 3.87–3.53 (m, 4H), 2.40–1.93 (m, 7H), 1.80–1.37 (m, 7H), 1.24 (d, *J* = 7.1 Hz, 6H), 0.97–0.89 (m, 12H); ^13^C NMR (101 MHz, CDCl_3_) δ 169.07, 156.69, 151.88, 141.95, 132.98, 132.07, 124.51, 121.70, 117.21, 82.14, 72.30, 61.35, 54.73, 51.11, 47.70, 41.92, 24.79, 23.51, 23.12, 21.71, 14.71; MS (ESI) *m*/*z*: 843.0 [M+H]^+^.

**Diethyl ((2*R*,2′*R*)-((2*S*,2′*S*)-((buta-1,3-diyne-1,4-diylbis(3,1-phenylene))bis(1H-imidazole-5,2-diyl))bis(pyrrolidine-2,1-diyl))bis(4-methyl-1-oxopentane-1,2-diyl))dicarbamate (8a).** It was prepared using a Sonogashira reaction, followed by dimerization of compound 15, and it was purified using DCM: MeOH; 20:1; yellow solid; yield: 13%; mp = 109.0–111.0 °C; ^1^H NMR (400 MHz, CDCl_3_) δ 7.70 (s, 1H), 7.68–7.64 (m, 1H), 7.35 (t, *J* = 6.8 Hz, 4H), 7.30 (d, *J* = 7.5 Hz, 2H), 7.21 (d, *J* = 6.4 Hz, 2H), 5.40 (d, *J* = 8.7 Hz, 2H), 5.26–5.22 (m, 2H), 4.54 (td, *J* = 9.8, 3.5 Hz, 2H), 4.13–4.09 (m, 4H), 3.58 (d, *J* = 26.5 Hz, 4H), 2.24–2.06 (m, 8H), 2.04–1.81 (m, 4H), 1.80–1.53 (m, 6H), 1.52–1.47 (m, 2H), 0.92 (dd, *J* = 15.4, 5.6 Hz, 12H); ^13^C NMR (101 MHz, CDCl_3_) δ 173.30, 162.65, 156.36, 134.58, 130.43, 128.57, 127.40, 125.38, 124.06, 121.84, 116.25, 114.57, 81.41, 73.53, 61.01, 54.30, 50.72, 47.31, 41.68, 25.20, 24.44, 23.18, 21.38, 14.37; MS (ESI) *m*/*z* = 843.0 [M+H]^+^; HRMS *m*/*z* = 841.4401 (calculated = 841.4407) (M-H)^−^.

**Diethyl ((2*R*,2′*R*)-((2*S*,2′*S*)-((buta-1,3-diyne-1,4-diylbis(4,1-phenylene))bis(1*H*-imidazole-5,2-diyl))bis(pyrrolidine-2,1-diyl))bis(4-methyl-1-oxopentane-1,2-diyl))dicarbamate (8b).** It was prepared using a Sonogashira reaction, followed by dimerization on compound 16, and it was purified using DCM: MeOH 100:5; light brown solid; yield: 17%; mp = 129–132 °C; ^1^H NMR (400 MHz, CDCl_3_) δ 7.75–7.39 (m, 8H), 7.20 (d, *J* = 15.0 Hz, 2H), 5.65–5.51 (m, 2H), 5.39–5.01 (m, 2H), 4.55 (d, *J* = 9.1 Hz, 2H), 4.11 (dd, *J* = 13.2, 6.5 Hz, 4H), 3.85–3.47 (m, 4H), 2.41–1.94 (m, 8H), 1.70–1.44 (m, 4H), 1.41–1.32 (m, 2H), 1.24 (t, *J* = 7.0 Hz, 6H), 0.96–0.87 (m, 12H); ^13^C NMR (101 MHz, CDCl_3_) δ 173.60, 156.67, 155.52, 140.75, 132.95, 124.67, 124.51, 122.70, 117.63, 81.99, 74.50, 61.33, 54.71, 51.05, 47.65, 41.99, 25.45, 24.76, 23.50, 21.71, 14.69; MS (ESI) *m*/*z* = 843.0 [M+H]^+^.

**Dimethyl ((1*S*,1′*S*)-((2*S*,2′*S*)-((buta-1,3-diyne-1,4-diylbis(3,1-phenylene))bis(1*H*-imidazole-5,2-diyl))bis(pyrrolidine-2,1-diyl))bis(2-oxo-1-phenylethane-2,1-diyl))dicarbamate (9a).** It was prepared using a Sonogashira reaction, followed by dimerization of compound 17, and it was purified using DCM: MeOH 20:1; light brown solid; yield: 10%; mp = 125–127 °C; ^1^H NMR (400 MHz, CDCl_3_) δ 7.86 (d, *J* = 18.0 Hz, 1H), 7.78–7.60 (m, 4H), 7.54 (dd, *J* = 11.2, 5.1 Hz, 1H), 7.49–7.33 (m, 12H), 7.31–7.29 (m, 2H), 6.25–6.05 (m, 2H), 5.44 (dd, *J* = 30.4, 7.3 Hz, 2H), 5.29 (d, *J* = 2.8 Hz, 2H), 3.65 (d, *J* = 9.2 Hz, 6H), 3.42–3.11 (m, 4H), 2.24–1.95 (m, 8H); ^13^C NMR (101 MHz, CDCl_3_) δ 170.16, 156.11, 155.42, 136.19, 131.84, 130.37, 129.02, 128.50, 127.91, 127.39, 125.32, 121.85, 115.71, 114.80, 83.07, 73.71, 56.63, 574.76, 52.19, 47.01, 29.11, 24.96; MS (ESI) *m*/*z* = 855.0 [M+H]^+^; HRMS *m*/*z* = 853.3474 (calculated = 853.3468) (M-H)^−^.

**Dimethyl ((1*S*,1′*S*)-((2*S*,2′*S*)-((buta-1,3-diyne-1,4-diylbis(4,1-phenylene))bis(1*H*-imidazole-5,2-diyl))bis(pyrrolidine-2,1-diyl))bis(2-oxo-1-phenylethane-2,1-diyl))dicarbamate (9b).** It was prepared using a Sonogashira reaction, followed by dimerization of compound 18, and it was purified using DCM: MeOH 100:4; brown semisolid; yield: 15%; ^1^H NMR (400 MHz, CDCl_3_) δ 7.64 (dd, *J* = 19.9, 8.4 Hz, 4H), 7.36 (ddd, *J* = 30.7, 17.8, 5.5 Hz, 12H), 7.24–7.08 (m, 4H), 6.11 (t, *J* = 13.6 Hz, 2H), 5.48 (d, *J* = 7.9 Hz, 2H), 5.29–5.24 (m, 2H), 3.75–3.65 (m, 4H), 3.64 (s, 6H), 2.14 (dd, *J* = 11.7, 5.9 Hz, 3H), 2.11–1.83 (m, 5H);^13^C NMR (101 MHz, CDCl_3_) δ 156.55, 147.45, 142.77, 141.65, 132.82, 129.26, 128.00, 124.52, 123.81, 119.42, 116.76, 84.51, 74.36, 57.61, 47.12, 29.30, 22.96; MS (ESI) *m*/*z* = 855.0 [M+H]^+^; HRMS *m*/*z* = 853.3470 (calculated = 853.3468) (M-H)^−^.

**Dimethyl ((1*R*,1′*R*)-((2*S*,2′*S*)-((buta-1,3-diyne-1,4-diylbis(3,1-phenylene))bis(1*H*-imidazole-5,2-diyl))bis(pyrrolidine-2,1-diyl))bis(2-oxo-1-phenylethane-2,1-diyl))dicarbamate (10a).** It was prepared using a Sonogashira reaction, followed by dimerization of compound 19, and it was purified using DCM: MeOH 20:1; light brown solid; mp =126–128 °C; yield: 10%; ^1^H NMR (400 MHz, CDCl_3_) δ 7.60 (dd, *J* = 15.4, 8.4 Hz, 6H), 7.50 (d, *J* = 8.4 Hz, 1H), 7.40–7.27 (m, 12H), 7.15 (s, 3H), 6.13 (d, *J* = 7.8 Hz, 1H), 5.48 (d, *J* = 7.9 Hz, 2H), 5.31–5.19 (m, 3H), 3.72–3.68 (m, 2H), 3.61 (s, 6H), 3.25 (t, *J* = 8.5 Hz, 2H), 2.21–2.07 (m, 4H), 2.07–1.84 (m, 4H); ^13^C NMR (101 MHz, CDCl_3_) δ 173.57, 156.01, 148.13, 137.33, 133.90, 133.80, 130.46, 129.88, 129.23, 129.12, 128.54, 128.01, 127.92, 126.98, 124.51, 82.61, 72.59, 57.70, 52.79, 52.15, 46.86, 38.45, 24.31; MS (ESI) *m*/*z* = 855.0 [M+H]^+^; HRMS *m*/*z* = 853.3472 (calculated = 853.3468) (M-H)^−^.

**Dimethyl ((1*R*,1′*R*)-((2*S*,2′*S*)-((buta-1,3-diyne-1,4-diylbis(4,1-phenylene))bis(1*H*-imidazole-5,2-diyl))bis(pyrrolidine-2,1-diyl))bis(2-oxo-1-phenylethane-2,1-diyl))dicarbamate (10b).** It was prepared using a Sonogashira reaction, followed by dimerization of compound 20, and it was purified using DCM: MeOH 100:4; brown solid; yield: 15%; mp = 125–127 °C; δ 7.80 (s, 2H), 7.68 (d, *J* = 8.1 Hz, 4H), 7.40–7.34 (m, 14H), 5.86 (d*, J* = 4.8 Hz, 2H), 5.38 (d, *J* = 5.3 Hz, 2H), 5.33 (d, *J* = 7.0 Hz, 2H), 3.64 (s, 6H), 3.57 (m, 4H), 2.16 (s, 5H), 1.99–1.84 (m, 3H); ^13^C NMR (101 MHz, CDCl_3_) δ 169.34, 156.55, 147.45, 142.77, 141.65, 132.82, 129.26, 128.00, 124.52, 123.81, 119.42, 116.76, 84.51, 74.36, 60.34, 57.61, 51.13, 47.12, 29.30, 22.96; MS (ESI) *m*/*z* = 855.0 [M+H]^+^; HRMS *m*/*z* = 853.3472 (calculated = 853.3468) (M-H)^−^.

##### 3.4. Biological Assays

###### 3.4.1. Cell Lines and Plasmids

Huh5-2 [65] and Huh7-JFH1 [66] stable cell lines harbor the subgenomic HCV reporter replicon I389luc-ubi-neo/NS3-3′/Con1/5.1 (genotype 1b; strain Con1) and I389luc-ubi-neo/NS3-3′_dg_JFH1 (genotype 2a, JFH1 strain), respectively (kindly provided by R. Bartenschlager, Heidelberg University, Heidelberg, Germany). Huh7.5-3a and Huh7.5-4a stable cell lines [67] harbor the subgenomic HCV reporter replicon S52-SG (Feo) (AII) (genotype 3a; S52 strain) and ED43-SG (Feo) (VYG) (genotype 4a; ED43 strain) [68], respectively (viral constructs kindly provided by C.M. Rice, Rockefeller University, New York, NY, USA). These replicons carry as a first cistron firefly luciferase (F-Luc) and neomycin phosphotransferase genes under the translational control of the HCV internal ribosome entry site (IRES), followed by HCV NS3 to NS5B proteins under the control of the EMCV IRES.

Cells were cultured in high glucose (25 mM) Dulbecco’s modified minimal essential medium (Thermo Scientific, Waltham, MA, USA), supplemented with 2 mM L-glutamine, 0.1 mM non-essential amino acids, 100 U/mL penicillin, 100 µg/mL streptomycin, and 10% (*v*/*v*) fetal calf serum (referred to as complete DMEM). Complete DMEM was supplemented with 500 μg/mL G418 for Huh5-2, 1 mg/mL for Huh7-JFH1, 750 μg/mL G418 for Huh7.5-3a and 350 μg/mL G418 for Huh7.5-4a.

Plasmid pH77S.3/GLuc2A [69] (kindly provided by S.M. Lemon, University of North Carolina, Chapel Hill, NC, USA) encodes the full-length reporter HCV genome sequence of genotype 1a (strain H77) with adaptive mutations. The Gaussia luciferase (G-Luc) gene, followed by the FMDV2A sequence is included between HCV p7 and NS2.

###### 3.4.2. In Vitro Transcription

H77S.3/GLuc2A was linearized with XbaI and used for in vitro transcription, as described previously [70].

###### 3.4.3. Transfection with In Vitro Transcribed RNA

Electroporation with the full-length viral RNA H77S.3/GLuc2A into Huh7-Lunet cells was performed as described elsewhere [71]. In brief, 4 × 10^6^ cells were detached by trypsin and resuspended in Cytomix [72] containing 2 mM ATP and 5 mM glutathione, mixed with 10 μg of viral RNA, and electroporated with a Gene Pulser system (Bio-Rad, Hercules, CA, USA).

###### 3.4.4. Anti-HCV Assay

The anti-HCV assay in replicon cells was performed by seeding 1 × 10^4^ cells per well in a 96-well plate. After 24 h of incubation at 37 °C (5% CO_2_) and medium removal, serial dilutions of the test compounds in complete DMEM were added. Cells were lysed after 72 h, and F-Luc activity was measured. The anti-HCV assay for the full-length viral construct H77S.3/GLuc2A was performed after electroporation of Huh7-Lunet cells with viral RNA. Cells were seeded in 96-well plates, the medium was removed 24 h after electroporation, and serial dilutions of the compounds in complete DMEM were added. At 72 h post-treatment, cell supernatants were collected, and G-Luc activity was measured. In addition, the cells were lysed for total protein quantification.

Relative luminescence units (RLU) were expressed as a percentage of the respective units derived from DMSO-treated cells (control). The half-maximal effective concentration (EC_50_), defined as the concentration of compound that reduces luciferase signal by 50%, was calculated by dose-response curve analysis using Prism 5.0 software (GraphPad Software Inc., San Diego, CA, USA).

###### 3.4.5. Luciferase and Bradford Assays

The F-Luc activity was measured using the Luciferase Assay System (Promega, Madison, WI, USA), as recommended by the manufacturer. The G-Luc activity was measured using 12 μM coelenterazine (Promega) in assay buffer (50 mM potassium phosphate, pH 7.4, 500 mM NaCl, 1 mM EDTA). Measurements were carried out using a GloMax 20/20 single tube luminometer (Promega) for 10 s. The F-Luc activity was normalized to the total protein amount determined using the Bradford assay reagent (Pierce, Waltham, MA, USA).

###### 3.4.6. Cytotoxicity Assay

Intracellular ATP levels were measured to determine the cytotoxicity of the compounds in the treated cells. In 96-well plates, 10^4^ cells per well were seeded, incubated with serial dilutions of the compounds or DMSO as a control for 72 h at 37 °C (5% CO_2_) and lysed for ATP measurement. ATP levels were measured using the ViaLight HS BioAssay kit (Lonza, Basel, Switzerland) in a GloMax 20/20 single-tube luminometer (Promega) for 1 s, according to the manufacturer’s protocol. Values were normalized to total protein amounts and used to determine the compound concentration causing 50% cell death (CC_50_). Nonlinear regression analysis was performed using Prism 5.0 software (GraphPad Software Inc., San Diego, CA, USA).

###### 3.4.7. Indirect Immunofluorescence

An indirect immunofluorescence analysis of Con1 NS5A was performed as described previously [71]. Propidium iodide (Sigma-Aldrich, St. Louis, MO, USA) was used to stain the DNA. Images were obtained with a Leica TCS-SP8 Confocal Microscope (Wetzlar, Germany).

###### 3.4.8. Total RNA Extraction and Quantification of Viral Replicons

Extraction of total RNA from Huh5-2 cells was performed using TRIzol reagent (Ambion, Austin, TX, USA), according to the manufacturer’s instructions. Replicon RNA was quantified with reverse transcription (RT) and quantitative real-time polymerase chain reaction (qPCR). RT was performed using a Con1 IRES-specific primer (IRES-R: 5′-GGATTCGTGCTCATGGTGCA-3′) and Moloney Murine Leukemia Virus (MMLV) reverse transcriptase (Promega). For qPCR, primers specific for Con1 IRES (IRES-F: 5′-GGCCTTGTGGTACTGCCTGATA-3′ and IRES-R) and the housekeeping gene YWHAZ (YWHAZ-F: 5′-GCTGGTGATGACAAGAAAGG-3′ and YWHAZ-R: 5′-GGATGTGTTGGTTGCATTTCCT-3′) were used in reactions containing KAPA SYBR FAST qPCR Master Mix (Kapa Biosystems, Wilmington, MA, USA).

###### 3.4.9. Statistical Analysis

In all diagrams, bars represent the mean values of at least two independent experiments in triplicate. Error bars represent standard deviations. Only results subjected to statistical analysis using Student’s t-test with *p* ≤ 0.05 was considered statistically significant and presented. Statistical calculations were performed using Excel Microsoft Office^®^.

## 4. Conclusions

In the context of eradicating HCV infections, globally focused efforts are required with wider research approaches targeting not only drugs’ potency but equally important the emerging resistance and the pan-genotypic spectrum of such compounds. Evolving our previously identified potent DAAs bearing the core scaffolds 3,3′-(buta-1,3-diyne-1,4-diyl)dianiline and 4,4′-(buta-1,3-diyne-1,4-diyl)dianiline linked to different capping groups to act as NS5A inhibitors, we introduced cap modifications. Specifically, we replaced existing amide groups with imidazole rings, hoping to achieve better potency, but also increase genotypic coverage, as well as to attain good metabolic stability. Different capping groups were also linked to these modified cores to try various possible interactions inside the NS5A pocket. Among those, the *m*, *m*’ compound **10a** with the aromatic phenylglycine cap proved the most potent in the picomolar range (EC_50_ = 0.10 nM), while the second most active compound **5b**, *p*, *p’* with the aliphatic leucine cap was in the low nanomolar range (EC_50_ = 16.12 nM) among this series. Additionally, combining in silico and pharmacochemical drug-like calculations, we started setting solid foundations on revealing all concealed aspects of the HCV NS5A activity and offering rationalized interpretation of data. Proving that spatial conformation of the molecule inside the NS5A pocket and molecule length affects the interactions that take place between caps and protein. Since our data indicate the core length being in close correlation with whether an *m*- or *p*- substitution will be tolerated, higher length cores with *p*-,*p*’- substitution was not favoured in our hands. Therefore, it is important to study the change in caps together with the molecule conformation to achieve the best combination that gives the highest potency. Additionally, **10a**, the most potent among all, was tested against GT 1a, GT 2a, GT 3a, and GT 4a and showed an EC_50_ in the nanomolar range against all of them, showing very good coverage of different genotypes. These outcomes and the fact that it has good metabolic stability make compound **10a** a promising candidate for further study and testing.

## Figures and Tables

**Table 1:** Final compounds’ specific structural features.

Compound	Attachment to Core	Terminal Cap Stereochemistry	X	Y
**1a**	*meta*	*S*	–CH(CH_3_)_2_	–CH_3_
**2a**	*meta*	*R*	–CH(CH_3_)_2_	–CH_3_
**3a**	*meta*	*S*	–CH(CH_3_)_2_	–C_2_H_5_
**4a**	*meta*	*S*	–CH(CH_3_)_2_	–C_4_H_9_
**5a**	*meta*	*S*	–CH_2_CH(CH_3_)_2_	–CH_3_
**6a**	*meta*	*R*	–CH_2_CH(CH_3_)_2_	–CH_3_
**7a**	*meta*	*S*	–CH_2_CH(CH_3_)_2_	–C_2_H_5_
**8a**	*meta*	*R*	–CH_2_CH(CH_3_)_2_	–C_2_H_5_
**9a**	*meta*	*S*	–C_6_H_5_	–CH_3_
**10a**	*meta*	*R*	–C_6_H_5_	–CH_3_
**1b**	*para*	*S*	–CH(CH_3_)_2_	–CH_3_
**2b**	*para*	*R*	–CH(CH_3_)_2_	–CH_3_
**3b**	*para*	*S*	–CH(CH_3_)_2_	–C_2_H_5_
**4b**	*para*	*S*	–CH(CH_3_)_2_	–C_4_H_9_
**5b**	*para*	*S*	–CH_2_CH(CH_3_)_2_	–CH_3_
**6b**	*para*	*R*	–CH_2_CH(CH_3_)_2_	–CH_3_
**7b**	*para*	*S*	–CH_2_CH(CH_3_)_2_	–C_2_H_5_
**8b**	*para*	*R*	–CH_2_CH(CH_3_)_2_	–C_2_H_5_
**9b**	*para*	*S*	–C_6_H_5_	–CH_3_
**10b**	*para*	*R*	–C_6_H_5_	–CH_3_

**Table 2:** Cap-specific stereochemistry and substitution.

Cap	*	X	Y
**1c**	*S*	-CH(CH_3_)_2_	Methyl
**2c**	*R*	-CH(CH_3_)_2_	Methyl
**3c**	*S*	-CH(CH_3_)_2_	Ethyl
**4c**	*S*	-CH(CH_3_)_2_	Butyl
**5c**	*S*	-CH_2_CH(CH_3_)_2_	Methyl
**6c**	*R*	-CH_2_CH(CH_3_)_2_	Methyl
**7c**	*S*	-CH_2_CH(CH_3_)_2_	Ethyl
**8c**	*R*	-CH_2_CH(CH_3_)_2_	Ethyl
**9c**	*S*	-C_6_H_5_	Methyl
**10c**	*R*	-C_6_H_5_	Ethyl

* = *R* or *S*.

**Table 3:** Activity, cytotoxicity, and selectivity of compound series **1a**–**10a** and **1b**–**10b** against genotype 1b (Con1) replicon ^a^.

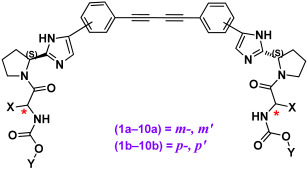						
Compound	Cap *	X	Y	HCV Replicon 1b		
				EC_50_ (nM) ^b^	CC_50_ (nM) ^b^	SI50
**1a**	*S*	–CH(CH_3_)_2_	–CH_3_	166.40	>100,000	>601.96
**2a**	*R*	–CH(CH_3_)_2_	–CH_3_	399.90	>100,000	>250.06
**3a**	*S*	–CH(CH_3_)_2_	–C_2_H_5_	158.80	>100,000	>629.72
**4a**	*S*	–CH(CH_3_)_2_	–C_4_H_9_	901.80	>100,000	>110.89
**5a**	*S*	–CH_2_CH(CH_3_)_2_	–CH_3_	113.70	>100,000	>879.51
**6a**	*R*	–CH_2_CH(CH_3_)_2_	–CH_3_	96.69	>100,000	>1034.23
**7a**	*S*	–CH_2_CH(CH_3_)_2_	–C_2_H_5_	97.89	>100,000	>1021.55
**8a**	*R*	–CH_2_CH(CH_3_)_2_	–C_2_H_5_	85.41	>100,000	>1170.82
**9a**	*S*	–C_6_H_6_	–CH_3_	62.04	>100,000	>1611.86
**10a**	*R*	–C_6_H_6_	–CH_3_	0.1001	>100,000	>999,001.00
**1b**	*S*	–CH(CH_3_)_2_	–CH_3_	89.80	>100,000	>1113.58
**2b**	*R*	–CH(CH_3_)_2_	–CH_3_	402.90	>100,000	>248.20
**3b**	*S*	–CH(CH_3_)_2_	–C_2_H_5_	336.20	>100,000	>297.44
**4b**	*S*	–CH(CH_3_)_2_	–C_4_H_9_	>1000	>100,000	ND
**5b**	*S*	–CH_2_CH(CH_3_)_2_	–CH_3_	16.12	>100,000	>6203.47
**6b**	*R*	–CH_2_CH(CH_3_)_2_	–CH_3_	42.11	>100,000	>1512.63
**7b**	*S*	–CH_2_CH(CH_3_)_2_	–C_2_H_5_	58.85	>100,000	>1699.23
**8b**	*R*	–CH_2_CH(CH_3_)_2_	–C_2_H_5_	77.52	>100,000	>1289.98
**9b**	*S*	–C_6_H_6_	–CH_3_	184.30	>100,000	>542.59
**10b**	*R*	–C_6_H_6_	–CH_3_	84.69	>100,000	>1180.77
Daclatasvir				0.027	17,700	655,556

^a^ Huh5-2 stable cell line containing the subgenomic reporter replicon of HCV 1b, Con1 strain; ^b^ Values are the mean of three independent experiments; standard deviation < 15%. * = *R* or *S*.

**Table 4:** Activity, cytotoxicity, and selectivity of compound **10a** in HCV genotype **2a** (JFH1), **3a** (S52) and **4a** (ED43) replicon assays, as well as HCV **1a** (H77) full length.

	Gt1a			Gt2a		Gt3a		Gt4a	
	CC_50_ (nM)	EC_50_ (nM)	SI	EC_50_ (nM)	SI	EC_50_ (nM)	SI	EC_50_ (nM)	SI
Comp. **10a**	>100,000	35.81	>2792.52	28.03	>3567.61	128.40	>777.00	65.04	>1537.52

## Data Availability

Data is contained within the article and in the Supplementary Materials.

## Supplementary Materials

The following supporting information can be downloaded at: https://www.mdpi.com/article/10.3390/ph15050632/s1, supplementary Tables S1, S2a and S2b; supplementary Figures S1–S21 and HRMS as well as NMR spectra.
