# Supplementary material for: Design and Synthesis of Novel Bis-Imidazolyl Phenyl Butadiyne Derivatives as HCV NS5A Inhibitors
Source: Pharmaceuticals (Basel). 2022 May 20;15(5):632. doi: 10.3390/ph15050632 (PMC9146377; doi:10.3390/ph15050632)

Supporting Information

Design and synthesis of novel bis-imidazolyl phenyl butadiyne derivatives as HCV NS5A inhibitors

Jehad Hamdy [1,#], Nouran Emadeldin [1,#], Mostafa M. Hamed [2], Efseveia Frakolaki [3,†], Sotirios Katsamakas[4], Niki Vassilaki [3], Grigoris Zoidis [4,*], Anna K. H. Hirsch [2,5], Mohammad Abdel-Halim [1,*] and Ashraf H. Abadi [1,*]

1 Department of Pharmaceutical Chemistry, Faculty of Pharmacy and Biotechnology, German University in Cairo, Cairo 11835, Egypt; jehad.hamdi93@gmail.com (J.H.); noura3110@gmail.com (N.E.)
2 Drug Design and Optimization, Helmholtz Institute for Pharmaceutical Research Saarland (HIPS)–Helmholtz Centre for Infection Research (HZI), Campus E8.1, 66123 Saarbrücken, Germany; mostafa.hamed@helmholtz-hips.de (M.M.H.); anna.hirsch@helmholtz-hips.de (A.K.H.H.)
3 Molecular Virology Laboratory, Department of Microbiology, Hellenic Pasteur Institute, Vas. Sofias Avenue, 11521 Athens, Greece;
sevif@pasteur.gr (E.F.); nikiv@pasteur.gr (N.V.)
4 Division of Pharmaceutical Chemistry, Department of Pharmacy, School of Health Sciences, National and Kapodistrian University of Athens, Panepistimiopolis-Zografou, 15771 Athens, Greece; sotikats@pharm.uoa.gr
5 Department of Pharmacy, Saarland University, Campus E8.1, 66123 Saarbrücken, Germany
* Correspondence: zoidis@pharm.uoa.gr (G.Z.); mohammad.abdel-halim@guc.edu.eg (M.A.-H.); ashraf.abadi@guc.edu.eg (A.H.A.); Tel.: +30-210-7274809 (G.Z.); +202-27590700 (ext. 3407) (M.A.-H.); +202-27590700 (ext. 3400) (A.H.A.)
† These authors contributed equally to this work.
‡ This manuscript is dedicated to the memory of our dear colleague and co-author Efseveia Frakolaki, who passed away while the manuscript was in preparation.

Supporting Information

Table of Content

Table S1. Molecular modeling results S3
Table S2a. Predicted drug-likeness properties S4
Table S2b. Predicted drug-likeness properties S5
Figure S1: Compound 1a docking solutions S6
Figure S2: Compound 2a docking solutions S7
Figure S3: Compound 3a docking solutions S8
Figure S4: Compound 4a docking solutions S9
Figure S5: Compound 5a docking solutions S10
Figure S6: Compound 6a docking solutions S11
Figure S7: Compound 7a docking solutions S12
Figure S8: Compound 8a docking solutions S13
Figure S9: Compound 9a docking solutions S14
Figure S10: Compound 1b docking solutions S15
Figure S11: Compound 2b docking solutions S16
Figure S12: Compound 3b docking solutions S17
Figure S13: Compound 4b docking solutions S18
Figure S14: Compound 5b docking solutions S19
Figure S15: Compound 6b docking solutions S20
Figure S16: Compound 7b docking solutions S21
Figure S17: Compound 8b docking solutions S22
Figure S18: Compound 9b docking solutions S23
Figure S19: Compound 10b docking solutions S24
Figure S20: OpenEye software collective docking solutions overlay S25
Figure S21: PyRx software collective docking solutions overlay S26
References S27
HRMS spectra and NMR spectra of **10a** S28

Table S1. Molecular modeling results

Comparative presentation of *in vitro* & *in silico* results of compounds versus HCV NS5A protein genotype 1b

a/a	Compound	NS5A EC_{50} (nM)	OE (docking score/Order)	PyRx (docking score/Order)
**1**	1a	166.40 / 15	-3.24 / 8	-8.30 / 5
**2**	2a	399.90 / 18	-4.69 / 2	-7.90 / 8
**3**	3a	158.80 / 14	-2.67 / 15	-7.90 / 8
**4**	4a	901.80 / 20	-1.56 / 18	-7.90 / 8
**5**	5a	113.70 / 13	-3.71 / 7	-7.90 / 8
**6**	6a	96.69 / 11	-2.72 / 14	-7.70 / 10
**7**	7a	97.89 / 12	-4.04 / 4	-8.20 / 6
**8**	8a	85.41 / 9	-1.73 / 17	-7.80 / 9
**9**	9a	62.04 / 5	-3.22 / 9	-9.40 / 1
**10**	10a	0.1001 / 2	-4.04 / 4	-8.80 / 2
**11**	1b	89.80 / 10	-2.96 / 10	-7.90 / 8
**12**	2b	402.90 / 19	-4.03 / 5	-7.70 / 10
**13**	3b	336.20 / 17	-2.76 / 13	-8.00 / 7
**14**	4b	>1000 / 21	-1.10 / 20	-7.60 / 11
**15**	5b	16.12 / 3	-2.91 / 11	-7.60 / 11
**16**	6b	66.11 / 6	-3.81 / 6	-7.80 / 9
**17**	7b	58.85 / 4	-2.49 / 16	-7.90 / 8
**18**	8b	77.52 / 7	-4.61 / 3	-7.20 / 12
**19**	9b	184.30 / 16	-1.40 / 19	-8.20 / 6
**20**	10b	84.69 / 8	-2.81 / 12	-8.40 / 4
**21**	Daclatasvir	0.0270 / 1	-5.89 / 1	-8.70 / 3

Table S2a. Predicted drug-likeness properties

	Compounds drug-likeness properties predicted with FAF4 online server [1]									
	**Compound 1a**	**Compound 2a**	**Compound 3a**	**Compound 4a**	**Compound 5a**	**Compound 6a**	**Compound 7a**	**Compound 8a**	**Compound 9a**	**Compound 10a**
**MW (<500Da)**	814.97	814.97	843.02	899.13	843.02	843.02	871.08	871.08	883.0	883.0
**logP (<5)**	5.75	5.75	6.48	8.25	6.46	6.46	7.19	7.19	6.01	6.01
**logD**	5.27	5.27	5.99	7.92	6.01	6.01	6.72	6.72	6.23	6.23
**logSw**	-7.73	-7.73	-8.23	-9.42	-8.22	-8.22	-8.71	-8.71	-8.39	-8.39
**tPSA (<140Å^2)**	174.64	174.64	174.64	174.64	174.64	174.64	174.64	174.64	174.64	174.64
**Fsp^3 (0-1)**	0.43	0.43	0.46	0.50	0.46	0.46	0.48	0.48	0.27	0.27
**HBD (<5)**	4	4	4	4	4	4	4	4	4	4
**HBA (<10)**	14	14	14	14	14	14	14	14	14	14
**Rotatable Bonds (<10)**	14	14	16	20	16	16	18	18	14	14
**Rigid Bonds**	42	42	42	42	42	42	42	42	54	54

Table S2b. Predicted drug-likeness properties

	Compounds drug-likeness properties predicted with FAF4 online server [1]										
	**Compound 1b**	**Compound 2b**	**Compound 3b**	**Compound 4b**	**Compound 5b**	**Compound 6b**	**Compound 7b**	**Compound 8b**	**Compound 9b**	**Compound 10b**	**Daclatasvir**
**MW (<500Da)**	814.97	814.97	843.02	899.13	843.02	843.02	871.08	871.08	883.0	883.0	738.87
**logP (<5)**	5.75	5.75	6.48	8.25	6.46	6.46	7.19	7.19	6.01	6.01	5.09
**logD**	5.27	5.27	5.99	7.92	6.01	6.01	6.72	6.72	6.23	6.23	4.16
**logSw**	-7.73	-7.73	-8.23	-9.42	-8.22	-8.22	-8.71	-8.71	-8.39	-8.39	-6.93
**tPSA (<140Å^2)**	174.64	174.64	174.64	174.64	174.64	174.64	174.64	174.64	174.64	174.64	174.64
**Fsp^3 (0-1)**	0.43	0.43	0.46	0.50	0.46	0.46	0.48	0.48	0.27	0.27	0.45
**HBD (<5)**	4	4	4	4	4	4	4	4	4	4	4
**HBA (<10)**	14	14	14	14	14	14	14	14	14	14	14
**Rotatable Bonds (<10)**	14	14	16	20	16	16	18	18	14	14	13
**Rigid Bonds**	42	42	42	42	42	42	42	42	54	54	40

Compound 1a docking solutions

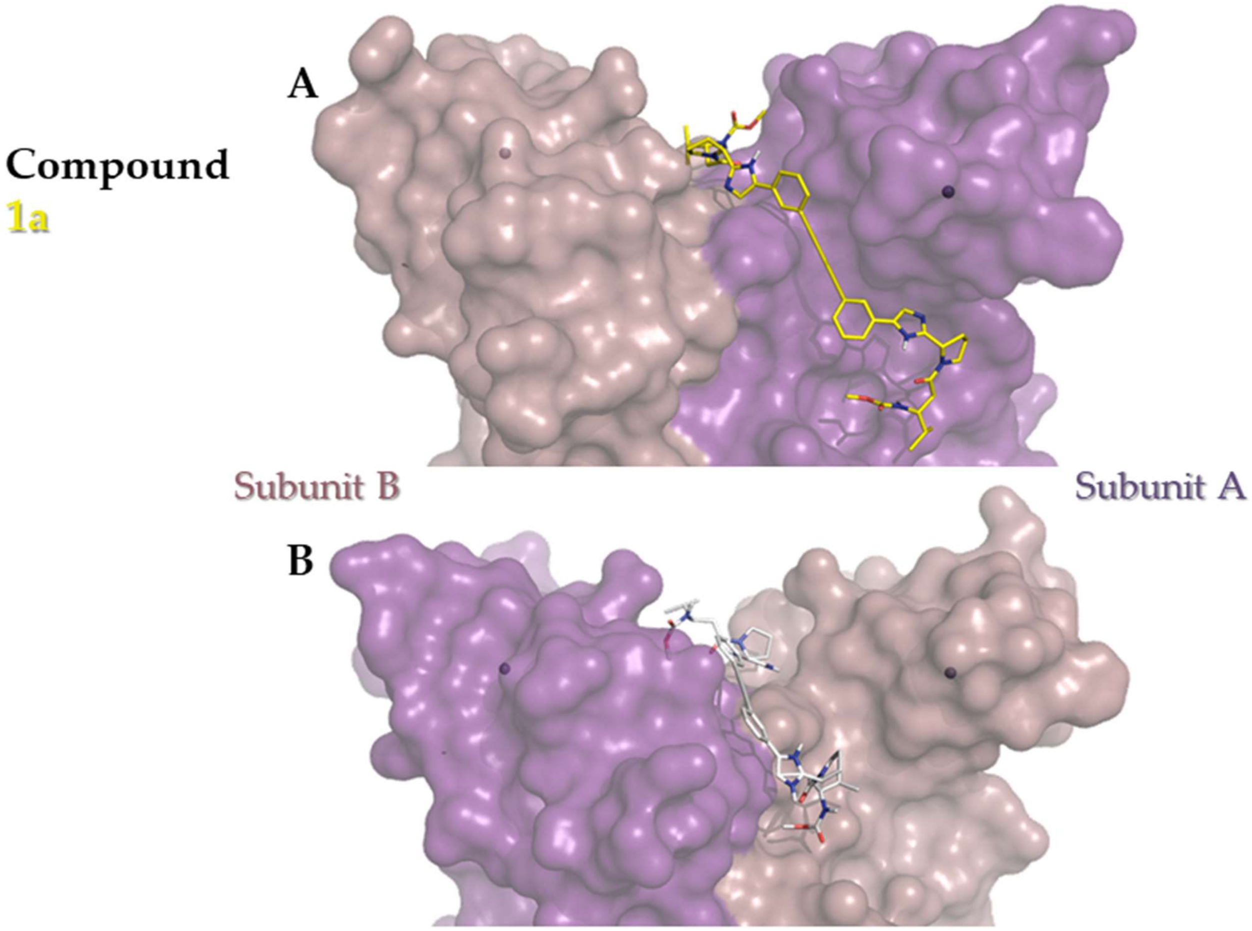

Figure S1. A) Purple and violet surface representation of the HCV GT 1b subunit A & subunit B respectively with the presence of **1a** (binding mode with the best score) obtained from the software OpenEye as yellow sticks and zinc metal shown as faded balls inside the surface, B) Purple and violet surface representation of the HCV GT 1b subunit A & subunit B respectively with the presence of **1a** (binding mode with the best score) obtained from the software PyRx as gray sticks and zinc metal shown as faded balls inside the surface.

Compound 2a docking solutions

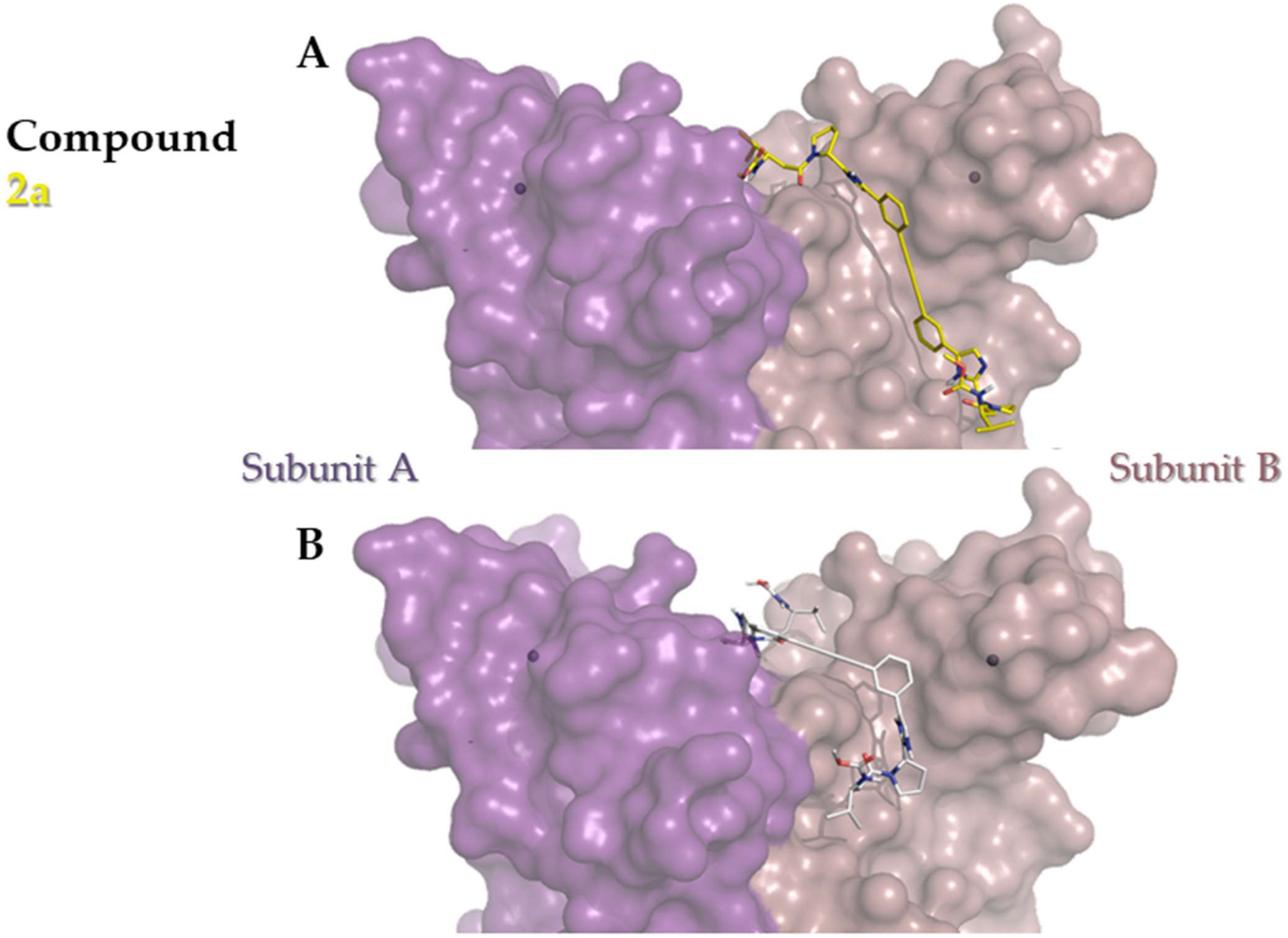

Figure S2. A) Purple and violet surface representation of the HCV GT 1b subunit A & subunit B respectively with the presence of **2a** (binding mode with the best score) obtained from the software OpenEye as yellow sticks and zinc metal shown as faded balls inside the surface, B) Purple and violet surface representation of the HCV GT 1b subunit A & subunit B respectively with the presence of **2a** (binding mode with the best score) obtained from the software PyRx as gray sticks and zinc metal shown as faded balls inside the surface.

Compound 3a docking solutions

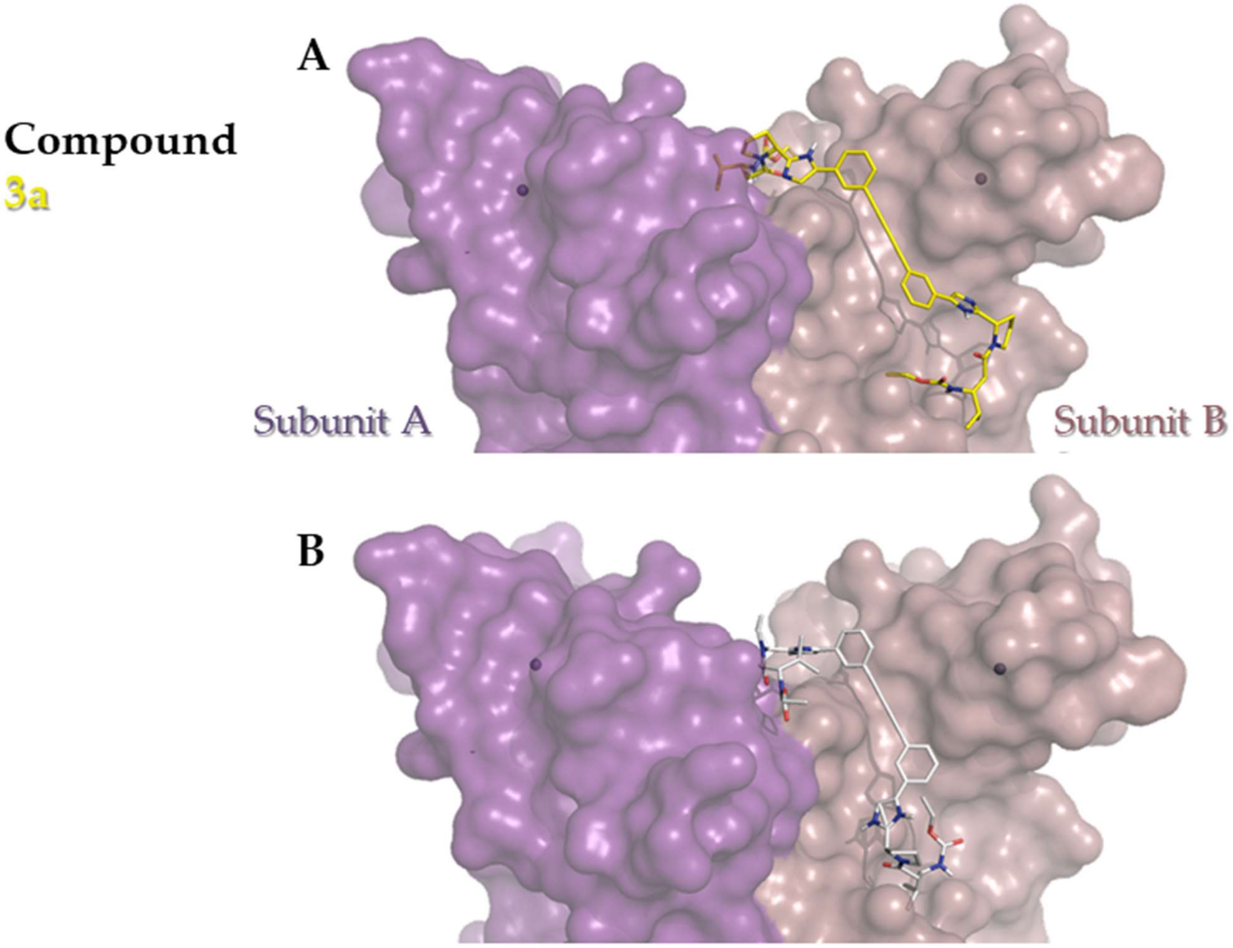

Figure S3. A) Purple and violet surface representation of the HCV GT 1b subunit A & subunit B respectively with the presence of **3a** (binding mode with the best score) obtained from the software OpenEye as yellow sticks and zinc metal shown as faded balls inside the surface, B) Purple and violet surface representation of the HCV GT 1b subunit A & subunit B respectively with the presence of **3a** (binding mode with the best score) obtained from the software PyRx as gray sticks and zinc metal shown as faded balls inside the surface.

Compound 4a docking solutions

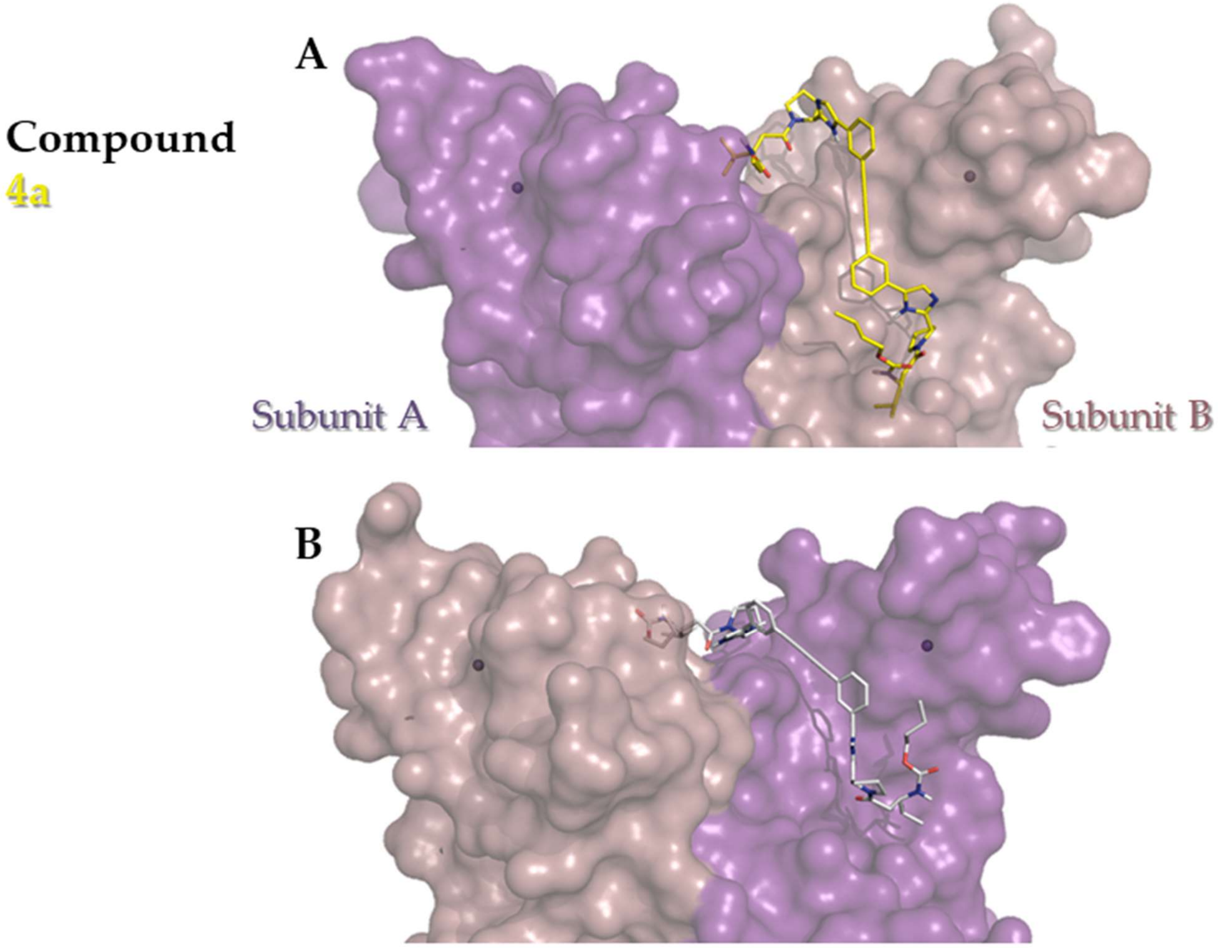

Figure S4. A) Purple and violet surface representation of the HCV GT 1b subunit A & subunit B respectively with the presence of **4a** (binding mode with the best score) obtained from the software OpenEye as yellow sticks and zinc metal shown as faded balls inside the surface, B) Purple and violet surface representation of the HCV GT 1b subunit A & subunit B respectively with the presence of **4a** (binding mode with the best score) obtained from the software PyRx as gray sticks and zinc metal shown as faded balls inside the surface.

Compound 5a docking solutions

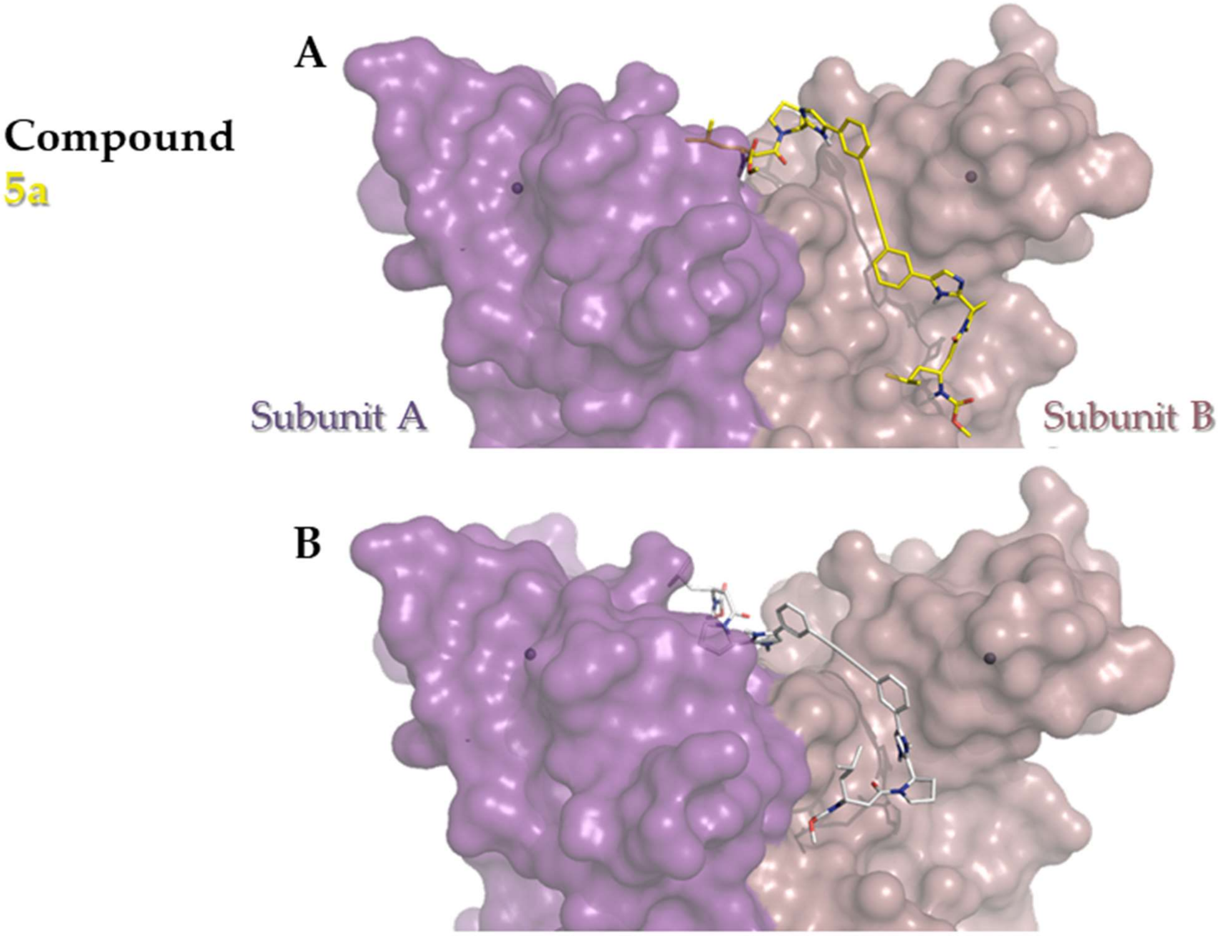

Figure S5. A) Purple and violet surface representation of the HCV GT 1b subunit A & subunit B respectively with the presence of **5a** (binding mode with the best score) obtained from the software OpenEye as yellow sticks and zinc metal shown as faded balls inside the surface, B) Purple and violet surface representation of the HCV GT 1b subunit A & subunit B respectively with the presence of **5a** (binding mode with the best score) obtained from the software PyRx as gray sticks and zinc metal shown as faded balls inside the surface.

Compound 6a docking solutions

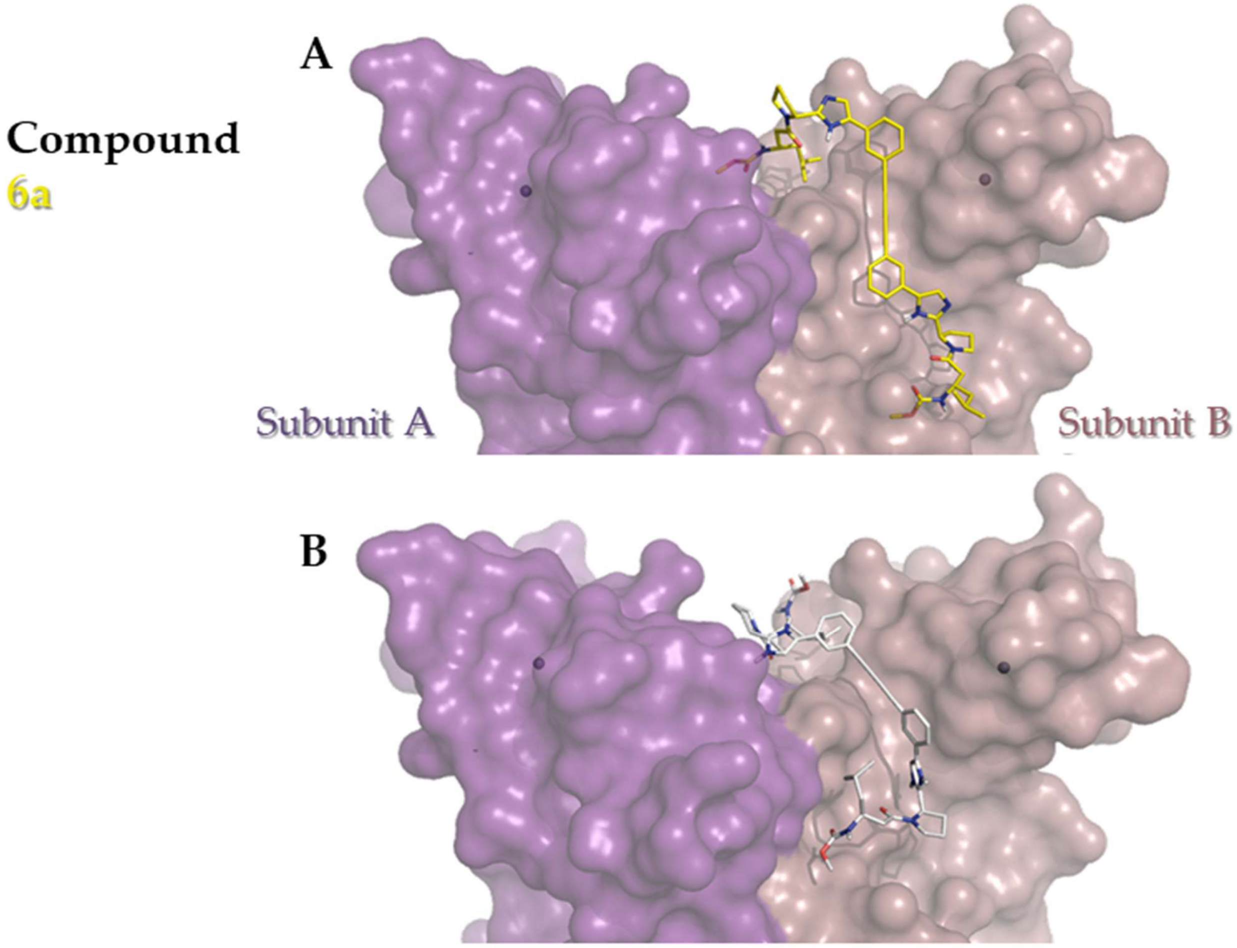

Figure S6. A) Purple and violet surface representation of the HCV GT 1b subunit A & subunit B respectively with the presence of **6a** (binding mode with the best score) obtained from the software OpenEye as yellow sticks and zinc metal shown as faded balls inside the surface, B) Purple and violet surface representation of the HCV GT 1b subunit A & subunit B respectively with the presence of **6a** (binding mode with the best score) obtained from the software PyRx as gray sticks and zinc metal shown as faded balls inside the surface.

Compound 7a docking solutions

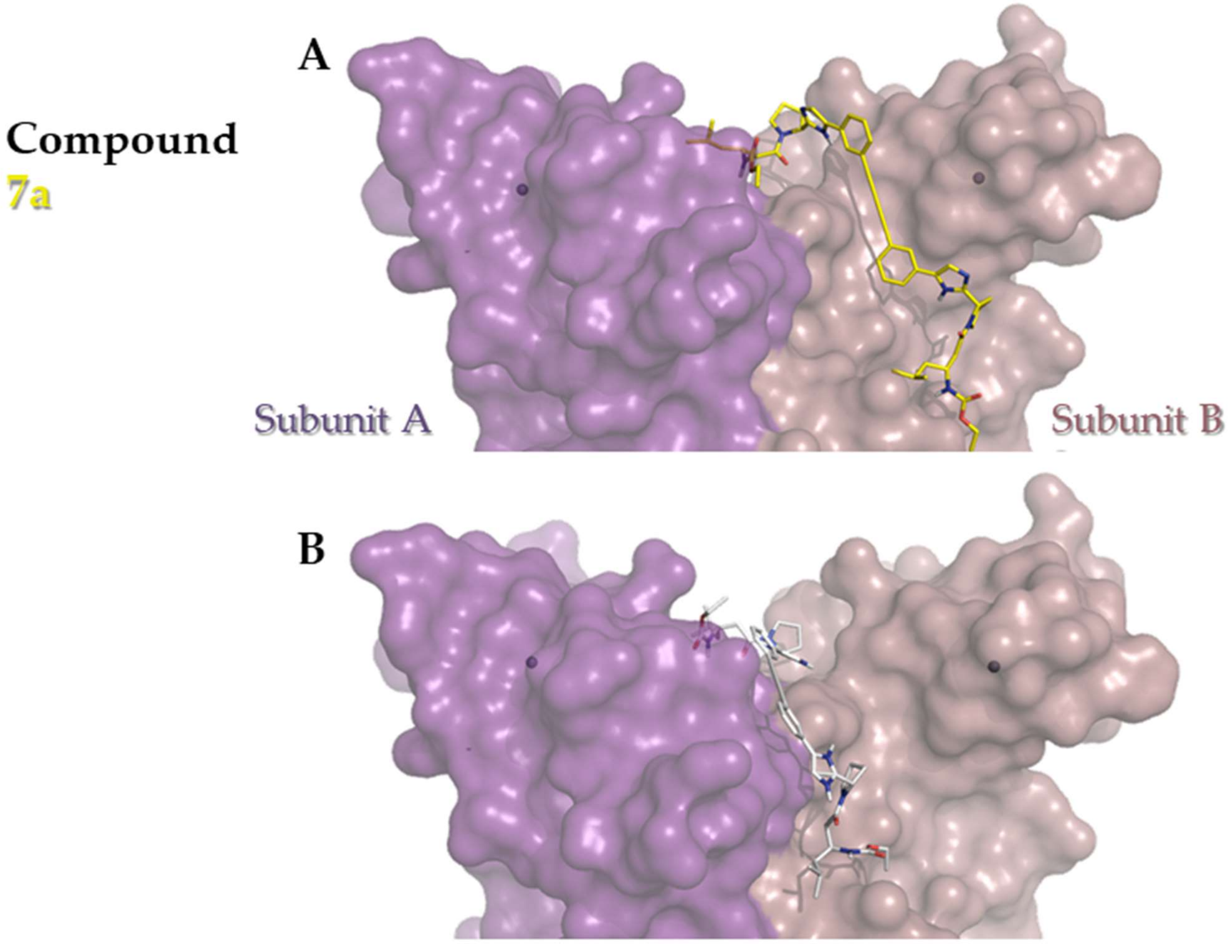

Figure S7. A) Purple and violet surface representation of the HCV GT 1b subunit A & subunit B respectively with the presence of **7a** (binding mode with the best score) obtained from the software OpenEye as yellow sticks and zinc metal shown as faded balls inside the surface, B) Purple and violet surface representation of the HCV GT 1b subunit A & subunit B respectively with the presence of **7a** (binding mode with the best score) obtained from the software PyRx as gray sticks and zinc metal shown as faded balls inside the surface.

Compound 8a docking solutions

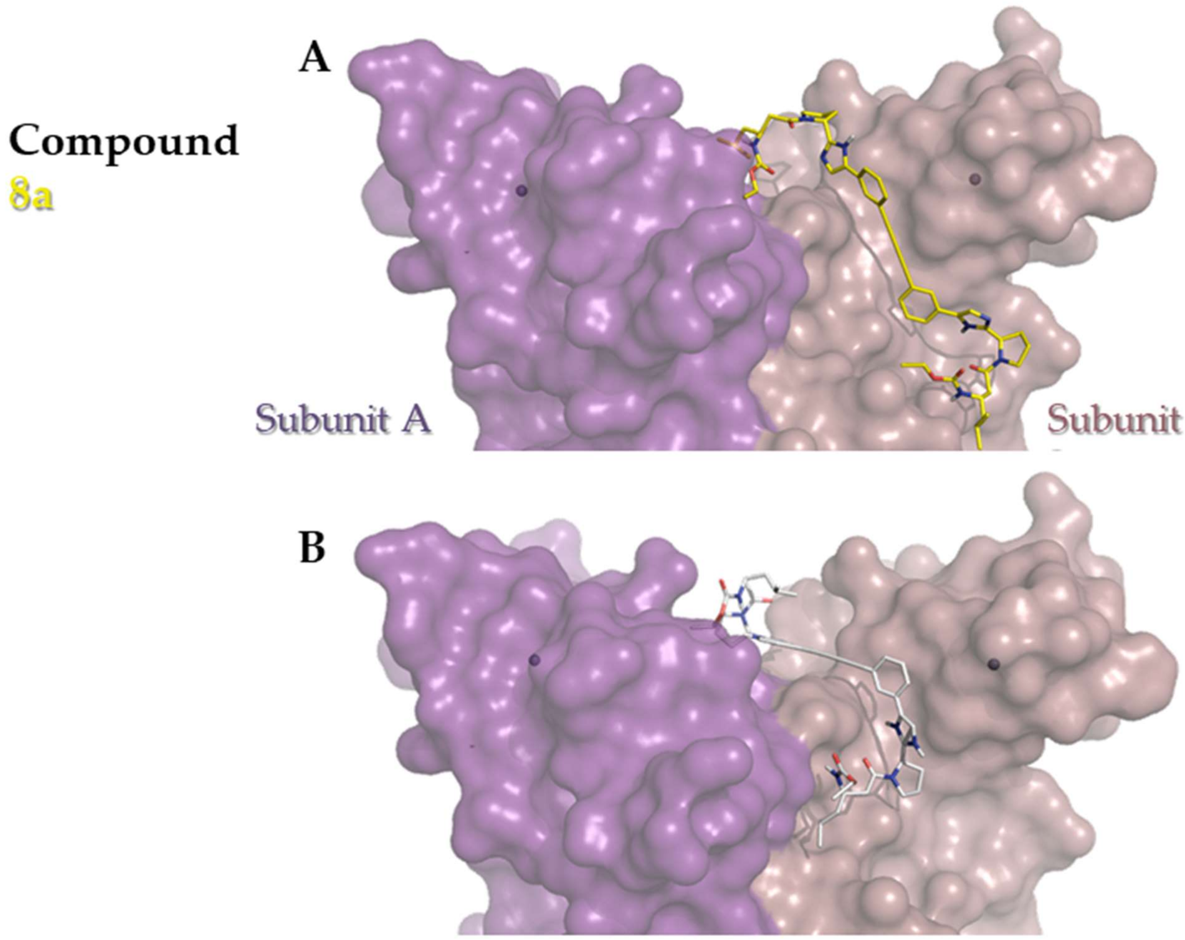

Figure S8. A) Purple and violet surface representation of the HCV GT 1b subunit A & subunit B respectively with the presence of **8a** (binding mode with the best score) obtained from the software OpenEye as yellow sticks and zinc metal shown as faded balls inside the surface, B) Purple and violet surface representation of the HCV GT 1b subunit A & subunit B respectively with the presence of **8a** (binding mode with the best score) binding mode obtained from the software PyRx as gray sticks and zinc metal shown as faded balls inside the surface.

Compound 9a docking solutions

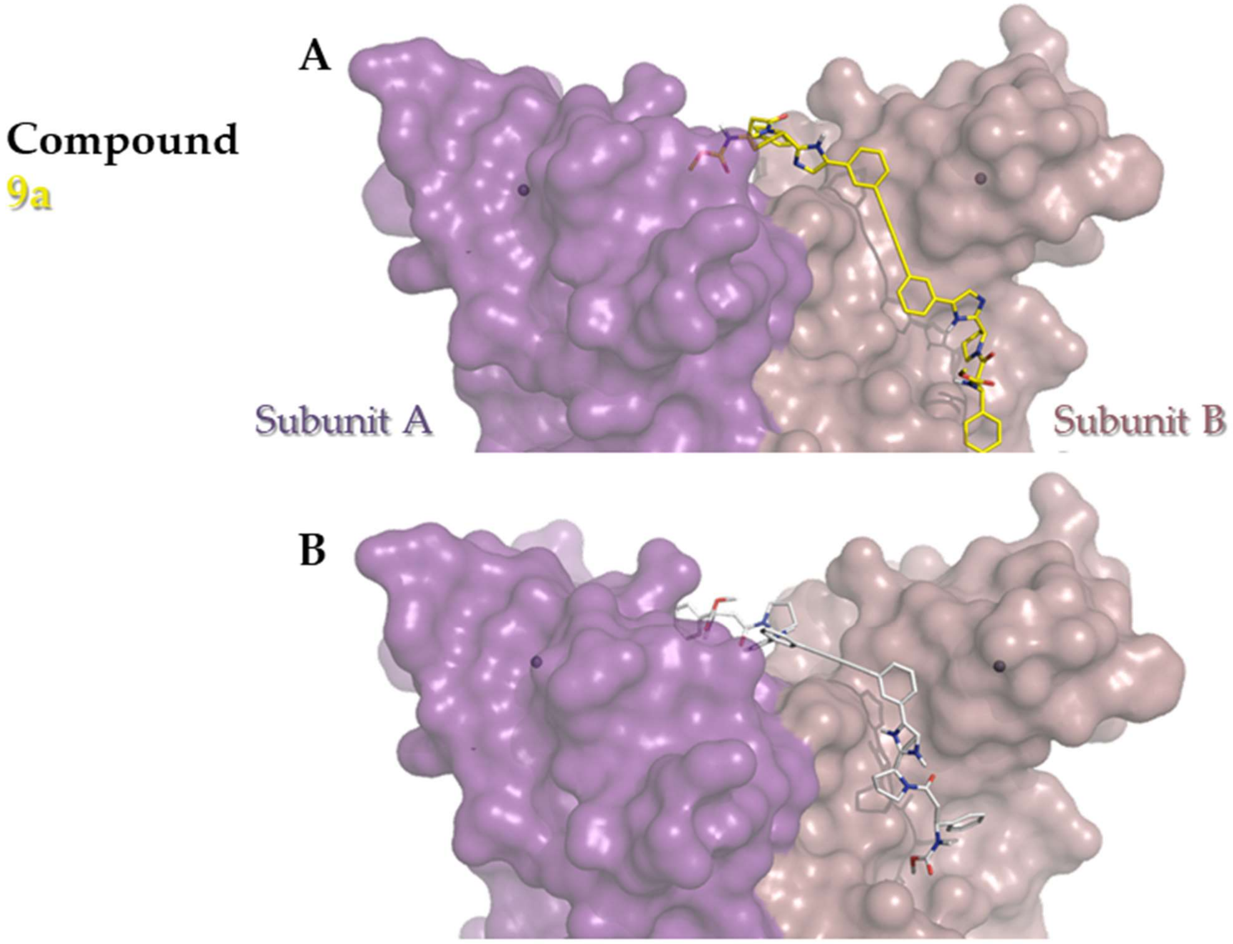

Figure S9. A) Purple and violet surface representation of the HCV GT 1b subunit A & subunit B respectively with the presence of **9a** (binding mode with the best score) obtained from the software OpenEye as yellow sticks and zinc metal shown as faded balls inside the surface, B) Purple and violet surface representation of the HCV GT 1b subunit A & subunit B respectively with the presence of **9a** (binding mode with the best score) obtained from the software PyRx as gray sticks and zinc metal shown as faded balls inside the surface.

Compound 1b docking solutions

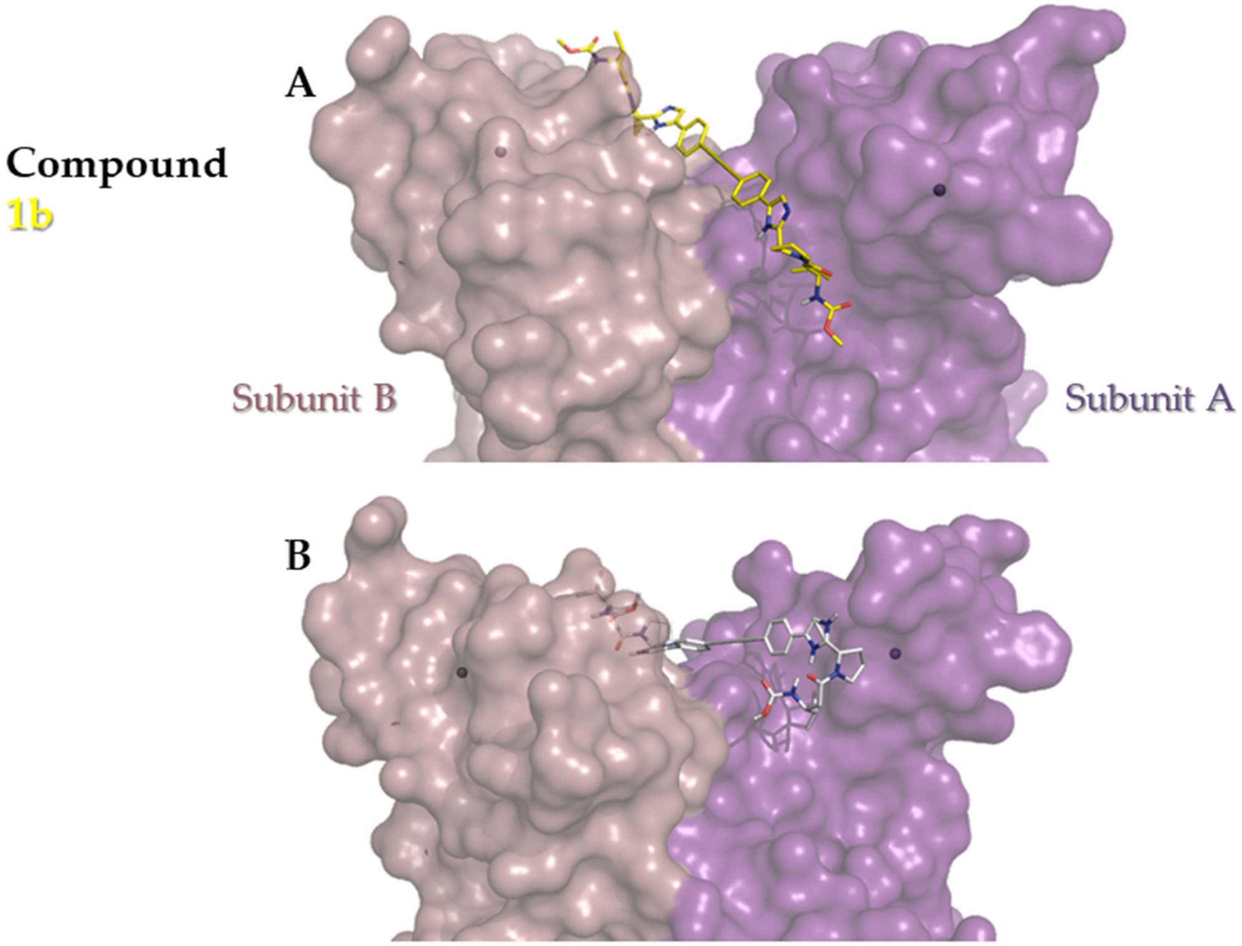

Figure S10. A) Purple and violet surface representation of the HCV GT 1b subunit A & subunit B respectively with the presence of **1b** (binding mode with the best score) obtained from the software OpenEye as yellow sticks and zinc metal shown as faded balls inside the surface, B) Purple and violet surface representation of the HCV GT 1b subunit A & subunit B respectively with the presence of **1b** (binding mode with the best score) obtained from the software PyRx as gray sticks and zinc metal shown as faded balls inside the surface.

Compound 2b docking solutions

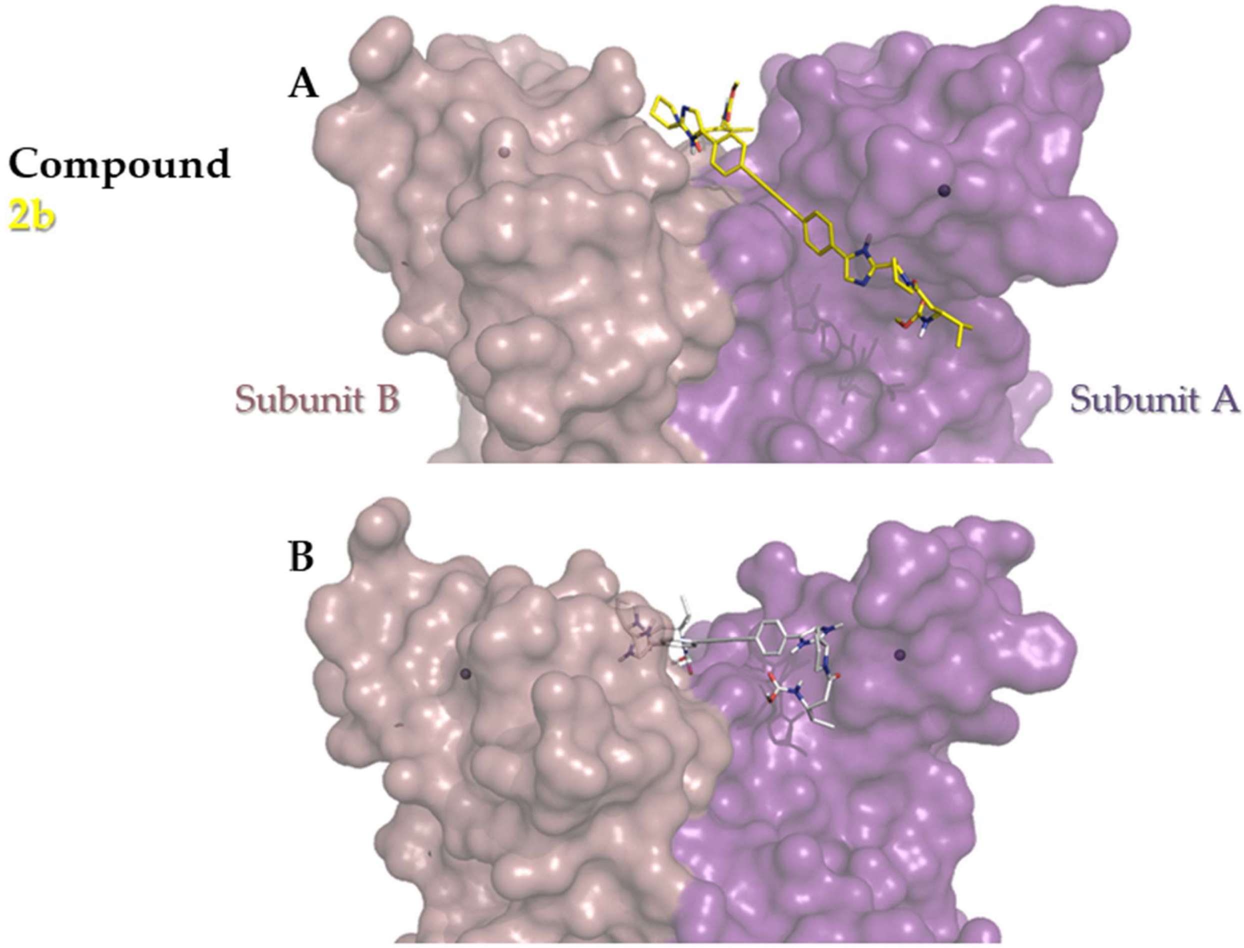

Figure S11. A) Purple and violet surface representation of the HCV GT 1b subunit A & subunit B respectively with the presence of **2b** (binding mode with the best score) obtained from the software OpenEye as yellow sticks and zinc metal shown as faded balls inside the surface, B) Purple and violet surface representation of the HCV GT 1b subunit A & subunit B respectively with the presence of **2b** (binding mode with the best score) obtained from the software PyRx as gray sticks and zinc metal shown as faded balls inside the surface.

Compound 3b docking solutions

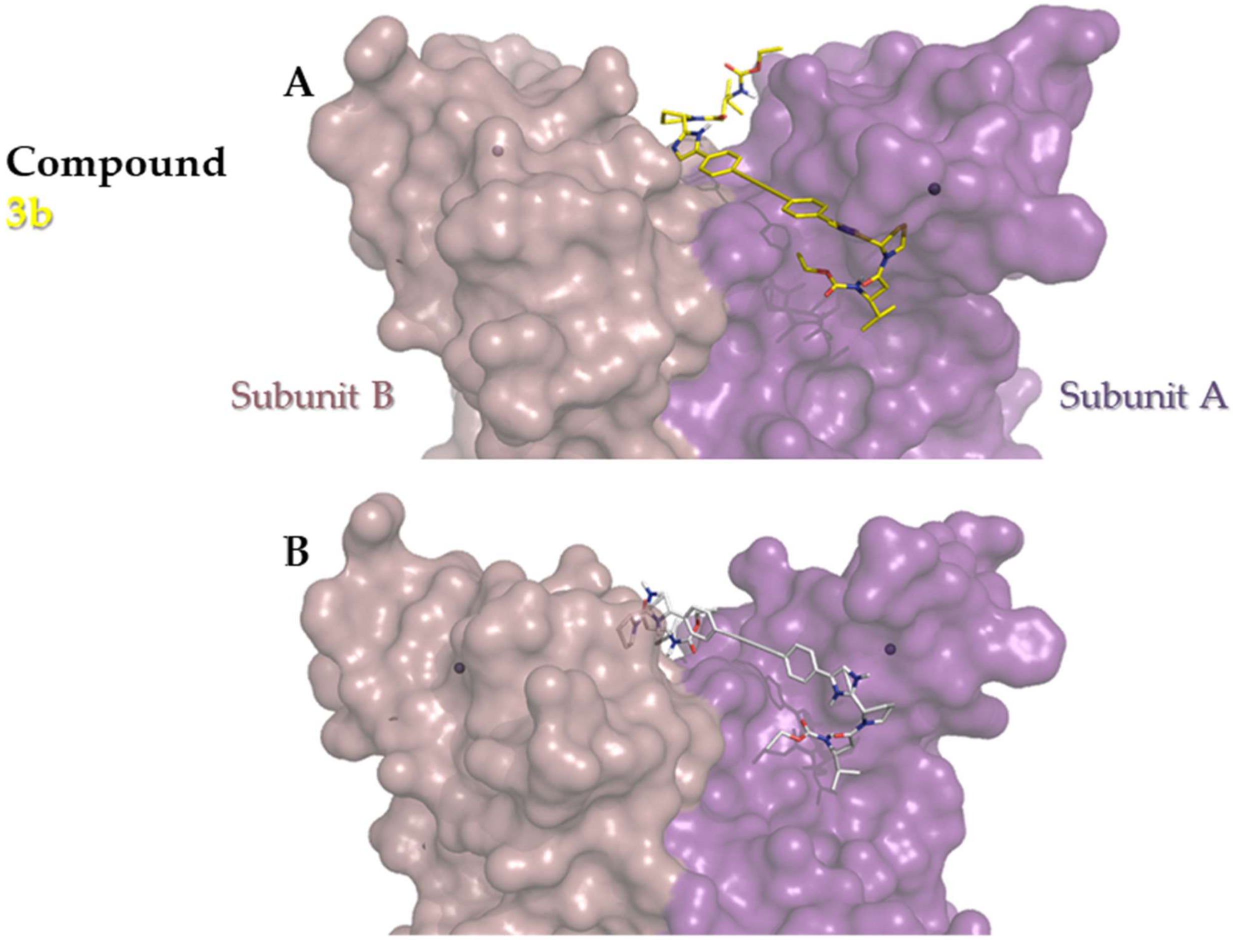

Figure S12. A) Purple and violet surface representation of the HCV GT 1b subunit A & subunit B respectively with the presence of **3b** (binding mode with the best score) obtained from the software OpenEye as yellow sticks and zinc metal shown as faded balls inside the surface, B) Purple and violet surface representation of the HCV GT 1b subunit A & subunit B respectively with the presence of **3b** (binding mode with the best score) obtained from the software PyRx as gray sticks and zinc metal shown as faded balls inside the surface.

Compound 4b docking solutions

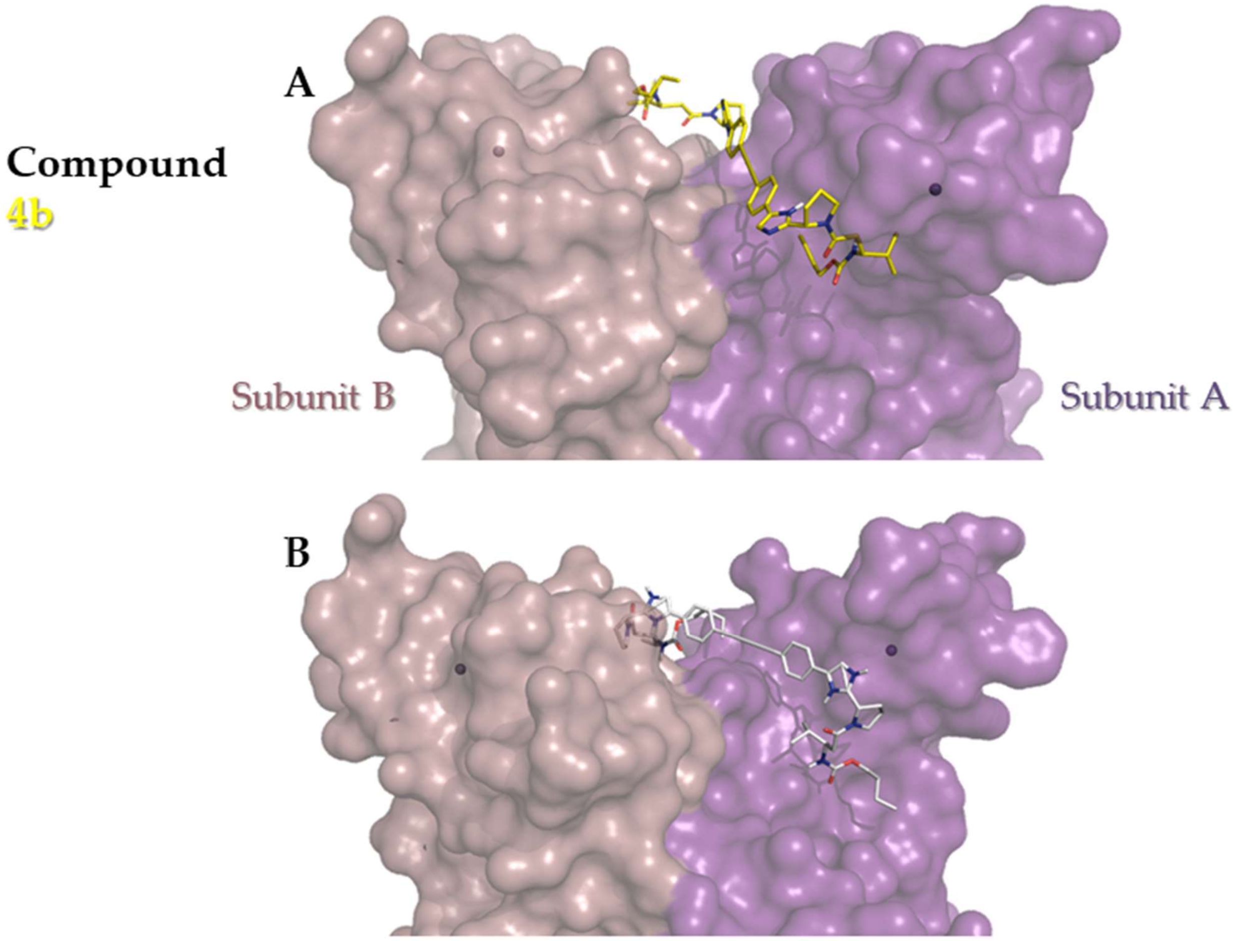

Figure S13. A) Purple and violet surface representation of the HCV GT 1b subunit A & subunit B respectively with the presence of **4b** (binding mode with the best score) obtained from the software OpenEye as yellow sticks and zinc metal shown as faded balls inside the surface, B) Purple and violet surface representation of the HCV GT 1b subunit A & subunit B respectively with the presence of **4b** (binding mode with the best score) obtained from the software PyRx as gray sticks and zinc metal shown as faded balls inside the surface.

Compound 5b docking solutions

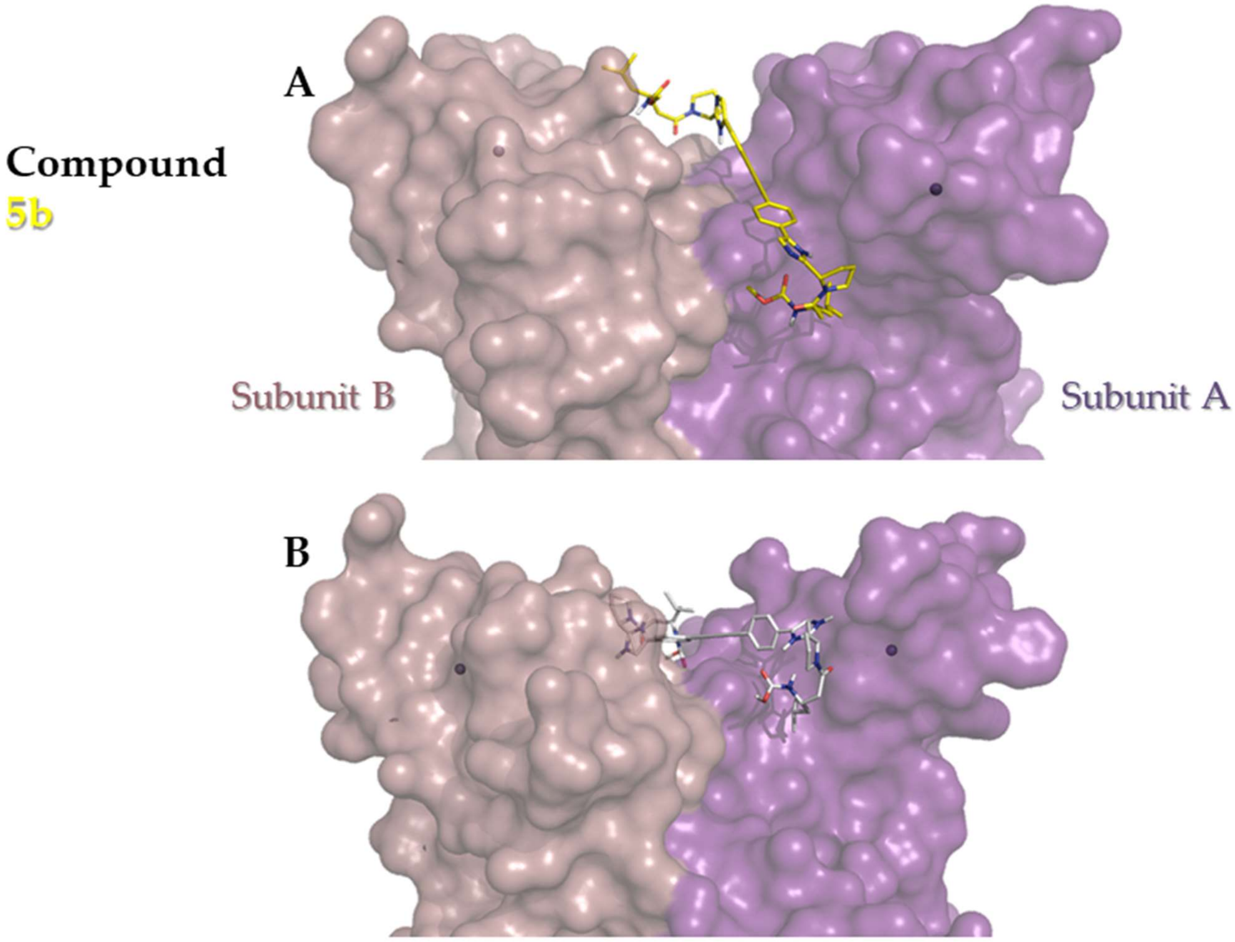

Figure S14. A) Purple and violet surface representation of the HCV GT 1b subunit A & subunit B respectively with the presence of **5b** (binding mode with the best score) obtained from the software OpenEye as yellow sticks and zinc metal shown as faded balls inside the surface, B) Purple and violet surface representation of the HCV GT 1b subunit A & subunit B respectively with the presence of **5b** (binding mode with the best score) obtained from the software PyRx as gray sticks and zinc metal shown as faded balls inside the surface.

Compound 6b docking solutions

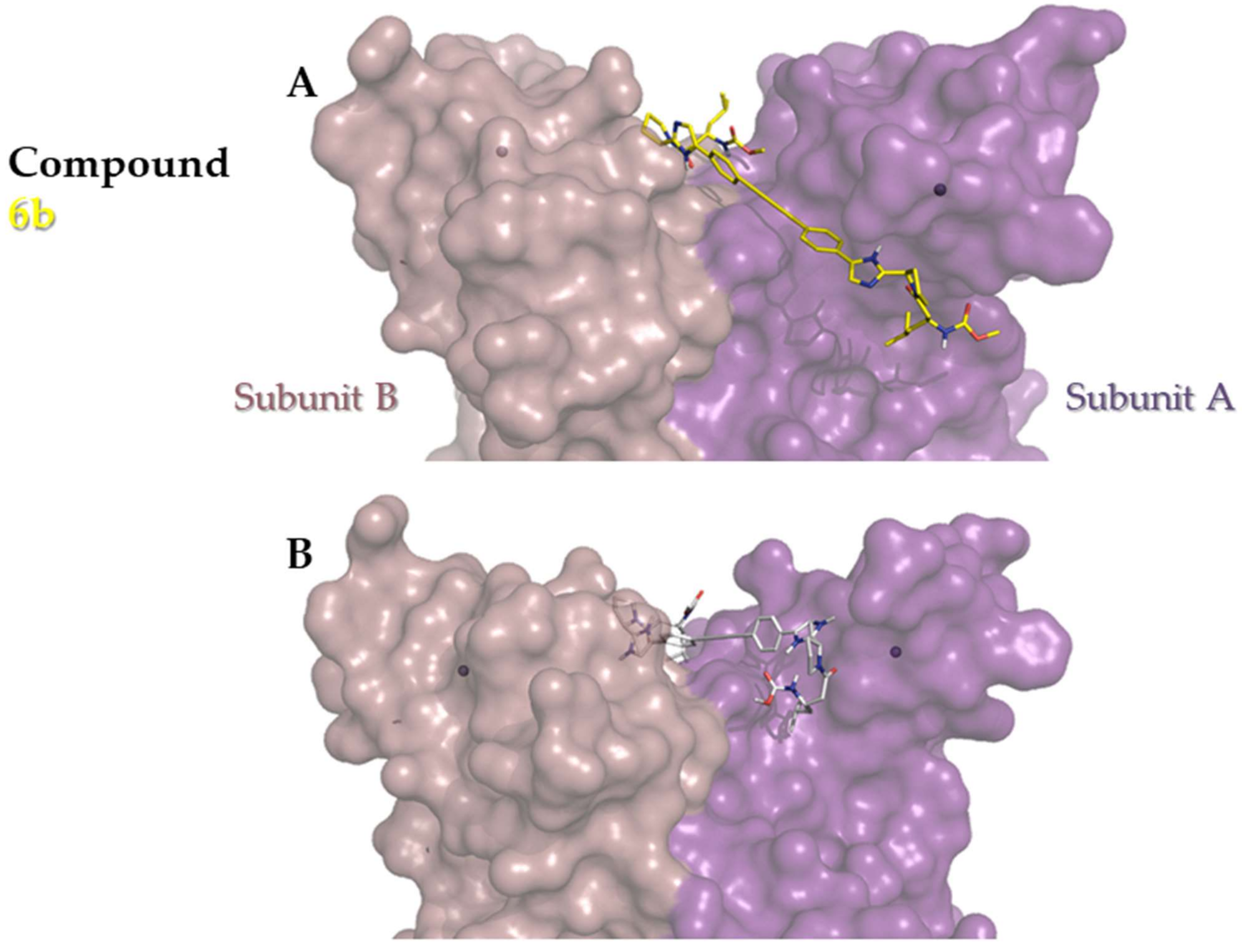

Figure S15. A) Purple and violet surface representation of the HCV GT 1b subunit A & subunit B respectively with the presence of **6b** (binding mode with the best score) obtained from the software OpenEye as yellow sticks and zinc metal shown as faded balls inside the surface, B) Purple and violet surface representation of the HCV GT 1b subunit A & subunit B respectively with the presence of **6b** (binding mode with the best score) obtained from the software PyRx as gray sticks and zinc metal shown as faded balls inside the surface.

Compound 7b docking solutions

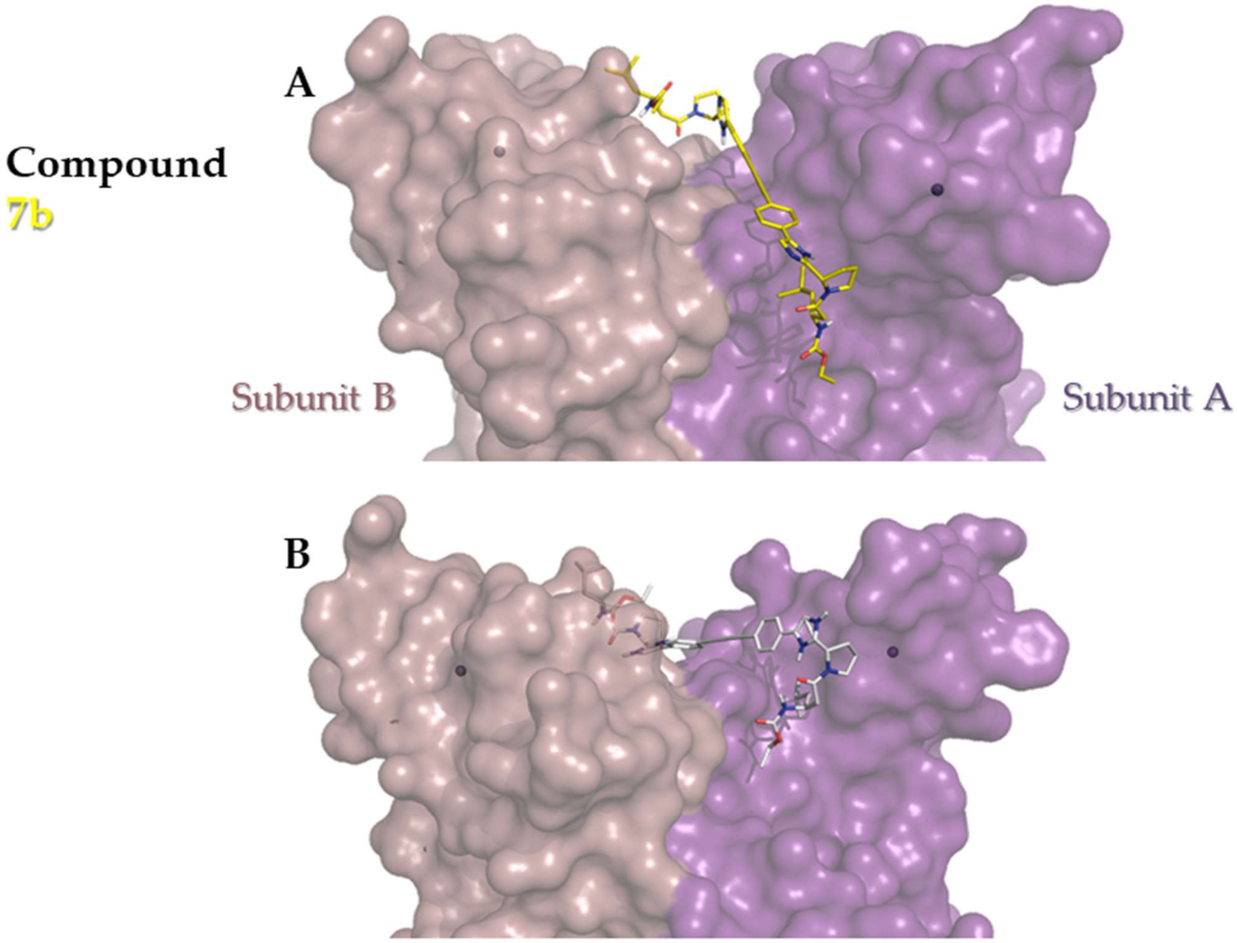

Figure S16. A) Purple and violet surface representation of the HCV GT 1b subunit A & subunit B respectively with the presence of **7b** (binding mode with the best score) obtained from the software OpenEye as yellow sticks and zinc metal shown as faded balls inside the surface, B) Purple and violet surface representation of the HCV GT 1b subunit A & subunit B respectively with the presence of **7b** (binding mode with the best score) obtained from the software PyRx as gray sticks and zinc metal shown as faded balls inside the surface.

Compound 8b docking solutions

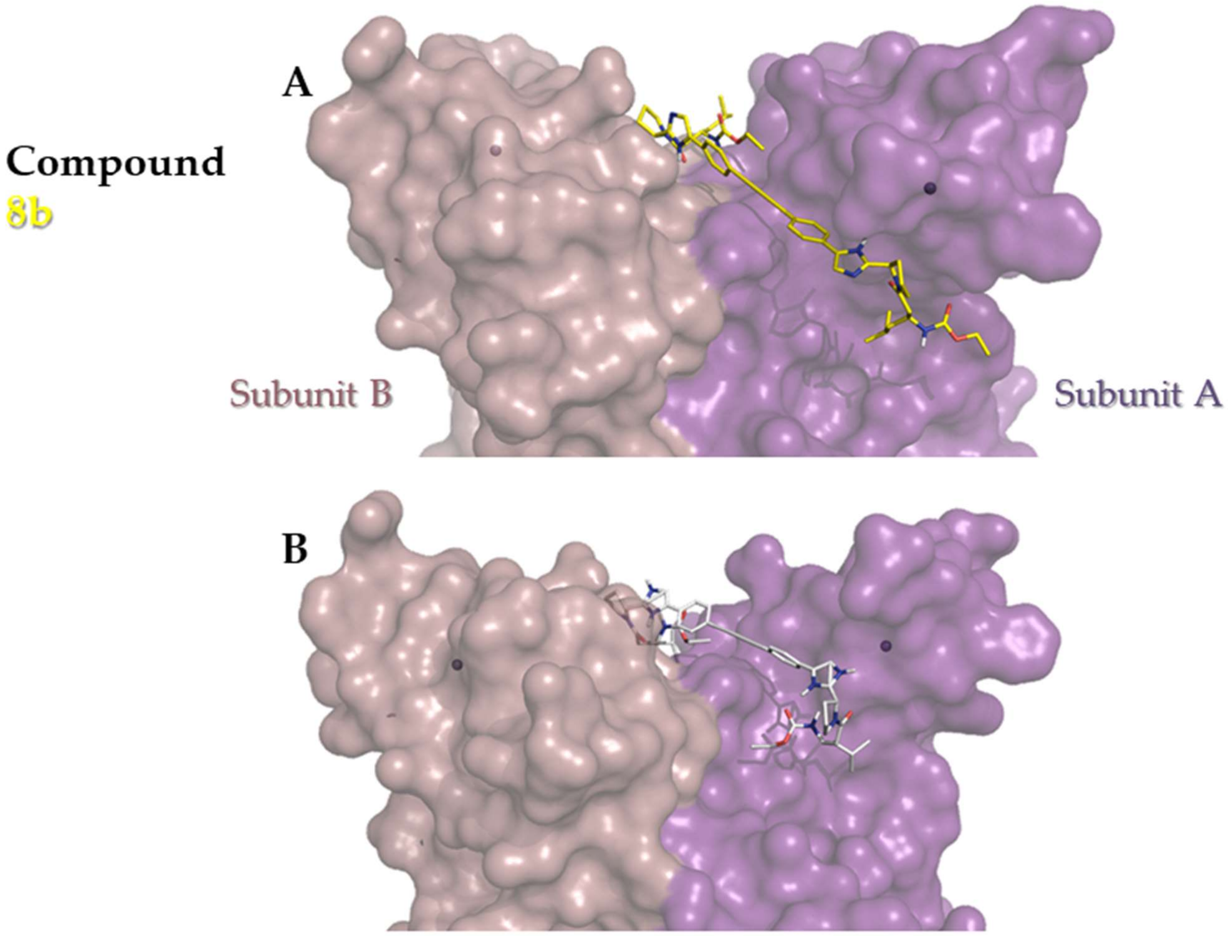

Figure S17. A) Purple and violet surface representation of the HCV GT 1b subunit A & subunit B respectively with the presence of **8b** (binding mode with the best score) obtained from the software OpenEye as yellow sticks and zinc metal shown as faded balls inside the surface, B) Purple and violet surface representation of the HCV GT 1b subunit A & subunit B respectively with the presence of **8b** (binding mode with the best score) obtained from the software PyRx as gray sticks and zinc metal shown as faded balls inside the surface.

Compound 9b docking solutions

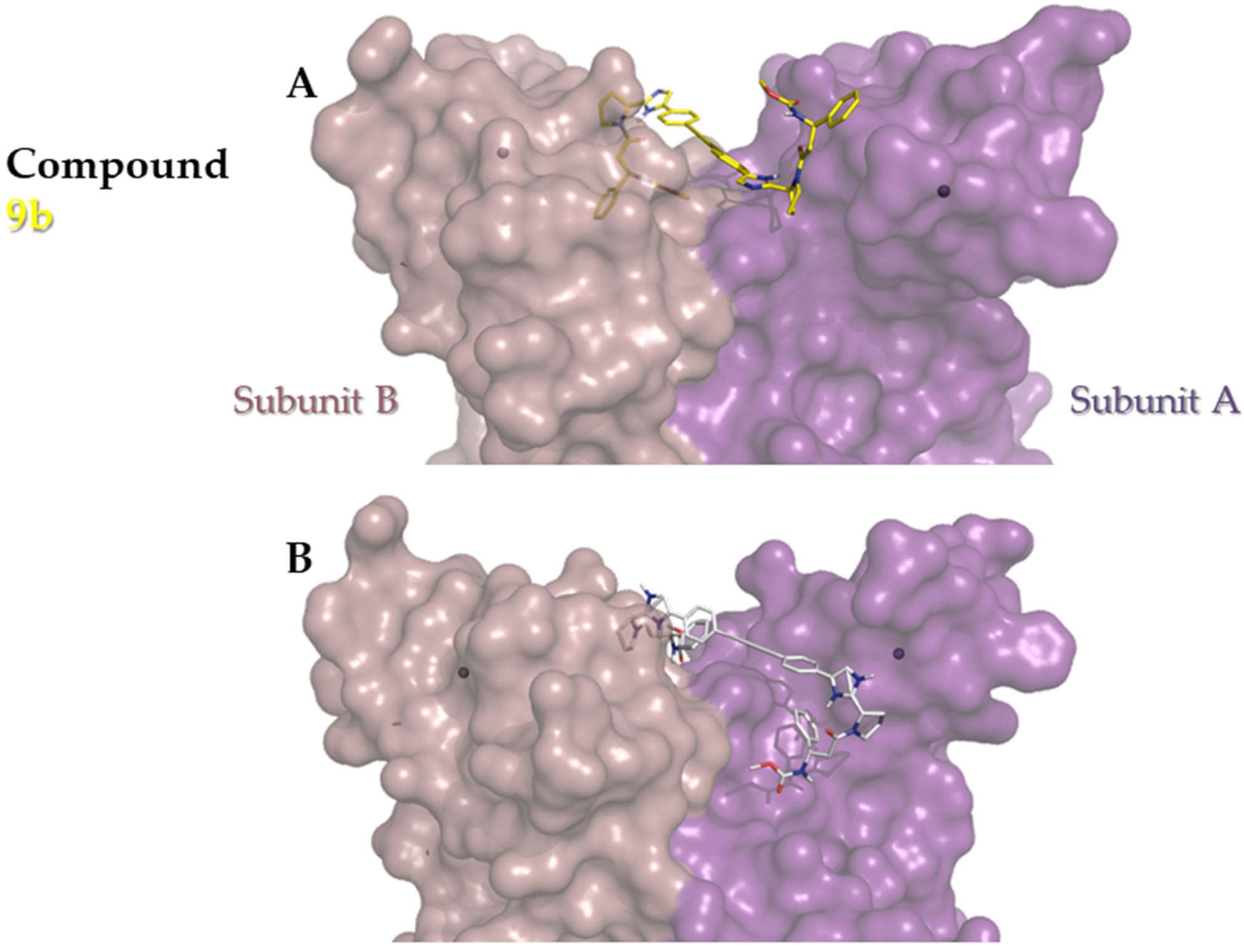

Figure S18. A) Purple and violet surface representation of the HCV GT 1b subunit A & subunit B respectively with the presence of **9b** (binding mode with the best score) obtained from the software OpenEye as yellow sticks and zinc metal shown as faded balls inside the surface, B) Purple and violet surface representation of the HCV GT 1b subunit A & subunit B respectively with the presence of **9b** (binding mode with the best score) obtained from the software PyRx as gray sticks and zinc metal shown as faded balls inside the surface.

Compound 10b docking solutions

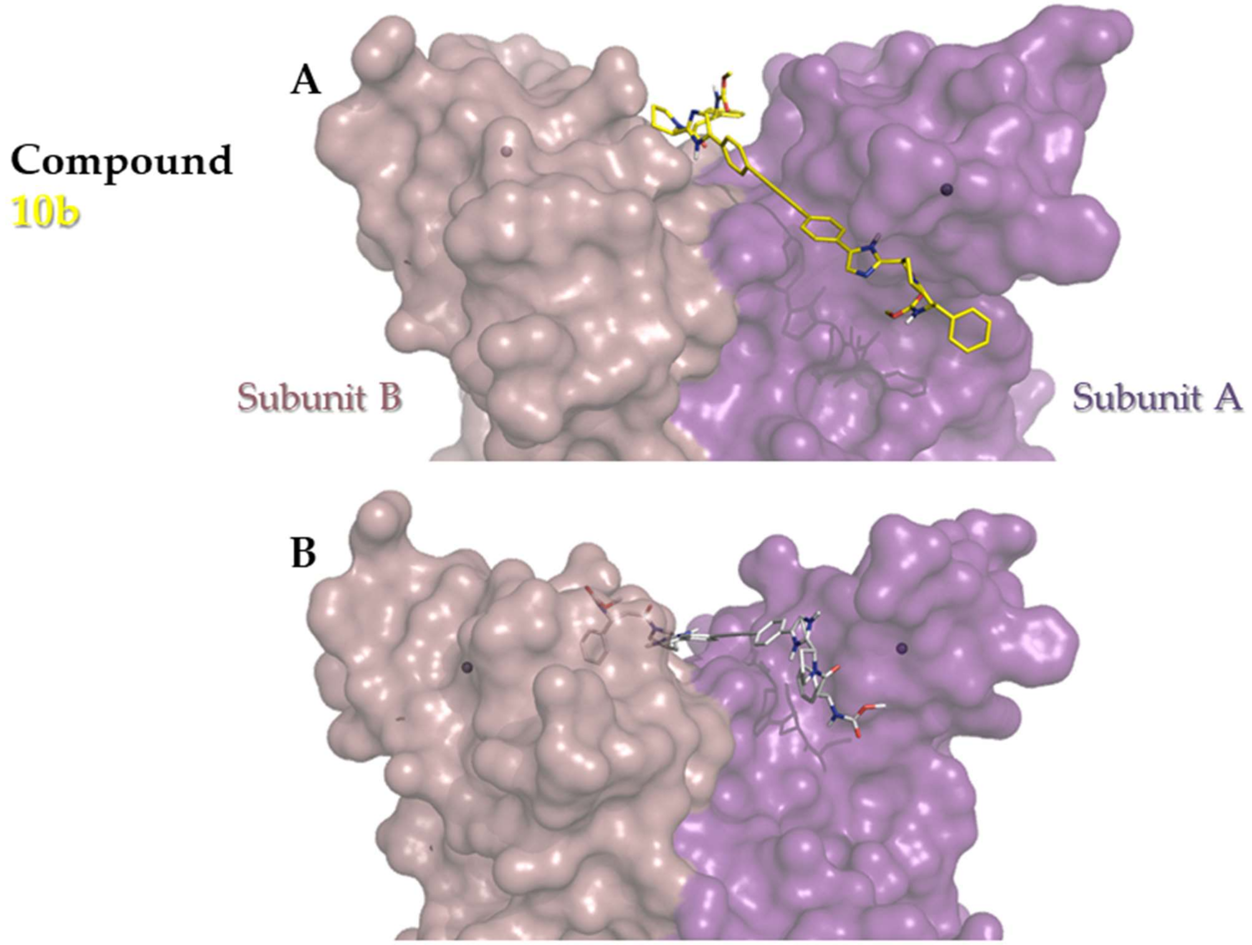

Figure S19. A) Purple and violet surface representation of the HCV GT 1b subunit A & subunit B respectively with the presence of **10b** (binding mode with the best score) obtained from the software OpenEye as yellow sticks and zinc metal shown as faded balls inside the surface, B) Purple and violet surface representation of the HCV GT 1b subunit A & subunit B respectively with the presence of **10b** (binding mode with the best score) obtained from the software PyRx as gray sticks and zinc metal shown as faded balls inside the surface.

OpenEye software collective docking solutions overlay

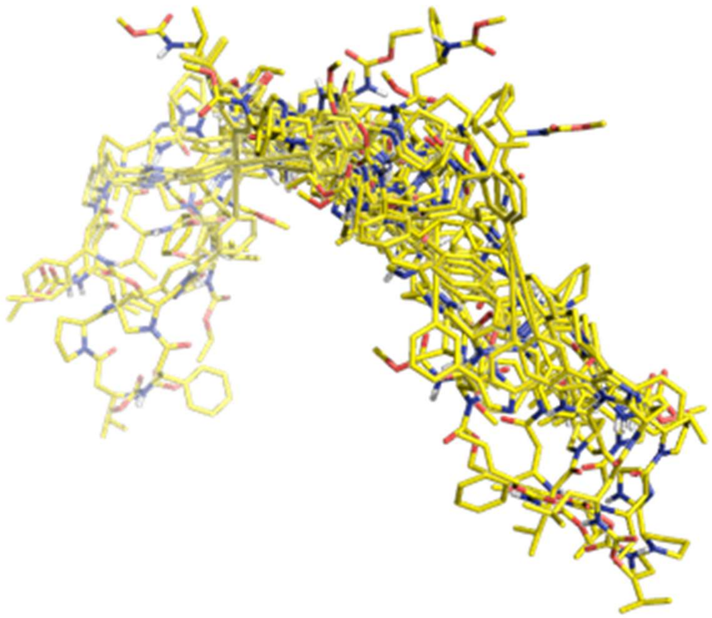

Figure S20. OpenEye software spatial distribution overlay representation of the solutions for **compounds 1a–10a** & **1b–10b** in yellow colored sticks at the absence of the protein.

PyRx software collective docking solutions overlay

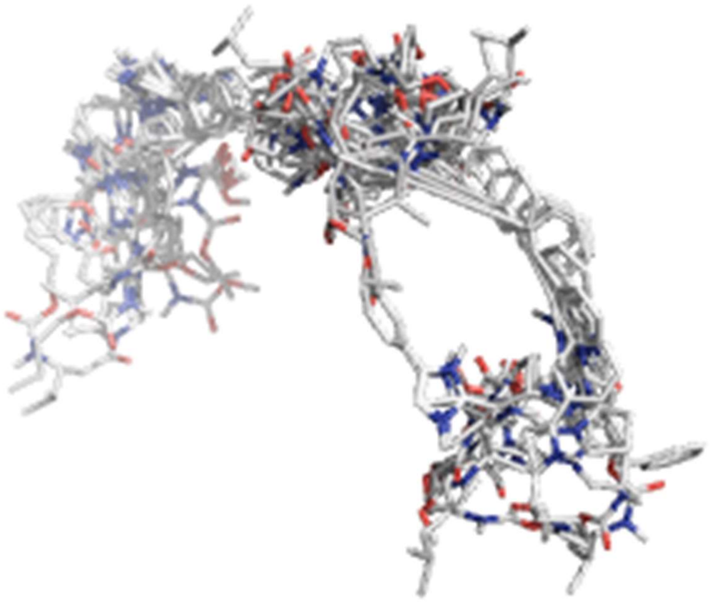

Figure S21. PyRx software spatial distribution overlay representation of the solutions for **compounds 1a-10a** & **1b-10b** in gray colored sticks at the absence of the protein.

References

1. Lagorce, D.; Bouslama, L.; Becot, J.; Miteva, M.A.; Villoutreix, B.O. FAF-Drugs4: free ADME-tox filtering computations for chemical biology and early stages drug discovery. *Bioinformatics* **2017**, *33*, 3658-3660, doi:10.1093/bioinformatics/btx491.

HRMS and NMR spectra

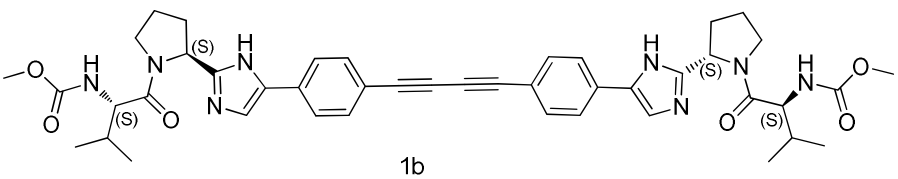

1b

HRMS

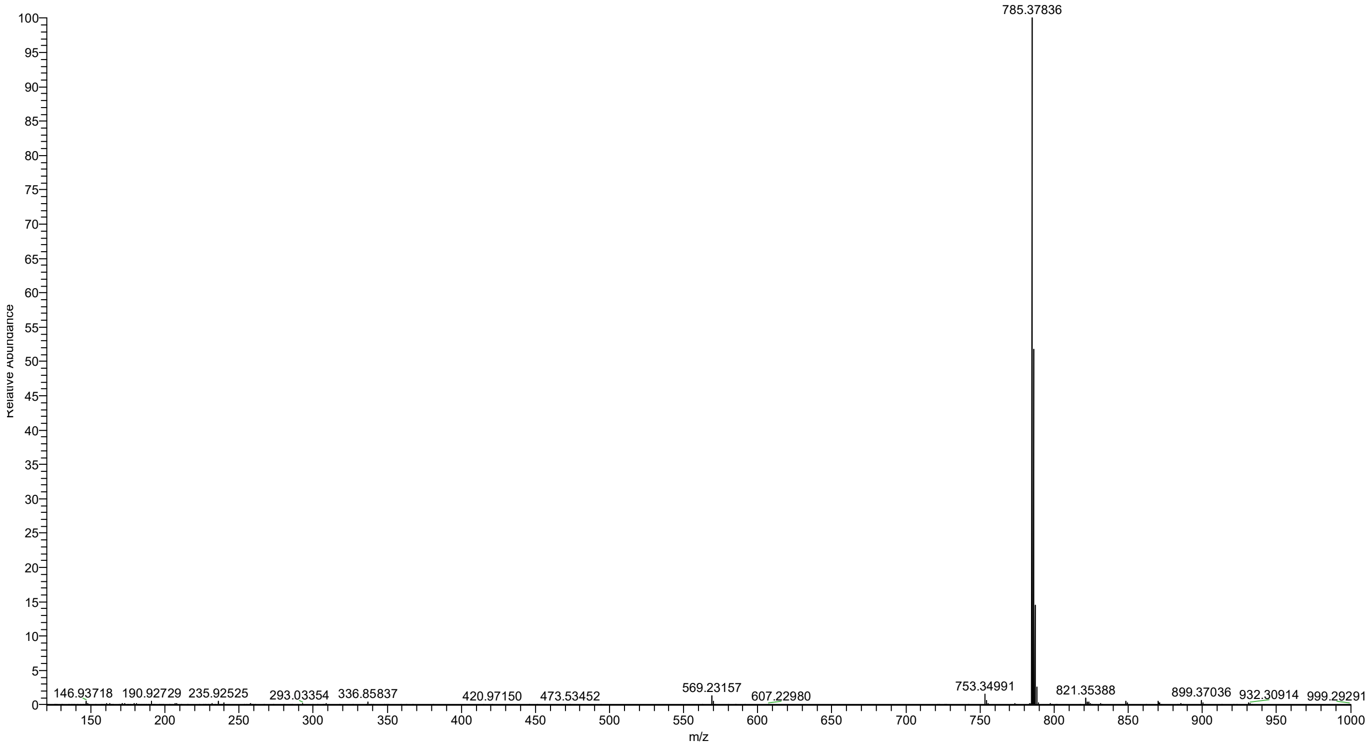

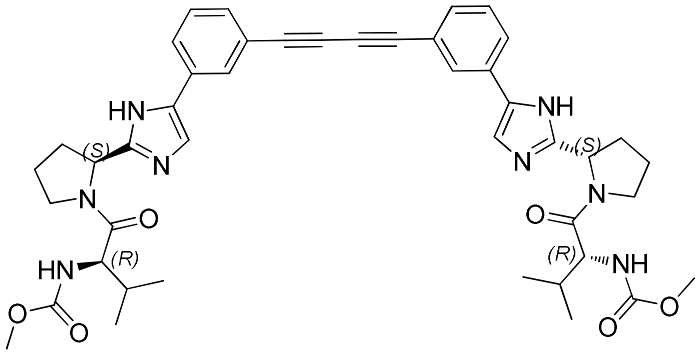
HN
N
(S)
N
O
HN
(R)
O
O
NH
N
(S)
N
O
(R)
NH
O
O

2a

HRMS

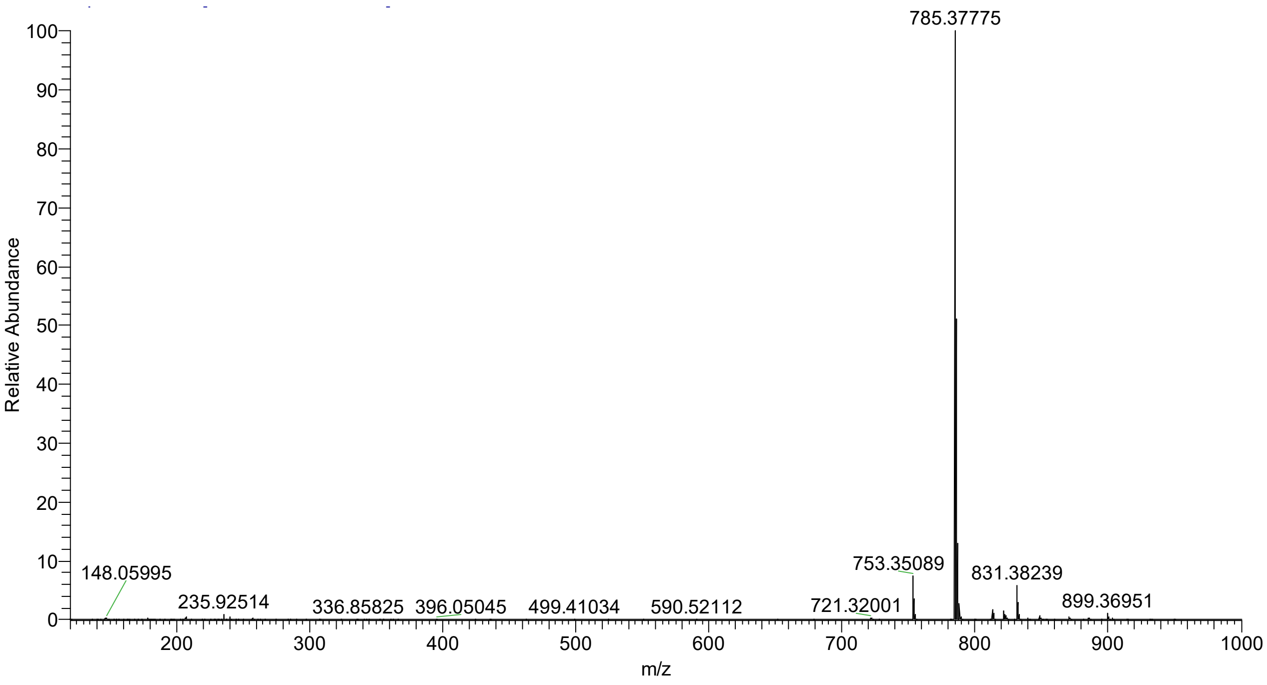
785.37775
Relative Abundance
148.05995
235.92514
336.85825
396.05045
499.41034
590.52112
721.32001
753.35089
831.38239
899.36951
m/z

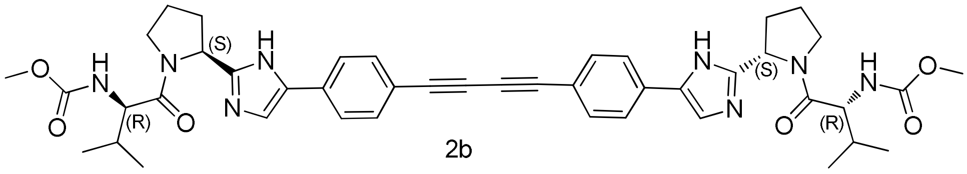

O
H
N
(R)
O
N
(S)
H
N
N
H
N
(S)
N
O
(R)
H
N
O

2b

HRMS

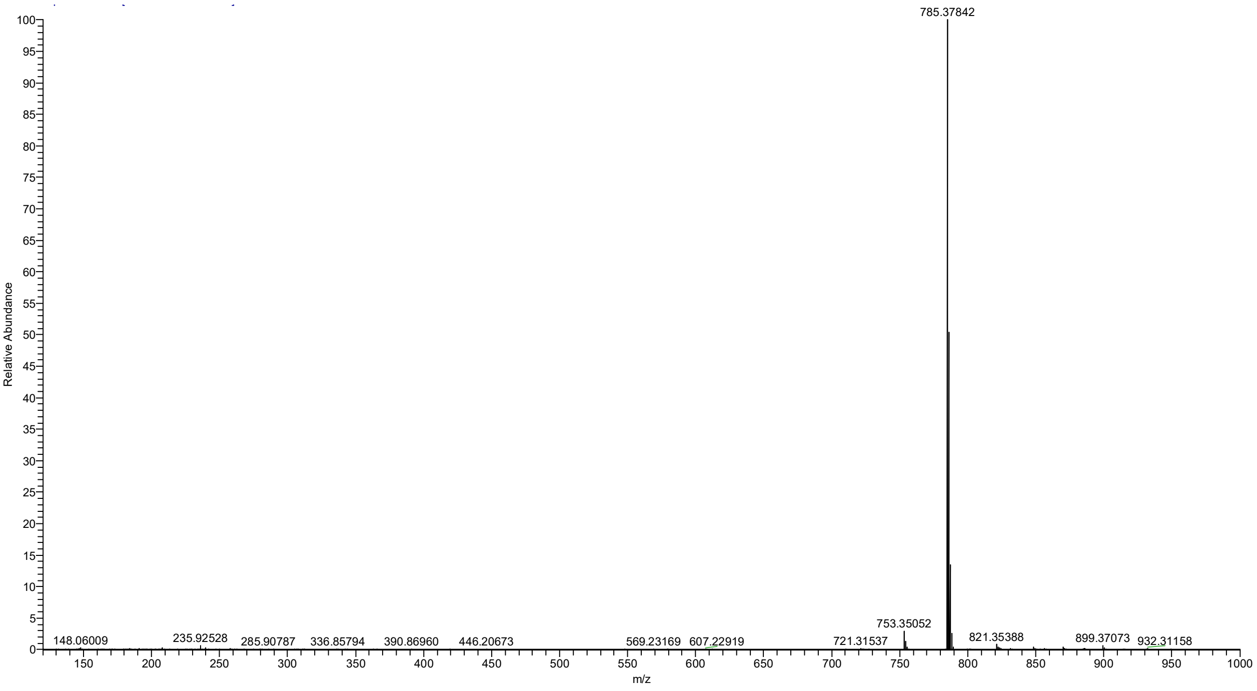

785.37842
Relative Abundance
148.06009
235.92528
285.90787
336.85794
390.86960
446.20673
569.23169
607.22919
721.31537
753.35052
821.35388
899.37073
932.31158
m/z

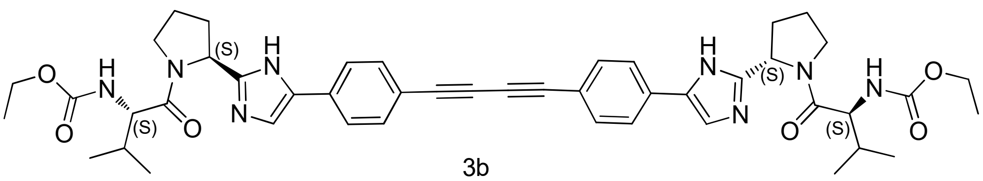

O
H
N
N
(S)
H
N
N
O
O
(S)

3b

HRMS

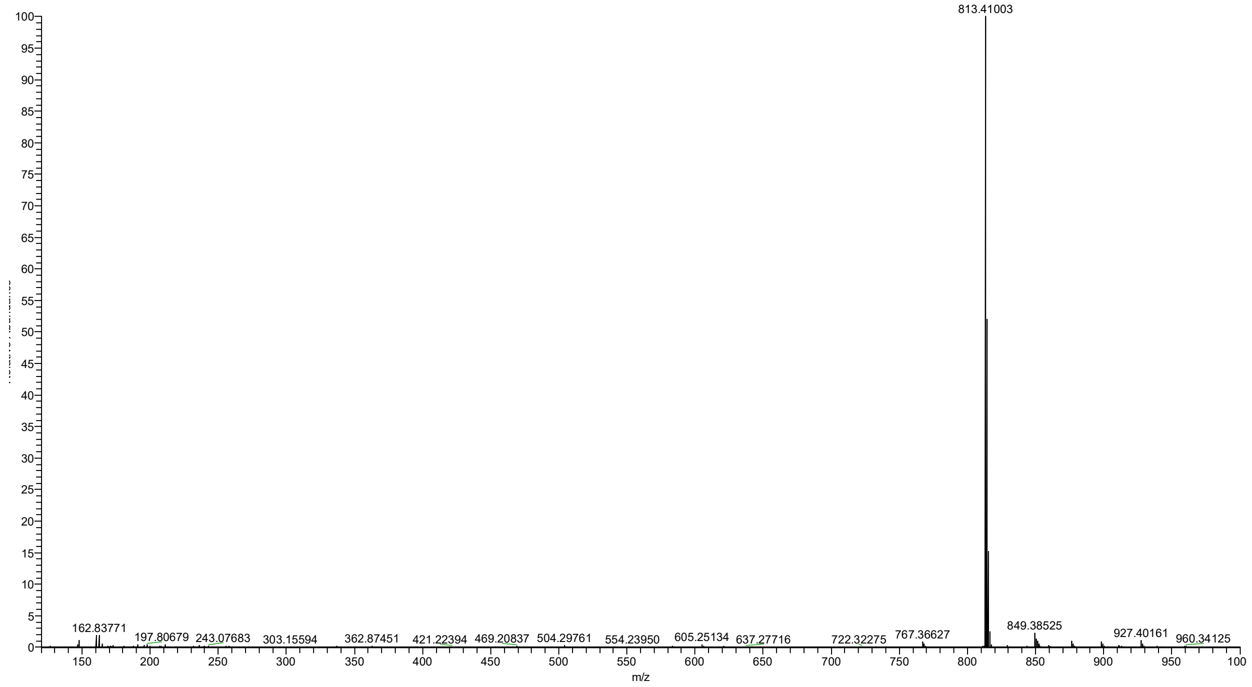

813.41003
162.83771
197.80679
243.07683
303.15594
362.87451
421.22394
469.20837
504.29761
554.23950
605.25134
637.27716
722.32275
767.36627
849.38525
927.40161
960.34125
m/z

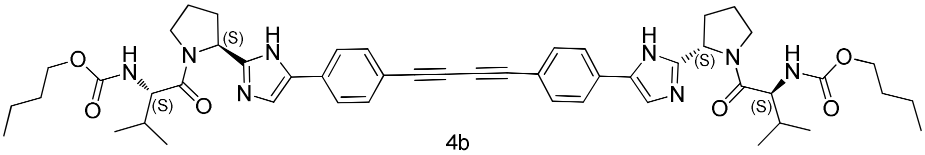

O
H
N
O
(S)
N
O
(S)
H
N
N
H
N
N
(S)
N
O
H
N
(S)
O
O

4b

HRMS

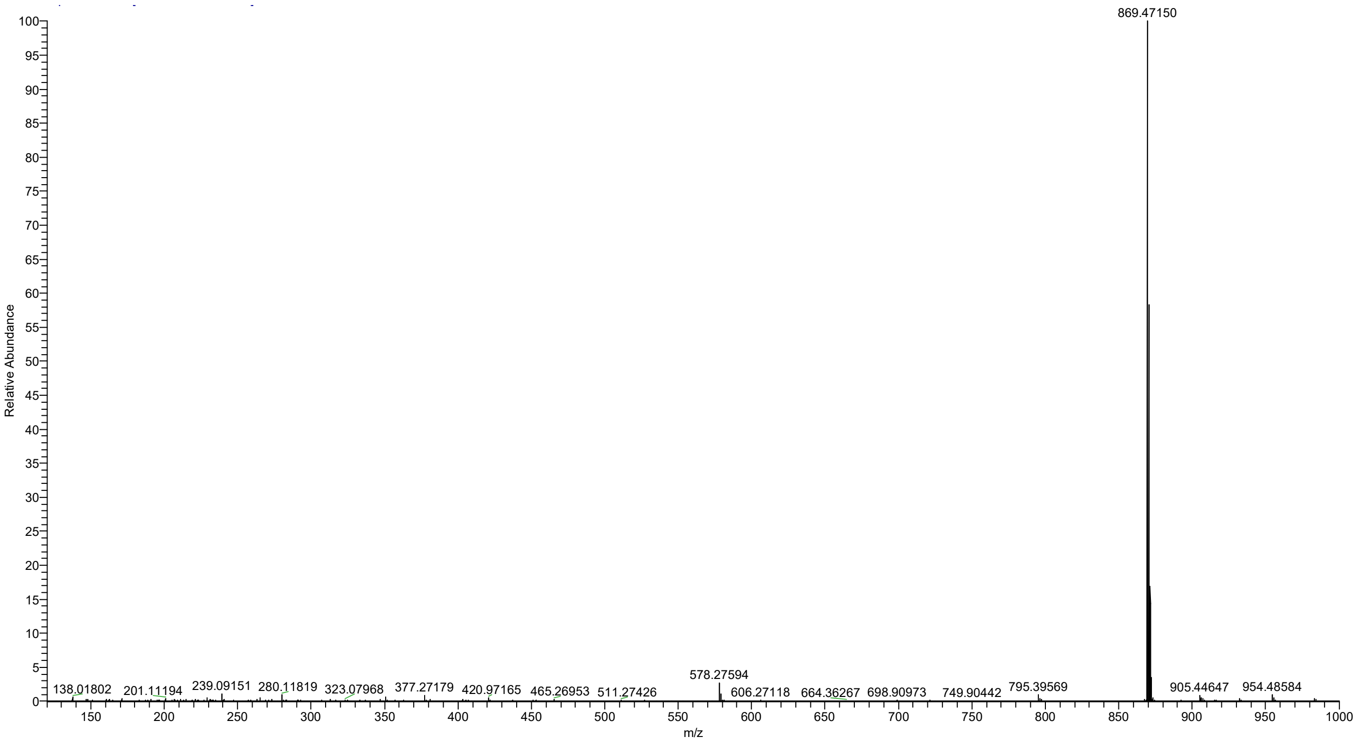

869.47150
Relative Abundance
m/z
138.01802
201.11194
239.09151
280.11819
323.07968
377.27179
420.97165
465.26953
511.27426
578.27594
606.27118
664.36267
698.90973
749.90442
795.39569
905.44647
954.48584

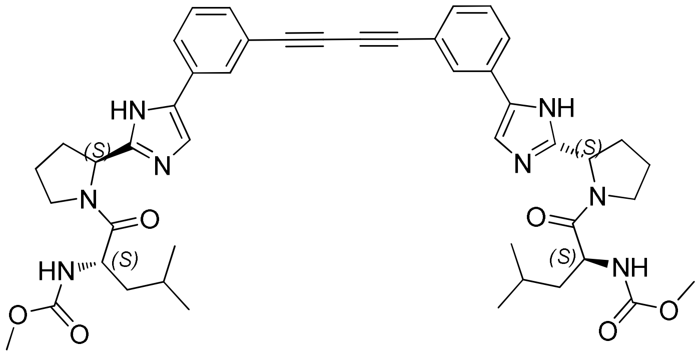
HN
N
(S)
N
O
HN
(S)
O
O
NH
N
(S)
N
O
(S)
NH
O
O

5a

HRMS

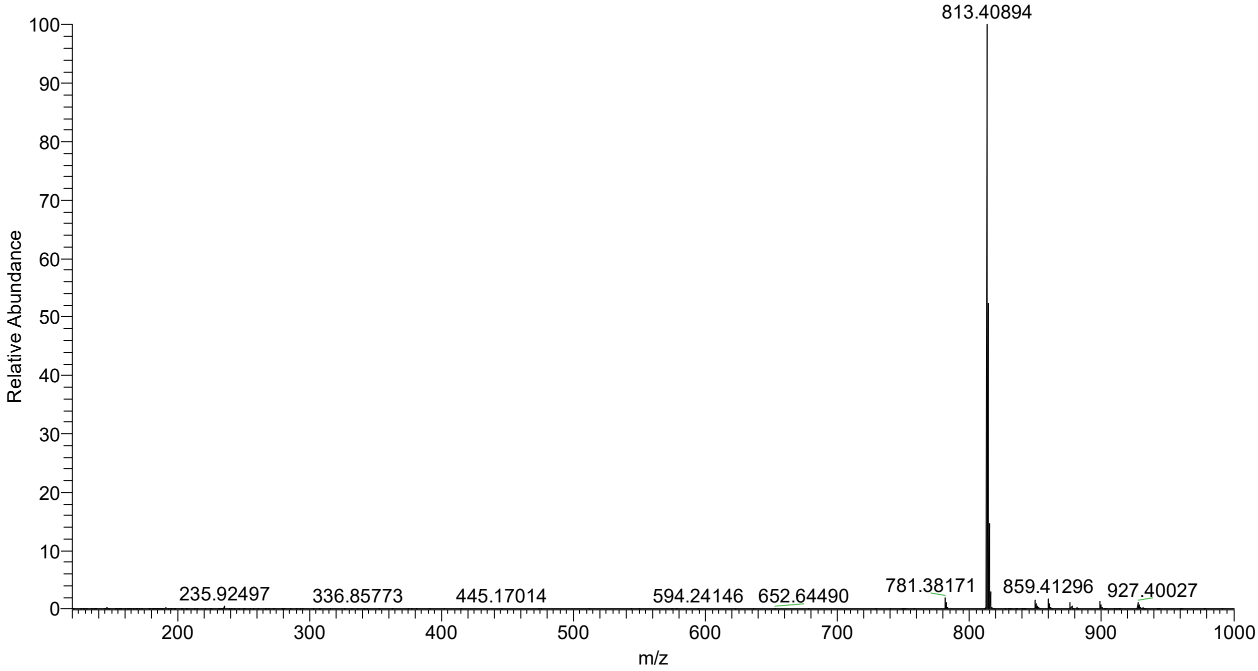
813.40894
235.92497
336.85773
445.17014
594.24146
652.64490
781.38171
859.41296
927.40027
Relative Abundance
m/z

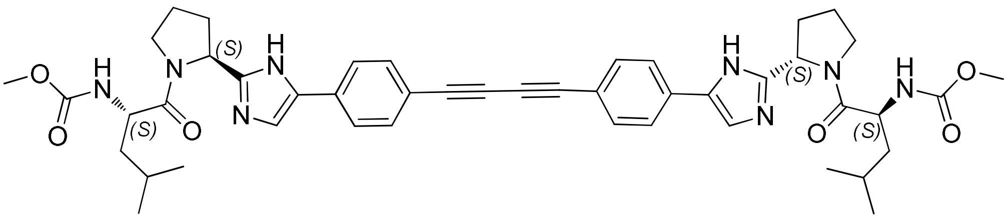
(S)
H
N
N
O
H
N
(S)
O
O
O
(S)
N
H
N
H
N
(S)
N
O
H
N
(S)
O
O
N

5b

HRMS

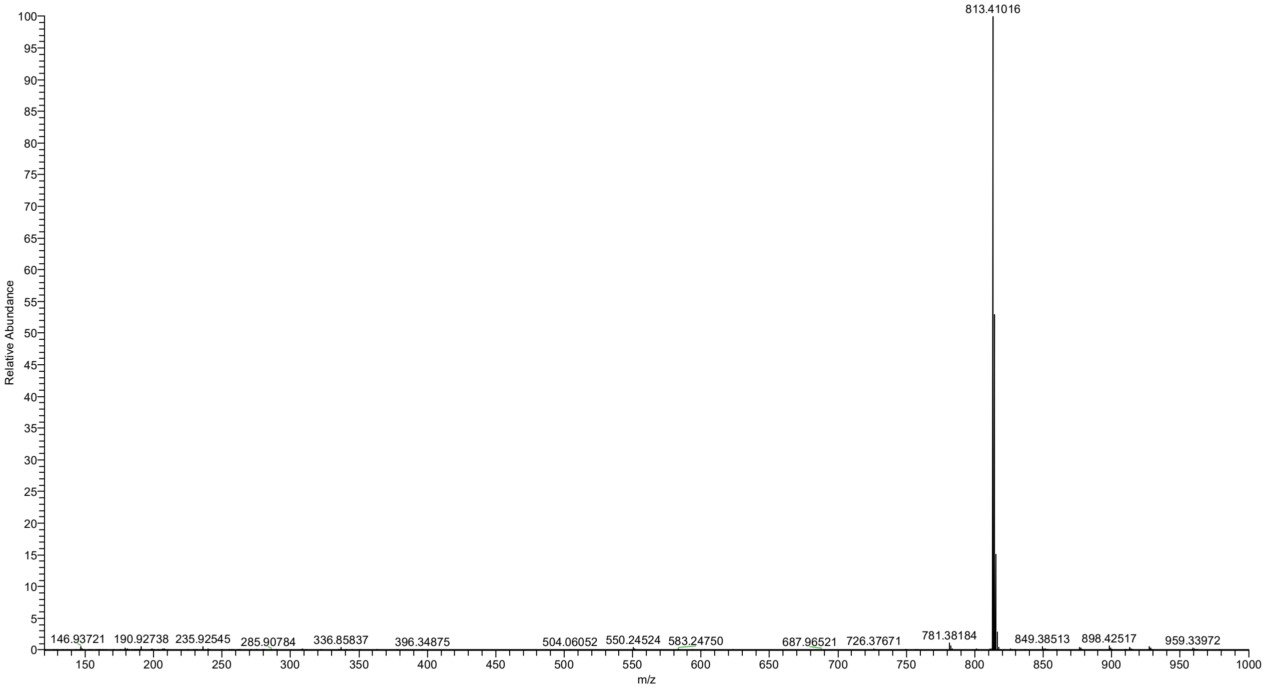
813.41016
Relative Abundance
146.93721
190.92738
235.92545
285.90784
336.85837
396.34875
504.06052
550.24524
583.24750
687.96521
726.37671
781.38184
849.38513
898.42517
959.33972
m/z

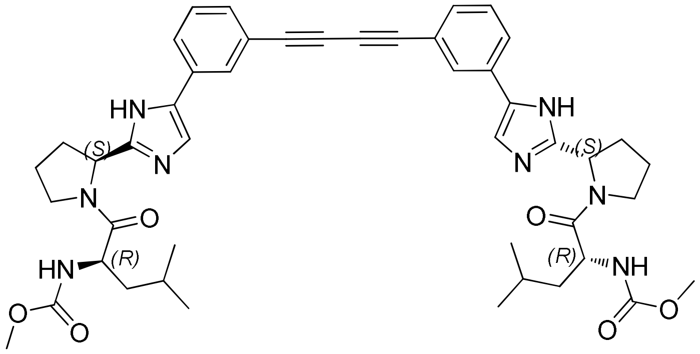
HN
N
(S)
N
O
HN
(R)
O
O
NH
N
(S)
N
O
(R)
NH
O
O

6a

HRMS

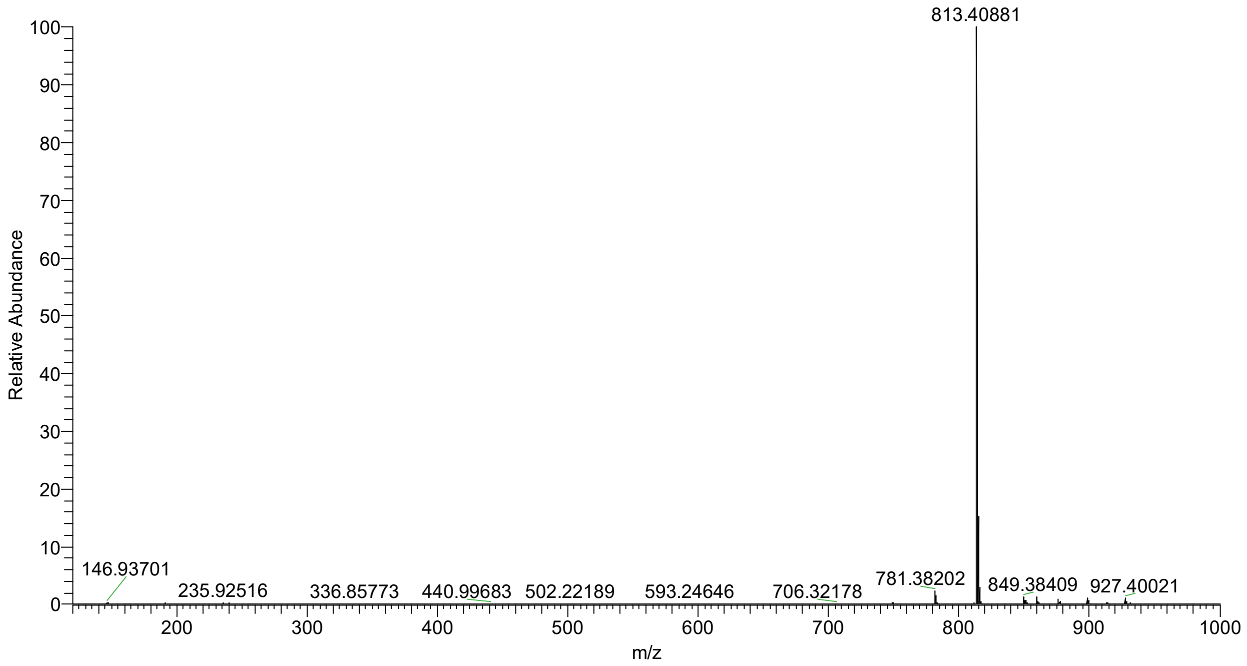
813.40881
100
90
80
70
60
50
40
30
20
10
0
Relative Abundance
146.93701
235.92516
336.85773
440.99683
502.22189
593.24646
706.32178
781.38202
849.38409
927.40021
200
300
400
500
600
700
800
900
1000
m/z

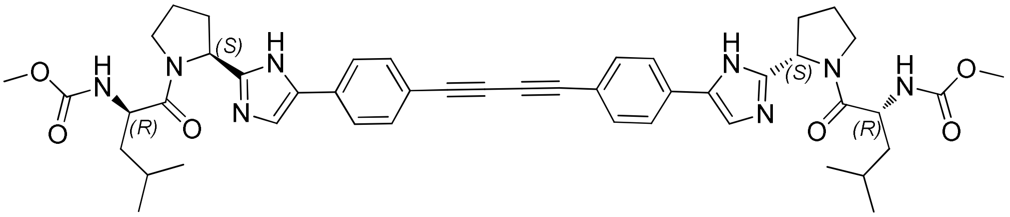
O
H
N
N
(S)
H
N
N
(R)
O
O
H
N
N
(S)
N
H
N
O
(R)
O
O

6b

HRMS

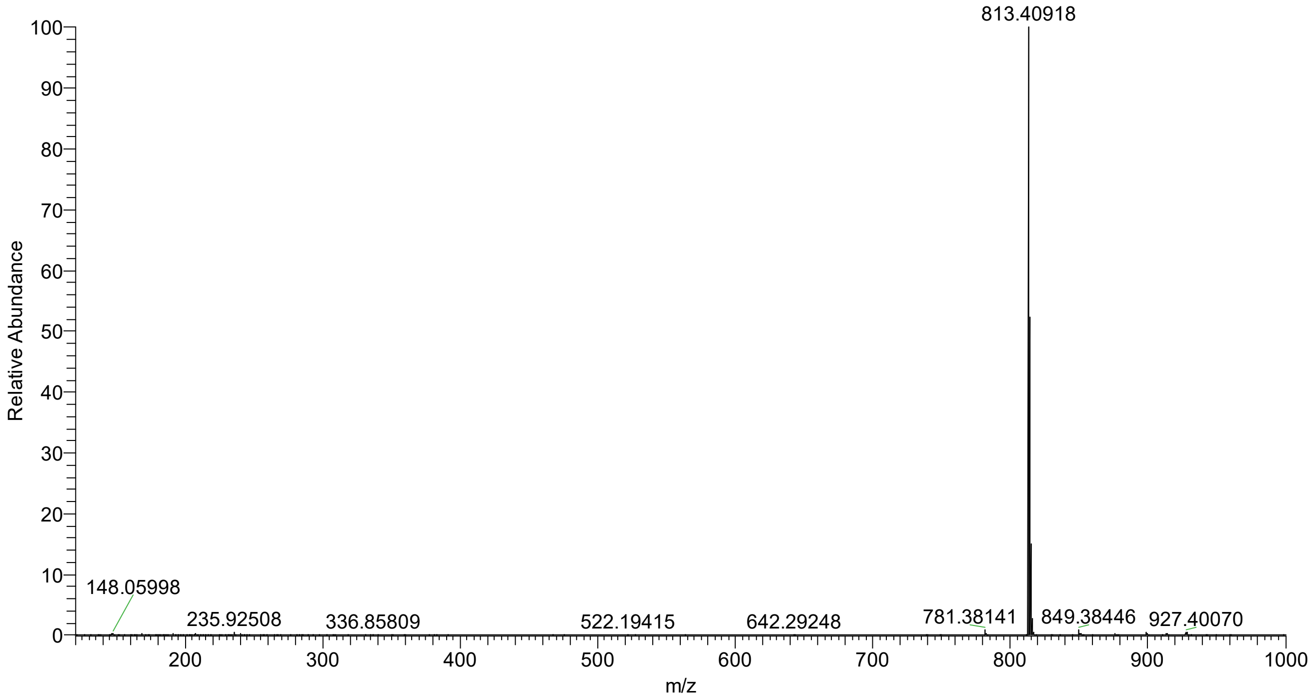
813.40918
Relative Abundance
148.05998
235.92508
336.85809
522.19415
642.29248
781.38141
849.38446
927.40070
m/z
0
10
20
30
40
50
60
70
80
90
100
200
300
400
500
600
700
800
900
1000

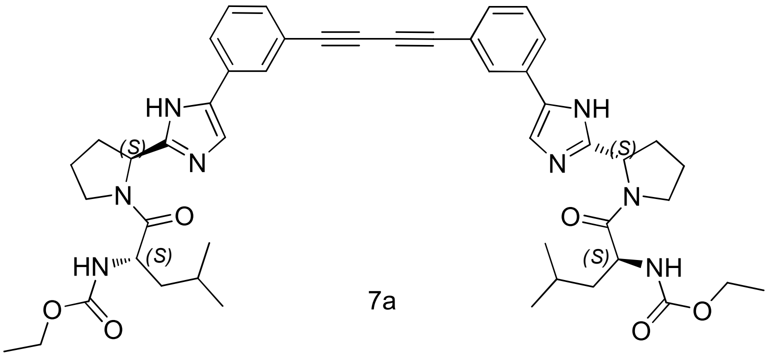
HN
N
(S)
N
O
HN
(S)
O
O
NH
N
(S)
N
O
(S)
NH
O
O

7a

HRMS

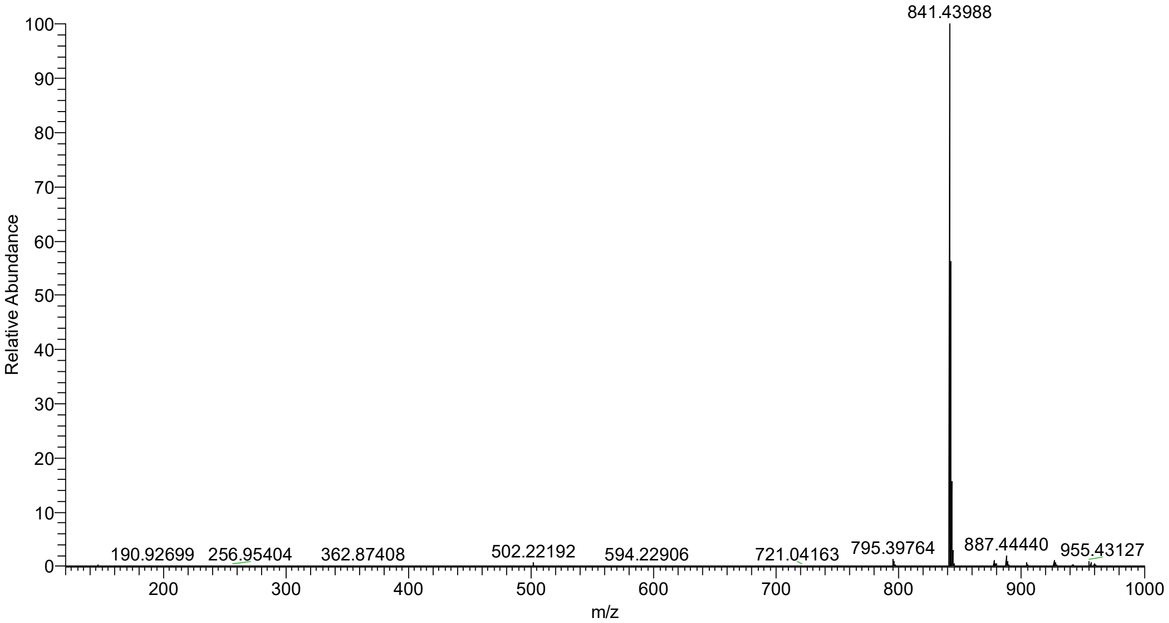
841.43988
100
90
80
70
60
50
40
30
20
10
0
Relative Abundance
190.92699
256.95404
362.87408
502.22192
594.22906
721.04163
795.39764
887.44440
955.43127
200
300
400
500
600
700
800
900
1000
m/z

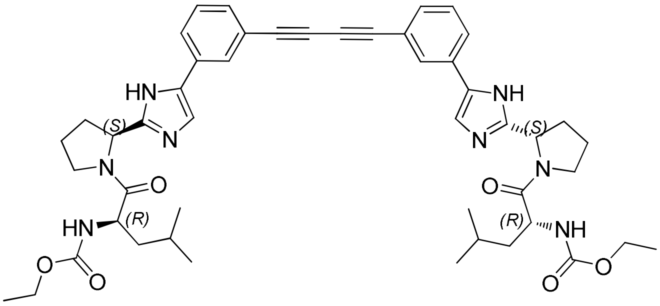
HN
N
(S)
N
O
HN
(R)
O
O
NH
N
(S)
N
O
(R)
NH
O
O

8a

HRMS

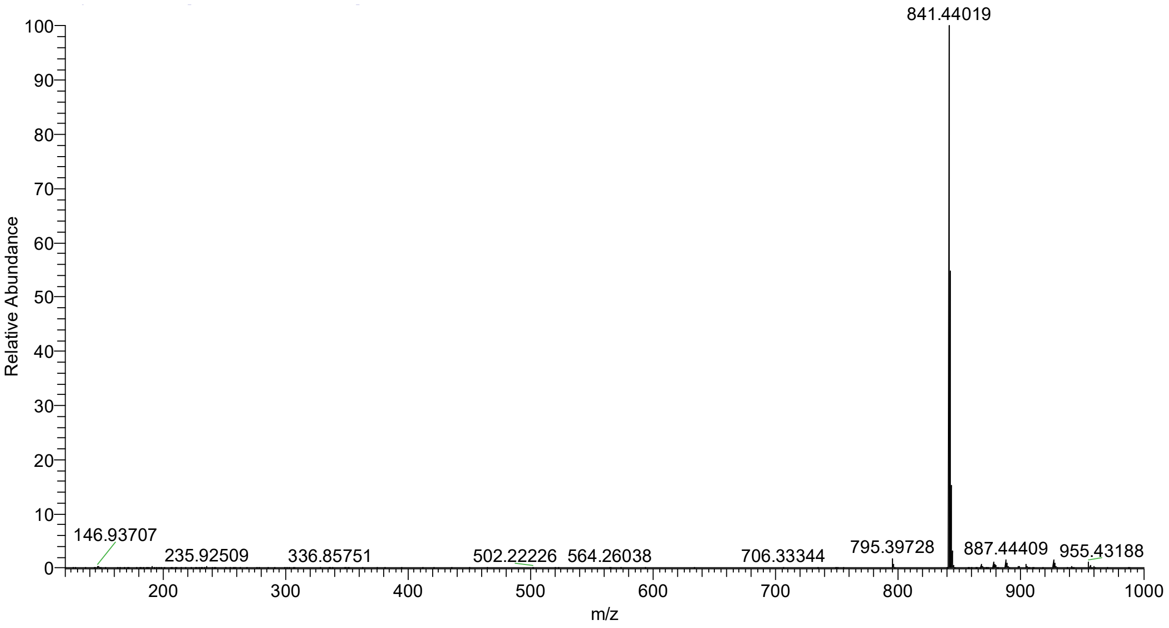
841.44019
Relative Abundance
146.93707
235.92509
336.85751
502.22226
564.26038
706.33344
795.39728
887.44409
955.43188
m/z

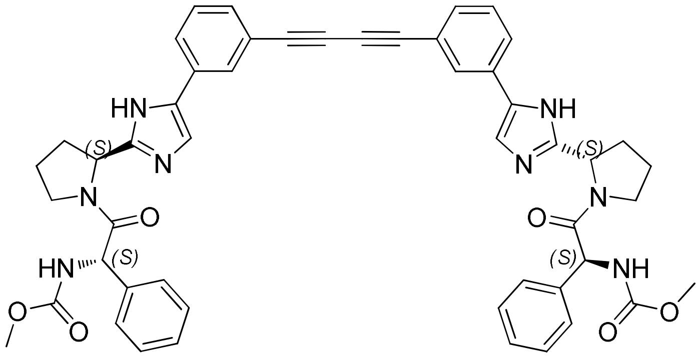
HN
NH
N
N
(S)
(S)
O
O
HN
NH
O
O
O
O

9a

HRMS

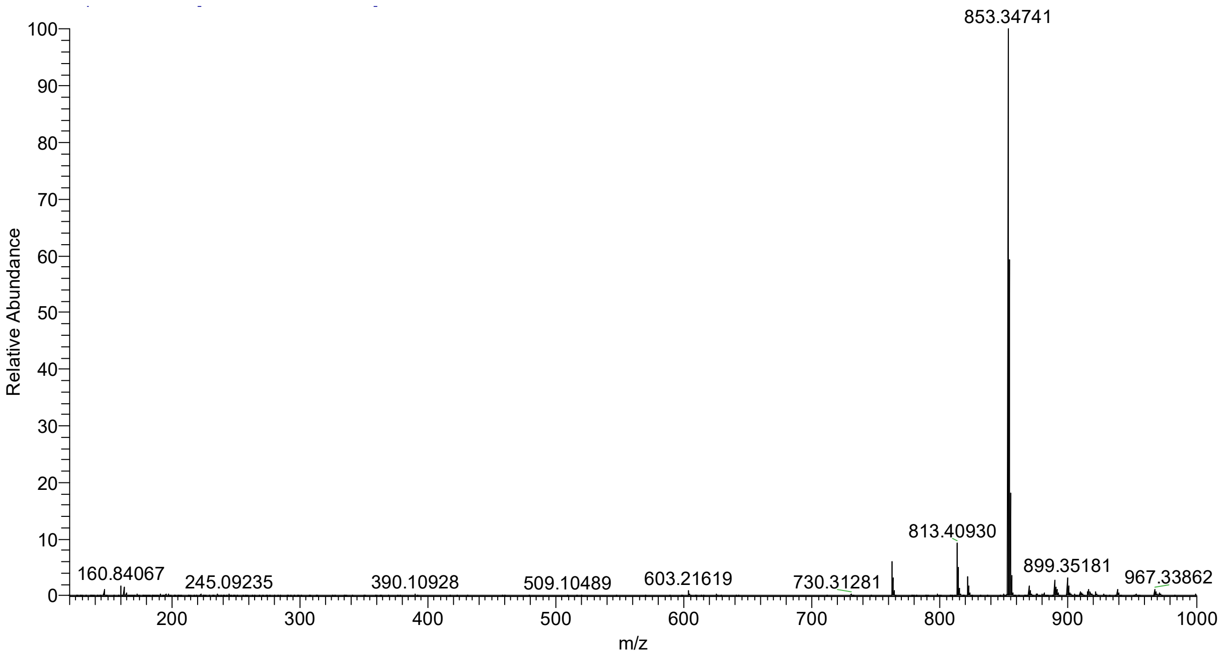
853.34741
813.40930
899.35181
967.33862
160.84067
245.09235
390.10928
509.10489
603.21619
730.31281
Relative Abundance
m/z

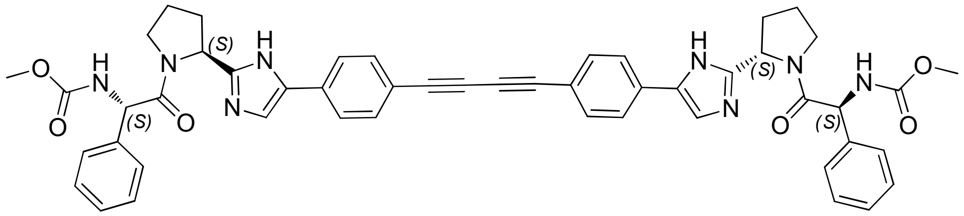
H
N
O
(S)
(S)
H
N
N
O
O
H
N
N
(S)
N
(S)
H
N
O
O
O

9b

HRMS

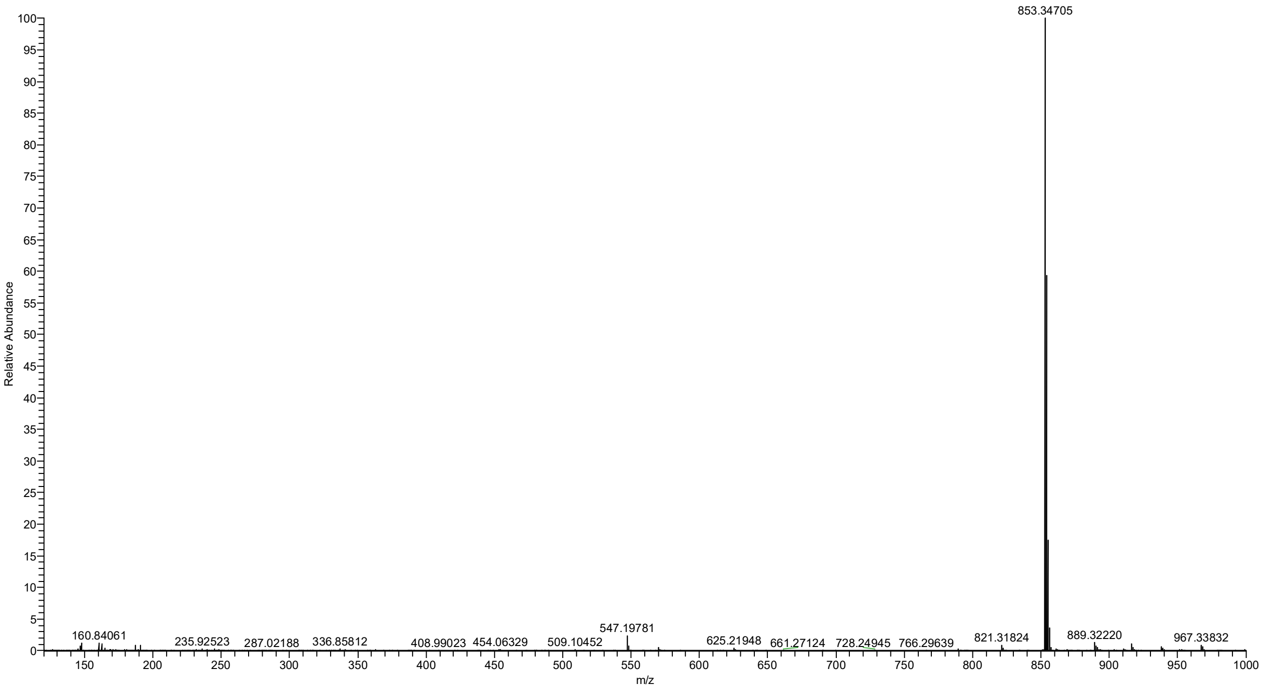
853.34705
Relative Abundance
160.84061
235.92523
287.02188
336.85812
408.99023
454.06329
509.10452
547.19781
625.21948
661.27124
728.24945
766.29639
821.31824
889.32220
967.33832
m/z

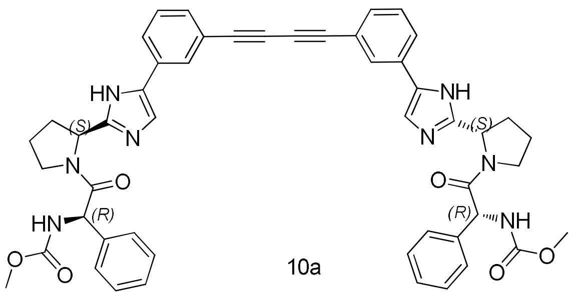

HN
NH
N
N
(S)
(S)
N
N
O
O
HN
NH
(R)
(R)
O
O
O
O

10a

HRMS

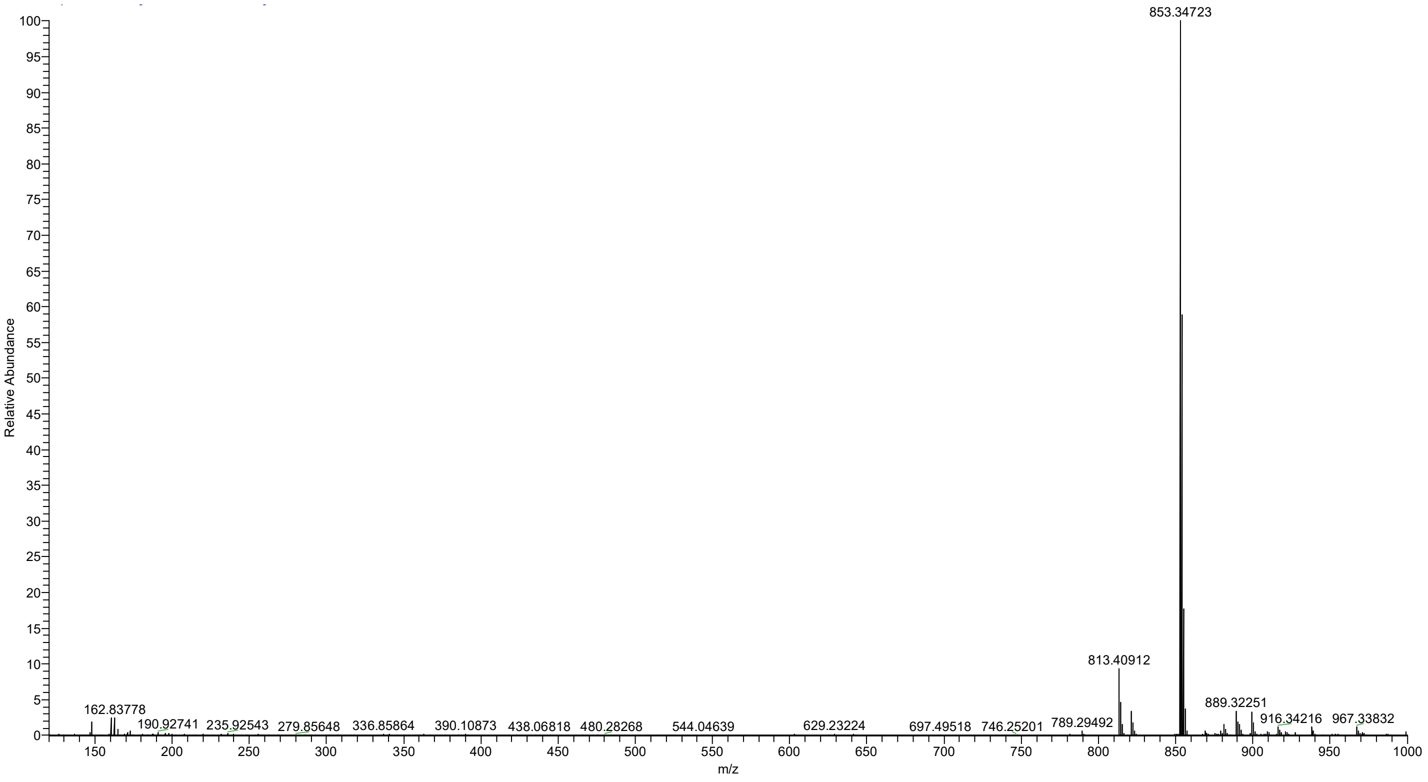

853.34723
813.40912
889.32251
916.34216
967.33832
162.83778
190.92741
235.92543
279.85648
336.85864
390.10873
438.06818
480.28268
544.04639
629.23224
697.49518
746.25201
789.29492
Relative Abundance
m/z

LC-MS

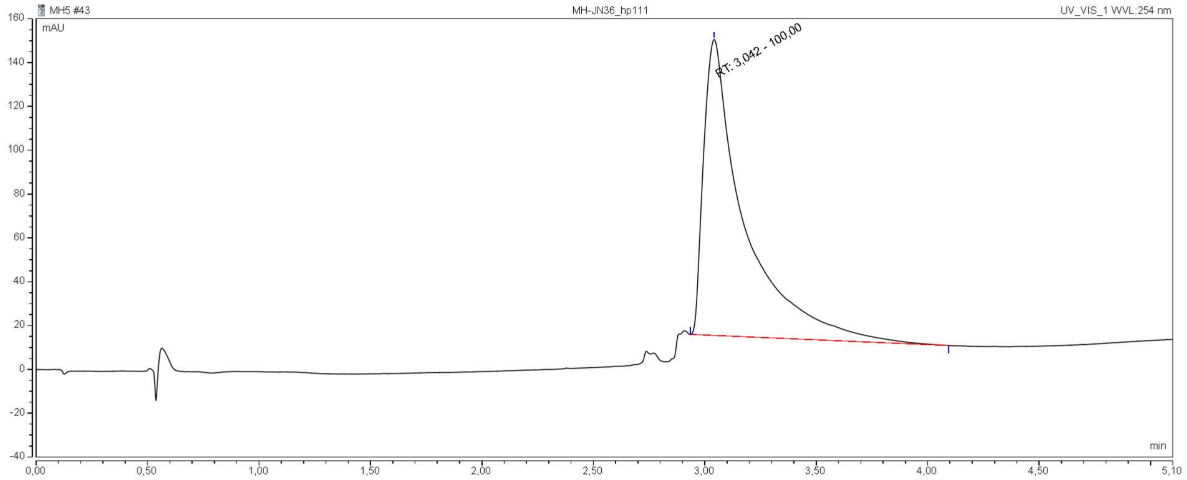
MH5 #43
MH-JN36_hp111
UV_VIS_1 WVL:254 nm
mAU
RT: 3,042 - 100,00
min

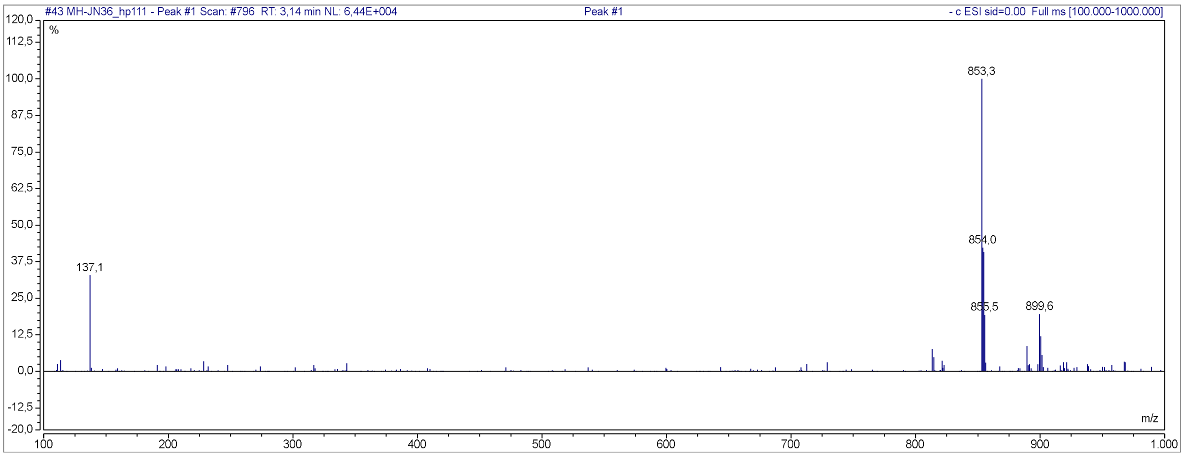
#43 MH-JN36_hp111 - Peak #1 Scan: #796 RT: 3,14 min NL: 6,44E+004
Peak #1
- c ESI sid=0.00 Full ms [100.000-1000.000]
%
853,3
854,0
855,5
899,6
137,1
m/z

^{1}H NMR

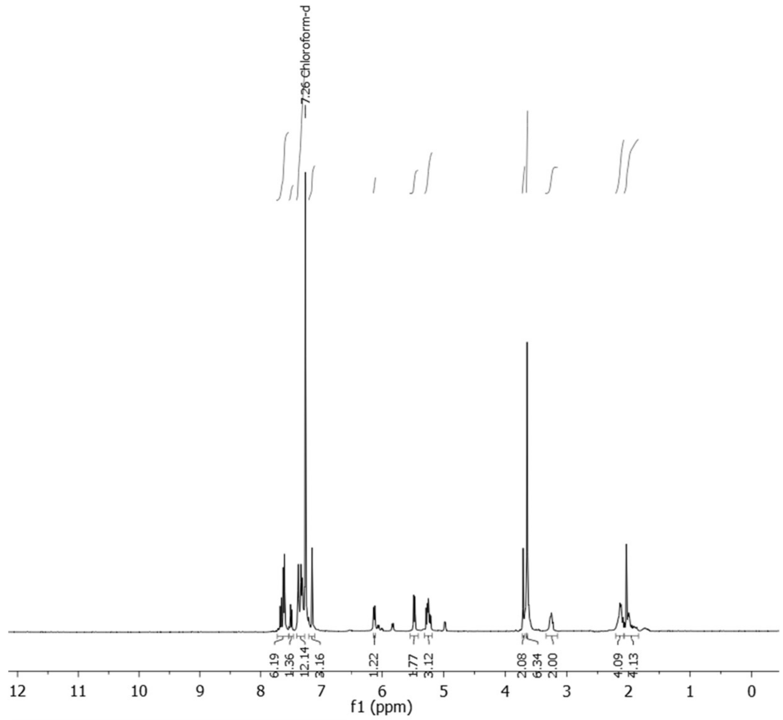

^{13}C NMR

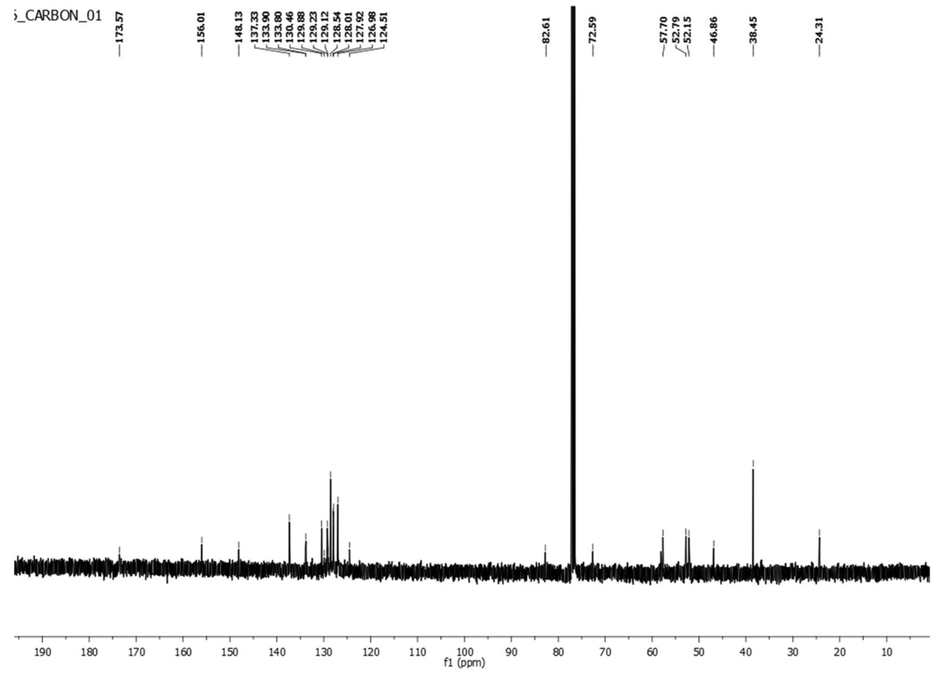

Supplement: Supplementary file 1 [file pharmaceuticals-15-00632-s001.zip › SI corrected.pdf]
